# Size‐Selective Functionalization of Sugars and Polyols Using Zeolites for Renewable Surfactant Production

**DOI:** 10.1002/anie.202511282

**Published:** 2025-08-15

**Authors:** Songlan Sun, Zezhong John Li, Yu‐Cheng Lin, Manon Rolland, Tom Nelis, Seongmin Jin, Shasha Zheng, Benjamin Nicolas Raffy, Wen Hua Bi, Esther Amstad, Jeremy S. Luterbacher

**Affiliations:** ^1^ Laboratory of Sustainable and Catalytic Processing Institute of Chemicals Sciences and Engineering Ecole Polytechnique Fédérale de Lausanne (EPFL) Lausanne CH‐1015 Switzerland; ^2^ Laboratoire des Polymères Institution of Materials Ecole Polytechnique Fédérale de Lausanne (EPFL) Lausanne CH‐1015 Switzerland; ^3^ Crystal Growth and Characterization Platform Institution of Physics Ecole Polytechnique Fédérale de Lausanne (EPFL) Lausanne CH‐1015 Switzerland; ^4^ Soft Materials Laboratory Institution of Materials Ecole Polytechnique Fédérale de Lausanne (EPFL) Lausanne CH‐1015 Switzerland

**Keywords:** Pore confinement, Renewable surfactants, Size‐selective acetalization, Sugars and polyols, Zeolites

## Abstract

Diol functionalization with acetals in carbohydrates and polyols is ubiquitously used in protection chemistry and in the manufacture of several platform chemicals. However, the selective functionalization of molecules where multiple acetalization reactions can occur typically involves multiple protection/deprotection steps. Here, we show that zeolites can be used to size‐selectively acetalize sugars and polyols in a single step, and we demonstrate the potential of this approach for producing functional chemicals and materials. We show that high selectivity is dependent on effective pore confinement, which is achieved by a careful pairing of the substrate size and the zeolite morphology. This zeolite‐catalyzed strategy exhibited consistently good‐to‐excellent yields (70%–95%) of novel polyol/sugar monoacetals, notably offering an easy route to bio‐based amphiphiles. These resulting xylose‐based surfactants exhibited similar or superior surface activity and, remarkably, hard‐water resistance compared to their nonrenewable ethoxylated analogues, which illustrates the potential for practical applications of this approach.

## Graphical abstract

A zeolite‐catalyzed strategy enables size‐selective monoacetalization of sugars and polyols in a single step by leveraging pore confinement to distinguish between mono‐ and multi‐substituted species. This broadly applicable method avoids protection steps and excess reagents, opening new routes to bio‐based amphiphiles with strong surface activity and hard‐water resistance.

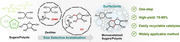

## Introduction

**Figure 1:**
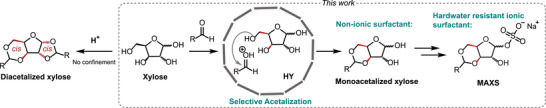
Comparison of confined zeolite‐catalyzed acetalization with the homogeneous acid‐catalyzed reaction: Monoacetalization is achieved via the size‐selective attribute of zeolites; in the absence of confinement, xylose forms multiple low ring‐strain cyclic acetals (“*cis*” ring junction).

Sugar and polyol acetalization play a pivotal role in crafting important molecular architectures, notably through protection group chemistry.^[^ ^1^ ^]^ Applications that require selective acetalization include surfactants,^[^ ^2^ ^]^ polymer precursors,^[^ ^3^ ^]^ and pharmaceutical intermediates.^[^ ^4^, ^5^, ^6^ ^]^ Typically, the synthesis processes involve unselective protection followed by selective deprotection;^[^ ^7^ ^]^ or the careful manipulation of reaction conditions, such as slowing down the reaction rate,^[^ ^8^ ^]^ regulating the hydrolysis equilibrium by adding water,^[^ ^9^ ^]^ or introducing a large excess of one of the reactants.^[^ ^2^, ^10^ ^]^ However, such processes suffer from poor atom economy and high process complexity. For instance, monoacetalized erythritol and xylitol act as precursors for nonionic,^[^ ^2^ ^]^ or anionic^[^ ^10^ ^]^ surfactants but are produced through the use of a large excess of polyols or by carefully controlling the hydrolysis equilibrium through water content optimization. These examples illustrate the lack of selective and atom‐efficient syntheses for selective acetalization, notably for sugars and polyols—such as xylose, glucose, erythritol, xylitol, and sorbitol—which often result in the formation of thermodynamically stable diacetals (Figure 1). At least partially for this reason, the acetalized version of these molecules has not been extensively used in the production of commodity chemicals, despite their ubiquity in the renewable chemical space. Such molecules are used less than those that are easy to produce selectively due to their formation of only one low‐strained ring acetal. Sorbitan acetals are a good illustration of this effect and are used extensively in surfactant preparation.^[^ ^11^ ^]^ Similar examples include xylulose and lyxose, which can selectively form monoacetals, as the creation of a second cyclic acetal is hindered due to high ring‐strain or high steric hindrance (Figure S1). However, the low natural occurrence of some of these substrates hinders their wider use.

Despite the use of enzymatic catalysts such as glycosidases or lipases in regio‐ and stereoselective transformations of carbohydrates, most notably in alkyl glycosides^[^ ^12^ ^]^ formation (acetal formation at the anomeric center), selective esterification,^[^ ^13^, ^14^, ^15^, ^16^ ^]^ and selective acylation/deacylation,^[^ ^17^, ^18^ ^]^ we are not aware of any enzymatic system being reported that catalyzes cyclic acetal formation from polyols or sugars. Likewise, homogeneous organic catalysts, such as chiral phosphoric acids,^[^ ^19^, ^20^ ^]^ boron‐based Lewis acids,^[^ ^21^ ^]^ have been used to chemoselectively and stereoselectively functionalize a single hydroxyl group in diols, but no system has been reported for the direct and selective formation of cyclic acetals from unprotected polyols.

Aluminosilicate zeolites are acid catalysts known to catalyze a variety of transformations, including alkylation, isomerization, cracking, dehydration, and condensation reactions. Acetalization, a subset of condensation reactions, has been explored with zeolites,^[^ ^22^, ^23^, ^24^, ^25^, ^26^, ^27^, ^28^ ^]^ but reported examples are exclusively non‐size‐selective. Notably, these studies focused on acetalization involving furfural‐related and glycerol‐related compounds for the synthesis of bio‐based fuel additives, where selectivity was not crucial for the product functions.

Zeolites are solid crystalline catalysts with active sites within molecular‐sized pores, the dimensions of which can control the product distribution. When reactions occur under confinement, constriction can control the size and shape of the resulting products. Typical examples include *para*‐selective alkylation of toluene over *meta‐* and *ortho*‐substitutions^[^ ^29^ ^]^ and cavity‐controlled methanol‐to‐olefins processes.^[^ ^30^ ^]^ More recent studies showcased the diverse applications of zeolites in the selective conversion of bio‐based molecules. For example, Dusselier et al. achieved the controlled dimerization of L‐lactic acid to (L, L)‐lactide using a BETA zeolite, preventing oligomerization, which led to a useful monomer for ring‐opening polymerization to produce high‐molecular‐weight polylactic acid (PLA).^[^ ^31^ ^]^ Recently, the same group developed the synthesis of bisphenol A alternatives, such as *p,p′*‐bisguaiacol, through a selective alkylation reaction between bio‐based arenes and alkenes using zeolite USY.^[^ ^32^ ^]^ More examples include the cross‐etherification of hydroxymethylfurfural (HMF) with fatty alcohol instead of self‐etherification by imposing geometric constraints with BETA zeolites to produce bio‐based surfactants,^[^ ^33^ ^]^ or glucose pyrolysis over medium‐pore zeolites to improve selectivity toward aromatics over oxygenate compounds and coke.^[^ ^34^ ^]^ Moreover, the pore architecture of zeolites not only affects the formation of products according to size and form but also governs molecular adsorption and diffusion,^[^ ^35^, ^36^ ^]^ promoting reactant aggregation in constrained environments or limiting reactants due to slow diffusion, ultimately influencing reaction rates and selectivity.

In this work, we show that the size‐selective attribute of zeolites can be used to catalyze acetalization and achieve selective functionalization by exploiting the substantial difference in molecular size between mono‐substituted and multi‐substituted substrates. To understand these systems, we explored a broad spectrum of polyols and sugars as substrates, different aldehydes as functionalization reagents, and a wide range of zeolites. With optimal adjustments, this proposed zeolite‐catalyzed selective acetalization strategy demonstrated consistently good‐to‐excellent yields of sugar/polyol monoacetal, without the need for multiple functionalization/defunctionalization steps^[^ ^7^ ^]^ or excessive use of one of the reactants.^[^ ^2^ ^]^ As a proof of concept, we demonstrate that this approach can be used to make several nonionic and anionic surfactants with remarkable properties that are comparable to or exceed those of major fossil‐based surfactants.

## Results and Discussion

**Figure 2:**
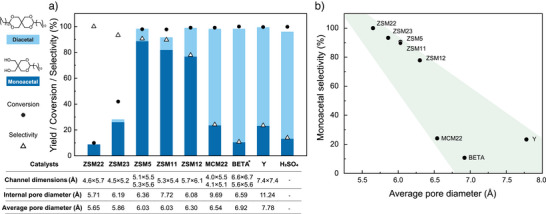
Pentaerythritol acetalization with dodecanal. a) Product distributions using different zeolites and H_2_SO_4_. b) Monoacetal (monododecylidene‐pentaerythritol) selectivity as a function of average pore diameter for different zeolites. Reaction conditions: 65 °C, 5 h, 1:2 mol. equivalent (polyol/dodecanal), zeolite: 0.05 g mL^−1^, H_2_SO_4_: 0.02 mol L^−1^. Selectivity was defined as the monoacetal product over the total monoacetal and diacetal products (no other products were observed) (Equation S6). The zeolite channel and internal pore dimensions in (a) were obtained from IZA (International Zeolite Association) database.^[^ ^39^ ^]^ The channel dimensions refer to the size of the openings between cavities, while the internal pore diameter represents the size of the internal voids within the zeolite. The average pore diameters in (a) and (b) were experimentally determined by Ar isotherm physisorption at 77 K with the nonlocal density functional theory model. The full pore size distributions are given in Figure S23. Table S10 contains more detailed information on these zeolites. *The BETA zeolite has a disordered framework structure, but the dimensions of the A polymorph are shown in panel (a) as representative values. The shaded areas serve only to highlight the trend in the data.

To demonstrate the influence of the pore size on the reaction selectivity, we investigated whether there was a correlation between monoacetal selectivity/yield and the zeolite pore dimensions. We used a range of zeolite pore sizes that spanned from 10‐membered‐ring (10‐MR) medium‐pore microporous zeolites (ZSM22, ZSM23, ZSM5, ZSM11, and MCM22) to 12‐membered‐ring (12‐MR) large‐pore microporous zeolites (ZSM12, BETA, and Y). We compared the results of these heterogeneous acid catalysts to those of the mineral acid H_2_SO_4_. We chose pentaerythritol, a symmetric tetraol with a kinetic diameter of 6.0 Å, as the first substrate to limit the complexity of substrates and eliminate product isomerism. The best monoacetal‐to‐diacetal selectivities were achieved primarily using 10‐MR zeolites (Figure 2a). The maximum monoacetal yield of 88.9% was obtained with ZSM5, a zeolite with an intersecting 10‐MR pore system composed of straight (5.3 × 5.6 Å) and sinusoidal (5.1 × 5.5 Å) channels. This result was followed by ZSM11 and ZSM12, which gave 82.0% and 78.5% monoacetal yield, respectively. Among the three, ZSM5 and ZSM11 possess a 3D pore structure with medium‐sized pore windows, which facilitates substrate diffusion to the active sites while providing enough confinement to suppress diacetalization, thus enhancing monoacetalization selectivity. ZSM12, despite being a 12‐MR zeolite, possesses a one‐dimensional pore system and a relatively small internal pore space, which effectively promotes confinement and favours the formation of monoacetal as well. If the pore size of one‐dimensional zeolite was too small for the substrate, as was the case in ZSM22 and ZSM23, the conversions remained low (below 50% at these reaction conditions), but the selectivity remained over 90%. This low conversion could likely be attributed to the severe mass transfer limitation and fouling (pore mouth blockage), while the pore confinement still limited the formation of a second acetal. 12‐MR large‐pore 3D microporous zeolites (BETA and Y) showed lower monoacetal selectivity, leading to results that resembled those observed with H_2_SO_4_. This similarity indicates a lack of influence of the pore confinement on this open‐chain polyol within the large‐pore microporous framework. Overall, monoacetal selectivity is negatively correlated to zeolite average pore diameter with a statistical significance of 99.95% (Figure 2b). Correlations with other zeolite properties, including Brønsted acid site loading, specific surface area, and crystallite size, did not show any statistical significance (Figure S24, Table S11). The Lewis acid sites were found not to be the active site of these reactions (discussed below in Figure 4b), so no correlations were attempted. Although acid strength is known to influence reaction rates and product selectivity by modulating reaction kinetics, this effect is not prominent in this study, likely due to diffusion limitations that dominate over intrinsic reactivity.^[^ ^37^ ^]^ Zeolites with weaker acid sites, such as Y and Beta, as determined by NH_3_‐TPD (Figure S21), showed similar monoacetal selectivity and substrate conversion as the strong mineral acid H_2_SO_4_, whereas zeolites with greater acid strengths (e.g., ZSM23 and ZSM5) predominantly yielded monoacetal. This reaffirms the prominence of pore confinement on the reaction selectivity. Interestingly, despite having a similar pore size to ZSM5 (where maximum pore diameters are all close to 5.5 Å^[^ ^34^ ^]^), MCM22 exhibits a distinct preference for diacetal formation over monoacetal formation, with yields comparable to those observed in large‐pore zeolites. MCM22 has a combination of 10‐ and 12‐membered‐ring channels with the large 12‐MR cavities open to the exterior. The diffusion resistance and steric hindrance of reactants and products are much reduced in these large open cavities (7.1 Å in diameter and 18.2 Å in height) compared to that of zeolites with 10‐membered rings only. Similar trends were observed for MCM22 with different reagents and products as well.^[^ ^38^ ^]^ These results suggest that the substrate conversion and product selectivity are affected by an interplay between pore window sizes, the number of dimensions of the accessible zeolite channels, channel dimensions, and the internal pore sizes.

**Figure 3:**
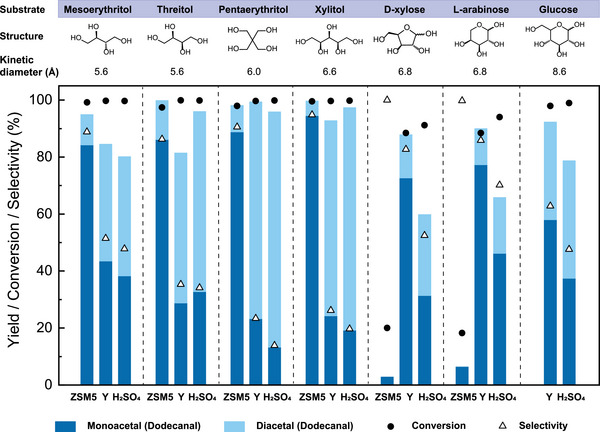
The product distribution of various polyol/sugar acetalizations catalyzed by zeolites and sulfuric acid. High selectivity toward monoacetals using appropriately sized zeolites showed that size‐selective acetalization with zeolites is widely applicable. Reaction conditions: 65 °C, 5 h, dioxane, 1:2 mol. equivalent (polyol/aldehyde), zeolite: 0.05 g mL^−1^ (SiO_2_/Al_2_O_3 _= 80). H_2_SO_4_: 0.02 mol L^−1^. Here, the different isomers of mono‐ and diacetals are not differentiated for simplicity. Further structural details for acetalized xylose are presented in Figure 4. The estimations of substrate kinetic diameters are detailed in the Supporting Information, Section 6.

To explore the universality of the pore confinement effect of zeolites on acetalization, we reacted substrates of various sizes with dodecanal, catalyzed by either medium‐pore microporous zeolite ZSM5, large‐pore microporous zeolite Y, or the mineral acid H_2_SO_4_ (Figure 3). Catalyzed by ZSM5, open‐chain polyols (mesoerythritol, threitol, pentaerythritol, and xylitol) led to high monoacetal yields, ranging from 84% to 95%. In contrast, we did not see high sugar conversion with cyclic sugars (D‐xylose and L‐arabinose) under these conditions but observed excellent selectivity for monoacetals, exceeding 99%. D‐Glucose was not tested with ZSM5, for its large kinetic diameter (8.6 Å) exceeded the pore dimensions of the zeolite (4.7 Å). Switching to the large‐pore HY zeolite, reactions with five‐carbon cyclic sugars (D‐xylose and L‐arabinose) led to higher conversion and favourable monoacetal yields (ranging from 70% to 80%), especially compared to H_2_SO_4_. With this larger zeolite, the high selectivity of monoacetals observed in open‐chain polyols diminished and tended toward that of H_2_SO_4_. This result further confirmed the effect of substrate confinement in zeolite pores. The smaller open‐chain polyols experienced a stronger steric effect in ZSM5 pores compared to those of HY, resulting in a higher yield of the smaller monoacetals. The steric effect vanished for these smaller polyols when the large‐pore HY was used, but remained for larger cyclic sugars. At the same time, these larger cyclic sugars experienced high diffusion resistance through the small ZSM5 pores (Table S13), which explains the low sugar conversions with these catalysts. The poorer mole balance of these cyclic sugars catalyzed by ZSM‐5 compared to HY further suggests that some sugars and their acetals (or degradation products) could not be removed from the pores after repeated washing, an indicator of pore blockage. In summary, the selectivity for monoacetal formation varies for each substrate, depending on its relative size when paired with a particular microporous catalyst. The kinetic diameter of substrates serves as a valuable indicator for gauging their relative sizes, but cannot provide an exact measurement of a molecule's precise dimensions. The challenge in defining the precise size effects of a molecule likely arises from the complexities associated with non‐spherical shapes and dynamic conformations. We use only the substrate diameter, as the aldehyde is constant and acts as a narrow tail, while the sugar or polyol dominates the overall size as the bulky head group.

We studied more extensively the acetalization of xylose due to the simplicity of its reaction combined with its very high natural abundance—xylose is derived from hemicellulose and is the second most prominent carbohydrate on the planet after glucose.^[^ ^40^ ^]^ We chose 12‐MR large‐pore microporous zeolites such as BETA and Y to investigate the factors affecting the distribution of xylose acetalization products, notably because 10‐MR zeolites showed limited product yields with xylose (Table S15).

**Figure 4:**
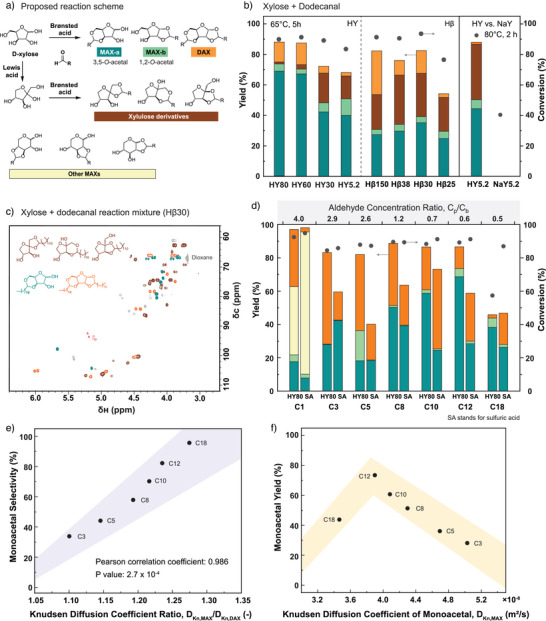
Xylose acetalization reaction. a) Proposed reaction scheme for xylose acetalization and legend. b) Acetalization reaction of xylose and dodecanal over Y and BETA zeolites with different SiO_2_/Al_2_O_3_ ratios. c) HSQC 2D NMR spectrum of the reaction mixture of xylose and dodecanal catalyzed by Hβ30. d) MAX (monoalkylidene‐xylose or monoacetalized xylose) and DAX (dialkylidene‐xylose or diacetalized xylose) yield from acetalization reactions of xylose and different aliphatic aldehydes (from C1 formaldehyde to C18 octadecanal) over HY80 zeolite and H_2_SO_4_. Correlations between e) monoacetal selectivities, f) monoacetal yields during D‐xylose acetalization with various aldehydes using HY80, and the corresponding Knudsen diffusion coefficients for the reaction products in the zeolite pores. For simplicity, zeolite types are prefixed with the cation type (H for proton and Na for sodium) and suffixed with the SiO_2_/Al_2_O_3_ ratio. Reaction conditions were: 65 °C, 5 h, 1:2 mol. equivalent (xylose/aldehyde), zeolite: 0.05 g mL^−1^. H_2_SO_4_: 0.02 mol L^−1^.

In such xylose acetalization reactions, we frequently observed the formation of xylulose derivatives, in addition to monoacetalized (MAX) and diacetalized xylose (DAX), as identified through HSQC spectra (Figures 4a–c and S30). These xylulose derivatives likely originated from xylose isomerization catalyzed by Lewis acids,^[^ ^41^ ^]^ followed by xylulose acetalization reactions catalyzed by Brønsted acids (Figure 4a). In particular, the use of BETA zeolites and Y zeolites, which had lower SiO_2_/Al_2_O_3_ ratios, led to higher yields of xylulose derivatives due to their higher density of Lewis acid sites (Table S5). In cases where only weak Lewis acid sites were present, neither MAX nor DAX was formed, and no other products were detectable by gas chromatography (GC), as shown by the comparison between HY5.2 and NaY5.2 (Figure 4b). As a much stronger Lewis acid,^[^ ^42^ ^]^ AlCl_3_ alone was also found inadequate to catalyze xylose acetalization (Table S17). Although Lewis acid sites have been reported to catalyze the acetalization of open‐chain polyols (e.g., glycerol),^[^ ^43^, ^44^ ^]^ the hydroxyl groups on a sugar ring likely exert sufficient additional strain to render Lewis acid sites ineffective. Since Lewis acid sites were only found to catalyze the undesirable side reactions, one may be able to remove them by post‐synthesis treatments such as strong acid wash, chelating agent extraction, or chemical reactions to remove the extra‐framework aluminum species.^[^ ^45^, ^46^, ^47^, ^48^ ^]^ However, these treatments could also inadvertently affect framework stability and Bronsted acid sites, so optimization may be needed for each type of zeolite. ^[^ ^47^ ^]^

To investigate the effects of aldehyde size and electrophilicity on product selectivity, HY80, which has lower Lewis acid density, was chosen to eliminate the influence of xylose isomerization. We observe that MAX formation becomes increasingly favoured with larger aldehyde sizes (Figure 4d). Given the observed pore confinement effects, the product molecules (monoacetals and diacetals) are likely comparable in size to the zeolite pores, making Knudsen diffusion dominant; therefore, Knudsen diffusion coefficients were calculated to quantify the diffusion resistance they experience in the pores. Additionally, calculating Knudsen diffusion coefficients requires only molecular weight instead of molecule geometry, removing the difficulty in defining their exact kinetic diameters. The Knudsen diffusion coefficients were calculated with the experimentally measured average pore size for each zeolite‐product pair (Tables S12–14). We observe a monotonic increase in the MAX selectivity with an increasing Knudsen diffusion coefficient ratio of MAX to DAX, reaffirming the role of transport limitation in controlling the selectivity (Figure 4e). We further observe clear correlations between the yields of xylose monoacetals and diacetals and their diffusion coefficients within the HY80 pores (Figures 4f and S25). The DAX yield was positively correlated with the diffusion coefficients. Specifically, the lower the diffusion coefficient a product had, the lower the yield was measured, likely due to limitations in their formation within or diffusion out of the zeolite pores. As expected, the trend for MAX yield versus diffusion coefficient is non‐monotonic and features a maximum (Figure 4f). When diffusion coefficients were too low, the products could not access or form within the pores; when too high, size‐selective effects were insufficient to hinder diacetal formation, leading to an optimum intermediate diffusion coefficient. Similarly, the selectivity of pentaerythritol acetalization with zeolites of various pore sizes could also be correlated with Knudsen diffusion coefficients of the respective mono‐ and diacetals (Figure S26), where zeolites with small pores exerted diffusion restrictions on diacetals and favoured the formation of monoacetals. However, when the pore was too small, the monoacetal yield decreased due to limited substrate accessibility.

We also noted that smaller aldehydes (<C8) catalyzed by HY80 produced higher DAX yields compared to H_2_SO_4_, highlighting another characteristic of zeolite architecture: reactant aggregation in confined environments. The increased yield of DAX with smaller aldehydes was thus mainly attributed to the partition coefficient (*C*_p_/*C*_b_), defined as the ratio of aldehyde concentration within the zeolite pores (*C*_p_) to the bulk solution (*C*_b_). The *C*_p_/*C*_b_ decreased from 4.0 to 0.5 as the aldehyde size increased from formaldehyde to octadecanal, causing a shift in the primary product from DAX to MAX due to equilibrium considerations. Additionally, unsaturated functional groups in aldehydes altered the partition coefficient due to π‐interactions with Lewis acid sites within the catalyst, resulting in greater adsorption compared to saturated aldehydes of the same chain length (e.g., 2‐ and 4‐dodecenal versus dodecanal, Table S16, entries 7, 9, and 10), which also enhanced the DAX yields compared to their respective homogeneous systems. However, concentration alone does not dictate product distribution, as decreasing the aldehyde concentration with H_2_SO_4_ catalyst does not enhance MAX yield. Instead, halving it reduced the MAX yield from 25.4% to 17.5% (Figure S28). This suggests that product diffusion coefficients and local reactant concentrations in the confined environment jointly influence product selectivity. Other potential factors include the constrained flexibility of MAX due to confinement and the high local ionic strength within the zeolite pores.^[^ ^49^ ^]^ Further discussions on xylose acetalization selectivity and zeolite characterizations (NH_3_‐TPD, pyridine/acetonitrile‐d3‐FTIR, solid‐state NMR, and XRD) can be found in Supporting Information, Sections 7 and 5, respectively.

In summary, xylose acetalization with dodecanal by HY zeolite, particularly those with high SiO_2_/Al_2_O_3_ ratios, yielded higher MAX yields (i.e., a mixture of 3,5‐*O*‐acetal and 1,2‐*O*‐acetal), reaching around 74%. Moreover, the catalyst is well recyclable provided it is calcined between runs (at 550 °C for 6 h). This process likely burned off organic deposits, restoring the zeolite's microporosity and acidic active sites for reuse (see detailed discussion in Section S5.3), showing the potential for a scalable approach. The crystal phases, as well as their compositions, remained unchanged in the recycled catalysts, regardless of the number of recycles (Figure S19, Table S7). Nevertheless, the Lewis acid density slightly increased after repeated regeneration due to framework dealumination at high temperatures^[^ ^50^ ^]^ (Table S6). This change may affect the reaction selectivity by catalyzing unwanted side reactions. The long‐term stability of the zeolite should be carefully assessed in the future scale‐up studies.

Many major industrial surfactants feature a hydrophobic tail and a hydrophilic head that contains multiple carbon–oxygen bonds, notably by incorporation of ethoxylated units. These ethoxylated units are essential to tune the properties of the surfactant and notably to induce hardwater resistance to these molecules. Prominent examples include sodium laureth sulfate (SLES), which is a major component in many soaps and detergents,^[^ ^51^ ^]^ or polyoxyethylene alkyl ether carboxylic acids (AEC),^[^ ^52^ ^]^ which are similar to SLES but feature a carboxylic acid group instead of the sulfate. A major limitation of these surfactants is this fossil‐based ethoxylated unit, which has a non‐renewable source and poses biodegradation issues.^[^ ^53^ ^]^

**Figure 5:**
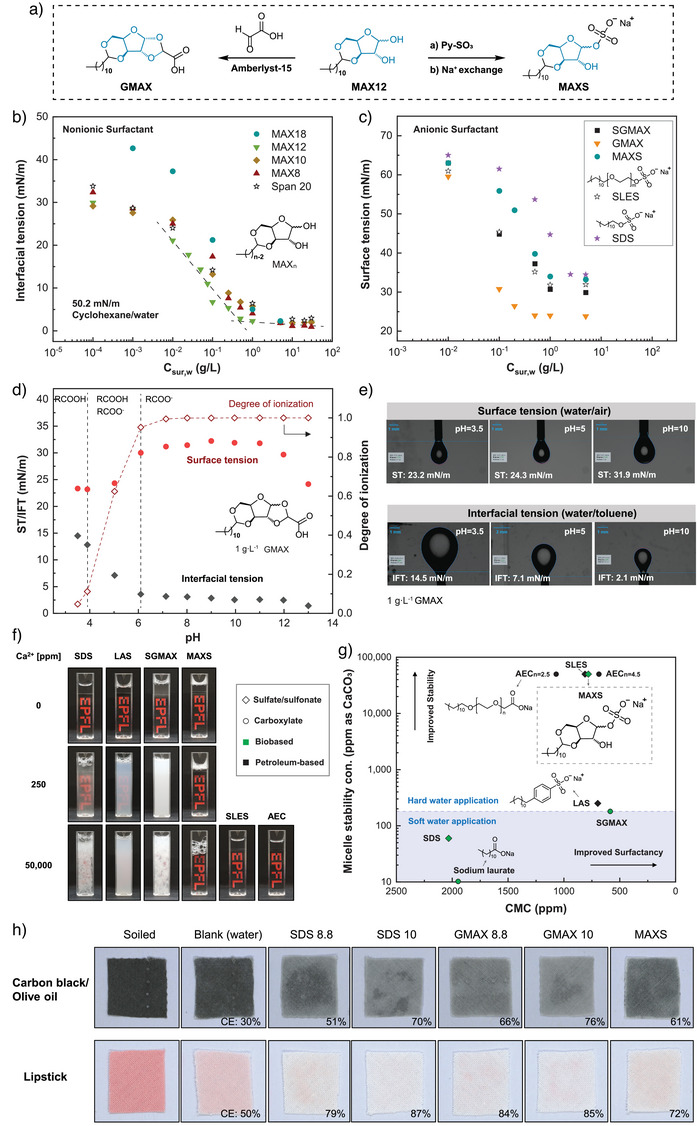
Amphiphilic properties of functionalized xylose MAX12, GMAX, and MAXS along with a comparison to commercial surfactants. a) Synthesis of GMAX and MAXS from MAX12. b) Interfacial tension (IFT) of the cyclohexane‐water interface at different concentrations of nonionic MAXn. c) Surface tension (ST) of water with different concentrations of GMAX, SGMAX (GMAX Na^+^ salt), MAXS, and commonly used commercial anionic surfactants. d) pH responsiveness of GMAX: ST of water and IFT of water/toluene with 1 g L^−1^ GMAX at different pH values. e) Measurement of ST and IFT of GMAX under different pH using pendant drop tensiometry. f) Hard water performance of MAXS, GMAX, and commercial surfactants: observation of surfactant solutions (at 1.5 times the Critical Micelle Concentration) through a cuvette at different water hardness levels (0 ppm, 250, and 50 000 ppm as CaCO_3_). g) Scatter plot of Critical Micelle Concentration (CMC) versus Micelle Stability concentration for MAXS, GMAX, and other commercial surfactants (details of individual experiments are provided in Supporting Information, Section 8.4; numerical values are provided in Table S4). h) Cleaning capacity of GMAX (suffixed with the pH, 8.8, 10) and MAXS (pH = 8.5) compared with SDS (pH = 8.8, 10) for removing different stains (standard stained cotton fabrics E‐101, CS116) using an ultrasonic cleaner. The cleaning efficiency (CE) calculation can be found in Section S8.8.

Selective sugar acetalization can allow us to incorporate a hydrophobic tail while maintaining xylose as a tunable hydrophilic head, thus mimicking the structure of these ethoxylated surfactants with a natural structure. In our previous work, we had developed xylose‐based surfactant mixtures through partial defunctionalizing DAX, a main product from aldehyde‐assisted biomass fractionation.^[^ ^54^ ^]^ However, this required a two‐step functionalization process and led to a mixture of compounds, preventing careful control of the resulting structures. Our development of size‐selective monoacetalization allows us to produce a single amphiphilic building block in one step. This new class of surfactants is denoted as MAXn, which hereafter represents only the 3,5‐*O*‐acetals of the two monoacetals of xylofuranose for simplicity (Figure 5a). These MAXn surfactants can significantly reduce the interfacial tension between oil and water. Among the variants with tail lengths of 8, 10, 12, and 18 carbons, MAX12 stands out with the lowest Critical Micelle Concentration (CMC) recorded at 410 ppm, achieving a plateau interfacial tension of below 5 mN m^−1^ in cyclohexane/water systems (Figure 5b). The emulsion stability of MAX12 was measured and compared to Span20, another carbohydrate‐based surfactant, and a blank, using optical microscopy over the course of 30 days (Figure S44). Water‐in‐oil (w/o) emulsions were generated by vortexing two phases: one comprising 2 mL of cyclohexane with 1 mg mL^−1^ surfactant and the other comprising 1 mL of water containing 1 mg mL^−1^ of Alcian blue. While the emulsion without surfactant quickly coalesced after emulsification (Figure S45), those stabilized by MAX12 remained stable for up to day 30 without any visible coalescence, as observed by optical microscopy. In contrast, emulsions prepared with Span20 exhibited larger droplet sizes initially, followed by an almost complete phase separation after day 7. This highlights MAX12's superior emulsion stabilizing capabilities compared to Span20. We also investigated the environmental stability of MAXn, specifically focusing on aqueous decomposition and biodegradation (refer to Section S8.9). Accelerated aqueous decomposition experiments revealed that MAX12 underwent rapid hydrolysis to xylose in neutral boiling water within 2 days. Additionally, MAX12 is readily biodegradable according to the OECD 301F method by monitoring biochemical oxygen demand.^[^ ^55^ ^]^

A key feature of the partially acetalized MAXn is that it is very simple to further functionalize. We extended its application to the synthesis of two types of anionic surfactants based on MAX12: a carboxylic acid‐based surfactant and a sulfate‐based surfactant (which notably serves as mimics of AEC and SLES structures) (Figure 5a,c). The former involved introducing carboxylic acid through acetalization with glyoxylic acid, denoted as GMAX (1,2‐*O*‐carboxylidene‐3,5‐*O*‐dodecylidene‐xylose), while the latter was synthesized by sulfating the free C1 hydroxyl group, denoted as MAXS (sodium 3,5‐*O*‐dodecylidene‐xylose‐1‐sulfate).

GMAX contains a weakly ionic, pH‐responsive carboxylic acid termination with a p*K*_a_ of 4.8, as determined through an acid‐base titration experiment (Figure S38). GMAX appears more acidic compared to lauric acid (p*K*_a_ = 5.3) because its carboxylic acid is situated adjacent to an acetal, an electron‐withdrawing group. The higher acidity, coupled with the presence of a sugar core, largely enhances the water solubility, which leads to better hard water resistance compared to sodium laurate, which rapidly precipitates in the presence of calcium ions (Figure S42). The sugar core likely acts as an analogue to the ethoxylated chain in AEC surfactant structures, enhancing water solubility and chelating metal cations to prevent precipitation and the loss of amphiphilic properties. Despite this, the hard water resistance is lower compared to AEC structures, likely due to the reduced efficiency of chelating metal cations caused by the rigidity of its tricyclic fused ring structure, as opposed to a flexible polyethylene oxide chain (PEO).^[^ ^56^, ^57^ ^]^ However, this issue can be overcome by sulfating a single hydroxyl group (see below).

Based on the degree of ionization of GMAX with respect to pH, as illustrated in Figure 5d, both ─COOH and ─COO^−^ coexist in the pH range of 4–6, where an increase in surface tension (ST) and a decline in interfacial tension (IFT) are observed as the pH level rises (Figure 5e). Below 4 and above 6, GMAX can be regarded as nonionic and completely ionic, respectively, with slight alterations in ST and IFT. This counterintuitive inverse correlation between ST/IFT with pH has been previously documented.^[^ ^52^, ^58^ ^]^ A detailed discussion on this phenomenon can be found in Section S8.2. Because of its pH‐responsive characteristics, GMAX could be a versatile surfactant with wide‐ranging applications. For instance, in industries such as cosmetics, food, pharmaceuticals, and delivery systems, operations are often facilitated through the use of responsive emulsions, which are activated by external stimuli.^[^ ^59^ ^]^ These emulsions undergo a shift between water‐in‐oil (w/o) and oil‐in‐water (o/w) states triggered by a pH change. Similarly, the system prepared with GMAX (pH ∼ 3.2) affords w/o emulsion, while SGMAX (sodium carboxylate, pH ∼7.6) affords o/w emulsion (Figures S47 and S49). In addition, surfactants are also employed to aid emulsion polymerization of latexes, which are commonly used in paper and paint coating, as well as the production of sealants and adhesives.^[^ ^60^ ^]^ To demonstrate SGMAX efficacy as a replacement for commercially utilized surfactants in these applications, o/w styrene emulsion polymerization was conducted using both SGMAX and sodium dodecyl sulfate (SDS), a very common commercial surfactant. In both cases, polymerization reached fairly high conversion (> 76%) within 5 h and high molecular weight (*M*_n _> 350 000 Da) while maintaining similar dispersity (*Đ* ∼ 2.0) (Table S18). The resulting latex exhibits remarkable stability, featuring a uniform particle diameter of around 68 nm (PdI = 0.031), with no discernible changes after 1 month (PdI = 0.028, Table S19). Furthermore, the preliminary ecotoxicity of GMAX has also been assessed and showed no general toxicity (ISO 11348–3: 2007), estrogenic activity (ISO 19040–1: 2018), or the inhibition of algal photosystem II (ISO 8692: 2004). Although minor algal growth inhibition was observed with GMAX, the induction concentration was over one order of magnitude higher than that of common commercial surfactants such as SDS^[^ ^61^, ^62^ ^]^ (detailed in Section S8.10). Beyond the aforementioned applications, GMAX also demonstrates outstanding performance in foaming (Figure S51), cleaning (Figure 5h), and emulsion stabilizing properties (Figures S47–S49). Moreover, its low Krafft point (<0 °C) enhances its versatility, enabling applications even in lower temperatures. Despite having far better hard water tolerance than sodium laurate, its performance is still affected by water hardness. In hard water conditions (> 180 ppm), the GMAX solution becomes turbid, and its capacity to lower surface tension is reduced.

We also developed a sulfate surfactant by sulfating C1–OH on xylose, which showed exceptional hard water resistance. MAXS exhibits a marked three‐order‐of‐magnitude increase in hard water stability compared with conventional surfactants like linear alkylbenzenesulfonates (LAS) and SDS, and was comparable with the commonly recognized hard water‐resistant surfactants, which include SLES and AEC (Figure 5g; see discussion in Section S8.4). Our testing concluded at 50,000 ppm of hardness, representing an extreme condition (Table S20). Under these conditions, the solutions of MAXS, SLES, and AEC remained clear, and no increase in surface tension was observed (Figures 5f and S43). The exceptional hard water tolerance of MAXS allows its application in extreme hard water conditions without the necessity to formulate chelating agents. We propose that the unique robustness of MAXS in hard water can be attributed to the presence of a free hydroxyl group within its sugar core, which resembles the function of ethoxylated moieties in SLES and AEC. These moieties act as chelation sites with metal cations and enhance their water solubility, thereby preventing precipitation under severe hard water conditions (for more discussions about hard water resistance, see Section S8.4). Besides, MAXS also demonstrated good performance in foaming (Figure S51), cleaning (Figure 5h), emulsion stabilizing properties (Figure S50), and low Kraft point (<0 °C). Overall, this performance could allow the direct replacement of SLES with a fully renewable alternative in a huge number of commercial formulations. These results also demonstrate the use of an unmodified carbohydrate structure as a replacement for a petroleum‐derived ethylene oxide chain.

## Conclusion

We introduce a straightforward and effective approach for selectively acetalizing sugars and polyols with yields between 70% and 95%. Unlike conventional approaches, this size‐selective zeolite catalysis is highly selective, atom‐efficient, and easily recyclable, opening new avenues in bulk chemical synthesis such as bio‐based amphiphiles. The resulting monoacetal nonionic surfactant MAX12 and its derivatives (anionic surfactants GMAX and MAXS) offer renewable alternatives to petroleum‐based counterparts while achieving high biomass utilization efficiency, and—as tested for MAX12—biodegradability in wastewater. The replacement of a fossil‐based polyethylene oxide chain with a largely unmodified natural sugar structure could be applied to other bulk chemicals to improve their sustainability. We anticipate that the straightforward monoacetalization of sugars and polyols can be expanded far beyond the applications showcased here and can facilitate the development of other bio‐based products, potentially even aiding the manufacture of therapeutics that rely on selective protection of polyols and carbohydrates. While traditional microporous zeolites may restrict access to bulkier substrates such as disaccharides, other porous materials with tunable pore structures, such as metal–organic frameworks (MOFs), may offer complementary confinement environments and represent an interesting direction for future study.

## Supporting Information

The supporting information file includes chemicals and materials, analytical methods, synthesis and characterization of acetal products, catalyst characterization, substrate and zeolite dimensions and selectivity correlations, xylose acetalization reactions, and performance of xylose monoacetal surfactants. Original electronic notebook and experimental measurements can be found in Zenodo repository: https://doi.org/10.5281/zenodo.14937056

## Conflict of Interests

J.S.L. is part owner of Bloom Biorenewables Ltd., a start‐up company that is commercializing the aldehyde functionalization chemistry of biomass‐derived molecules. S.S., Z.J.L., T.N., and J.S.L. are inventors on an international patent (PCT/EP2023/081790) on the methods of synthesizing at least partially acetal‐protected sugars via heterogeneous catalysis. The remaining authors declare no conflict of interest.

## Supporting information



Supporting Information

## Data Availability

The data that support the findings of this study are openly available in [Zenodo] at (https://10.5281/zenodo.14937056), reference number (14937056).
