# Supplementary material for: Size‐Selective Functionalization of Sugars and Polyols Using Zeolites for Renewable Surfactant Production
Source: Angew Chem Int Ed Engl. 2025 Aug 15;64(39):e202511282. doi: 10.1002/anie.202511282 (PMC12455472; doi:10.1002/anie.202511282)
Supplement: Supplementary file 1 — Supporting Information [file ANIE-64-e202511282-s001.pdf]

Supporting Information

Size-selective Functionalization of Sugars and Polyols using Zeolites for Renewable Surfactant Production

*Songlan Sun[a §], Zezhong John Li[a §], Yu-Cheng Lin[a], Manon Rolland[a, b], Tom Nelis[a], Seongmin Jin[a], Shasha Zheng[a], Benjamin Nicolas Raffy[a], Wen Hua Bi[c], Esther Amstad[d], Jeremy S. Luterbacher[a]**

[a]*École Polytechnique Fédérale de Lausanne, Laboratory of Sustainable and Catalytic Processing, Station 6, 1015 Lausanne, Switzerland*

[b]*École Polytechnique Fédérale de Lausanne, Laboratoire des Polymères, Station12, 1015 Lausanne, Switzerland*

[c]*École Polytechnique Fédérale de Lausanne, Crystal Growth and Characterization Platform, Station 3, 1015 Lausanne, Switzerland*

[d]*École Polytechnique Fédérale de Lausanne, Soft Materials Laboratory, Station12, 1015 Lausanne, Switzerland*

[§]*Authors contributed equally to this work.*

**Corresponding author: jeremy.luterbacher@epfl.ch*

Table of Contents

1. Introductory figure 2

2. Chemical and materials 3

3. Analytical methods 6

4. Synthesis and characterization of acetal products 14

5. Catalyst characterization 28

6. Substrate and zeolite dimensions and selectivity correlations 45

7. Xylose acetalization reaction 52

8. Performance of xylose monoacetal surfactants (MAXn, GMAX, MAXS) 66

1. Introductory figure

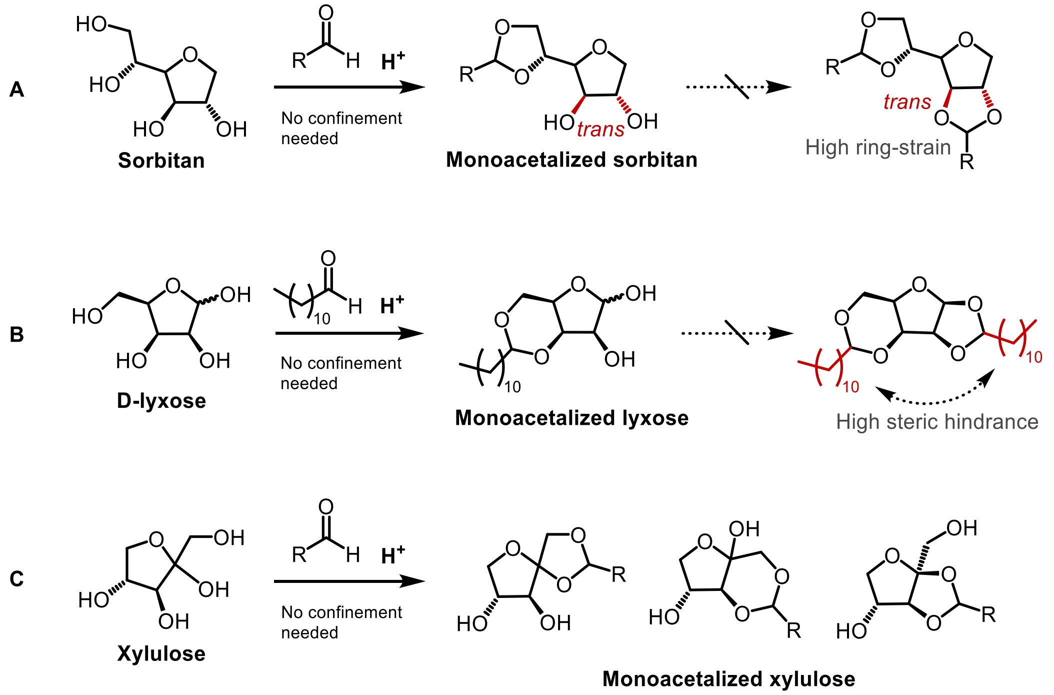

Figure S1. Acetalization of polyhydroxylated compounds forming only one low ring-strain cyclic acetal. (A) Monoacetalized sorbitan[1]: the formation of the second cyclic acetal would lead to a high ring-strain trans configuration[2]. (B) Monoacetalized lyxose (SI Figure S35): the formation of a "boat-like" tricyclic fused ring with bulky aldehyde would lead to high steric hindrance. (C) Monoacetalized xylulose (Figure 4c): the selectivity towards monoacetalization can be attributed to the high ring-strain *trans* configuration or large atomic distance between the free diols.

2. Chemical and materials

All commercial chemicals were of analytical grade and were used without further purification.

- 1,4-Dioxane ≥99.5%, Carl Roth AG
- Tetrahydrofuran (ACS Reagent Grade, Stabilized with BHT), Honeywell.
- Cyclohexane 99%, Fluorochem
- Cyclopentyl methyl ether (CPME) 99.9+%, stabilized, Chemie Brunschwig AG
- 2-Methyltetrahydrofuran (2-MeTHF) 99%, Carl Roth GmbH and Co. KG
- γ-Valerolactone (GVL) ≥99%, Sigma-Aldrich
- Dichloromethane (DCM) ≥99%, Chemie Brunschwig AG
- N-methyl-N-trimethylsilyl-trifluoroacetamide 99% (MSTFA), Fluorochem.
- Span® 20 (Fatty acid composition: Lauric acid (C12:0) ≥ 44%; balance primarily myristic (C14:0), palmitic (C16:0) and linolenic (C18:3) acids), Sigma-Aldrich
- Span® 80, TCI
- Tween® 20, Chemie Brunschwig AG
- ECOSURF SA-4, Sigma-Aldrich
- Sodium dodecyl sulfate 99%, Chemie Brunschwig AG
- Sodium dodecylbenzenesulfonate, technical grade, Sigma-Aldrich
- Sodium laureth sulfate, Chemie Brunschwig AG
- 1,2,4,5-Tetrachloro-3-nitrobenzene (standard for quantitative NMR), Sigma-Aldrich.
- Paraformaldehyde (granulated, extra pure), Carl Roth AG
- Propionaldehyde 97%, ABCR
- Pentanaldehyde 97%, IVALUA
- Trimethylacetaldehyde
- Octanaldehyde 99%, Sigma-Aldrich
- Decanaldehyde 95%, Acros Organics
- Dodecanaldehyde 95%, ABCR
- Octadecanaldehyde 95%, Chemie Brunschwig AG
- (*E*)-2-dodecenal, Sigma-Aldrich
- (Z)-4-dodecenal 94%, Sigma-Aldrich
- Glutaraldehyde, 50% in Water, Chemie Brunschwig AG
- Benzaldehyde ≥99.5%, Carl Roth AG
- Glyoxylic Acid Monohydrate 97%, Fluorochem

- Acetone
- Meso erythritol 99%, Chemie Brunschwig AG
- Pentaerythritol 98%, Chemie Brunschwig AG
- D-Threitol, Sigma-Aldrich
- Xylitol, Chemie Brunschwig AG
- D-(+)-xylose 99%, Sigma-Aldrich
- D-(+)-Glucose (BioUltra, anhydrous, ≥99.5%, sum of enantiomers, HPLC), Sigma-Aldrich
- L-arabinose 98%, IVALUA
- Xylulose 95%, Chemie Brunschwig AG
- Di-trimethylolpropane, Sigma-Aldrich
- Pyridine 99.5%, Acros Organics.
- Sulfur trioxide pyridine complex ($SO_3 \cdot Py$), Chemie Brunschwig AG
- Dimethylsulfoxide-d_6 (DMSO-d_6), Cambridge Isotope Laboratories.
- Sodium bicarbonate ($NaHCO_3$, 99%), Roth.
- 4,4'-azobis (4-cyanopentanoic acid) (ACVA), Sigma-Aldrich
- Styrene, Sigma-Aldrich
- Sudan black B, Sigma-Aldrich
- Column chromatography was performed with silica gel (P60 40-63 μm, 60 Å, Irregular Silica Gels), Silicycle
- Standard stained cotton fabrics, Center for Testmaterials (CFT)
- Polyoxyethylene alkyl ether carboxylic acids (AKYPO® RLM25 and AKYPO® RLM45 CA), Kao chemicals

The following commercial zeolites were used:

- H-MCM-22 ($Si/Al_2 = 7$) from China Catalyst Holding Co., Ltd.
- H-ZSM 22 ($Si/Al_2 = 65$-80) and H-ZSM 11 ($Si/Al_2 = 50$) from ACS Materials
- H-BETA ($Si/Al_2 = 30$; 150): from Clariant
- NH_4^+ ZSM-12 ($Si/Al_2 = 100$): from Clariant
- Templated (pyrrolidine) ZSM-23 ($Si/Al_2 = 100$): from Clariant

Obtaining zeolite ZSM-23 in the H^+ form:

Templated (pyrrolidine) ZSM-23 ($Si/Al_2 = 100$) from Clariant was calcined at 550 °C for 6 h in static air to remove organic matter. The calcined form was then ion-exchanged three times with a NH_4NO_3

solution (0.5 M) at 80 °C for 14 h. Finally, the proton form of the zeolite was obtained by performing another calcination step at 550 °C, 6 h in static air.

3. Analytical methods

<u>3.1 NMR</u>

All NMR spectra (^{1}H, ^{13}C, HSQC) were acquired using a Bruker Avance III 400 MHz spectrometer using the standard pulse sequences from Bruker. The purity of synthesized compounds was determined by quantitative ^{1}H NMR using 1,2,4,5-Tetrachloro-3-nitrobenzene as the internal standard.

<u>3.2 GC-MS</u>

Gas chromatography-mass spectrometry spectra were obtained using an Agilent 7890B series GC equipped with a HP5-MS capillary column and an Agilent 5977A series Mass Spectroscopy detector. Silylation derivatization was applied by adding 100 μL N-Methyl-N-(trimethylsilyl)-trifluoroacetamide (MSTFA) and 100 μL pyridine and kept under room temperature (r.t.) for 30min before detection. The GC-MS method was performed as follows: The injection temperature was 300 °C. 1 μL of the sample was injected with an autosampler in split mode (split ratio: 25:1). The column was initially kept at 40 °C for 3 min, then was heated at a rate of 30 °C·min^{-1} to 100 °C, followed by a heating rate of 40 °C·min^{-1} to 300 °C that was held for 5 min.

<u>3.3 GC-FID</u>

Quantitative analysis of monoacetals and diacetals were performed with an Agilent 7890B series GC equipped with an HP5 column and a FID. Silylation derivatization was applied by adding 100 μL N-Methyl-N-(trimethylsilyl)-trifluoroacetamide (MSTFA) and 100 μL pyridine and kept under r.t. for 30min before detection. The temperature ramping program was the same as that of the GC-MS. Quantification was done either by using external calibration standards (synthsized using the methods outlined in SI section 4), or correlation equations based on effective carbon number theory or the number of functional groups. The calibration curves were prepared while correcting for the purity of each isolated compound as determined by quantitative ^{1}H NMR.

<u>GC response factor calibration</u>

Sugar-based monoacetals and diacetals. GC response factors (RF) were estimated based on a regression dependent on the number of silylation groups (N_{Si}), the number of carbon atoms additional to the sugar carbons ($N_{C\text{-}a}$), and the number of carbon atoms in the sugar core ($N_{C\text{-}s}$) (Eq. S1). This regression was built using measured values of a selection of isolated products (*i.e.*, DAX1, DAX3, DAX8, DAX10, DAX12, MAX8, MAX10, and MAX12. See Figure S2 for calibration curves) to build an overall calibration for our product mixture (Table S1). The MAXn were silylated before calibration to protect vulnerable hydroxyl groups in the GC column.

The RF can be calculated:

Equation S1: Response factor (L/mol) = 27744 $N_{C\text{-}a}$ + 44360 N_{Si} + 13170 $N_{C\text{-}s}$

Table S1. Calculated RFs versus the measured RFs of sugar-based monoacetals and diacetals. Calculated RFs in italics were not used for calculating concentration but are given to illustrate the accuracy of the approach.

	$N_{C\text{-}s}$	$N_{C\text{-}a}$	N_{Si}	Calculated RF (L/mol)	Measured RF (L/mol)
DAX1	5	2	0	*121339*	109333
DAX3	5	6	0	*232316*	232170
DAX5	5	10	0	343294	–
DAX-Bn	5	12	0	398783	–
DAX8	5	16	0	*509760*	546634
DAX10	5	20	0	*620738*	632011
DAX12	5	24	0	*731716*	731659
DAX18	5	36	0	1064649	–
DAX12,2=	5	24	0	731716	–
DAX12,4=	5	24	0	731716	–
MAX1	5	1	2	182314	–
MAX3	5	3	2	237803	–
MAX5	5	5	2	293292	–
MAX-Bn	5	6	2	321036	–
MAX8	5	8	2	*376525*	364979
MAX10	5	10	2	*432014*	418439
MAX12	5	12	2	*487502*	487142
MAX18	5	18	2	653969	–
MAX12,2=	5	12	2	487502	–
MAX12,4=	5	12	2	487502	–
DAG12 (glucose)	6	24	1	835376	–
MAG12 (glucose)	6	12	3	575668	–
DAA12 (arabinose)	5	24	0	731716	–
MAA12 (arabinose)	5	12	2	487502	–

Polyol-based monoacetals and diacetals. The concentrations of acetalized polyols are measured using a different method. Specifically, we used the effective carbon number (ECN) method[3] using MAX12 and DAX12 with known RFs as references (Eq. S2 and S3), and verified the method accuracy with MAE12. A resulting deviation of 2% validates the accuracy of the ECN method.

Equation S2: $\text{Response factor}_{polyol\ monoacetal}\ (\text{L/mol}) = \text{Response factor}_{MAX12} \times \frac{ECN_{polyol\ monoacetal}}{ECN_{MAX12}}$

Equation S3: $\text{Response factor}_{polyol\ diacetal}\ (\text{L/mol}) = \text{Response factor}_{DAX12} \times \frac{ECN_{polyol\ diacetal}}{ECN_{DAX12}}$

Table S2. Calculated RFs versus the measured RFs of polyol-based monoacetals and diacetals. The calculated RFs in italics were not used for calculating concentration but are given to illustrate the accuracy of the approach.

	ECN*	Calculated RF (L/mol)	Measured RF (L/mol)
MAX12	18.8	–	487142
DAX12	24	–	731659
MAE12 (erythritol)	18.8	*487142*	496868
MAPE12 (pentaerythritol)	19.8	513054	–
MAXylitol12	22.2	575242	–
DAE12 (erythritol)	24	731660	–
DAPE12 (pentaerythritol)	25	762146	–
DAXylitol12	27.4	835312	–

*ECN contribution of the atoms obtained from[4]: aliphatic and aromatic carbons are counted 1, oxygen-ether -1, –C–O–Si$(CH_3)_3$ are 3.4.

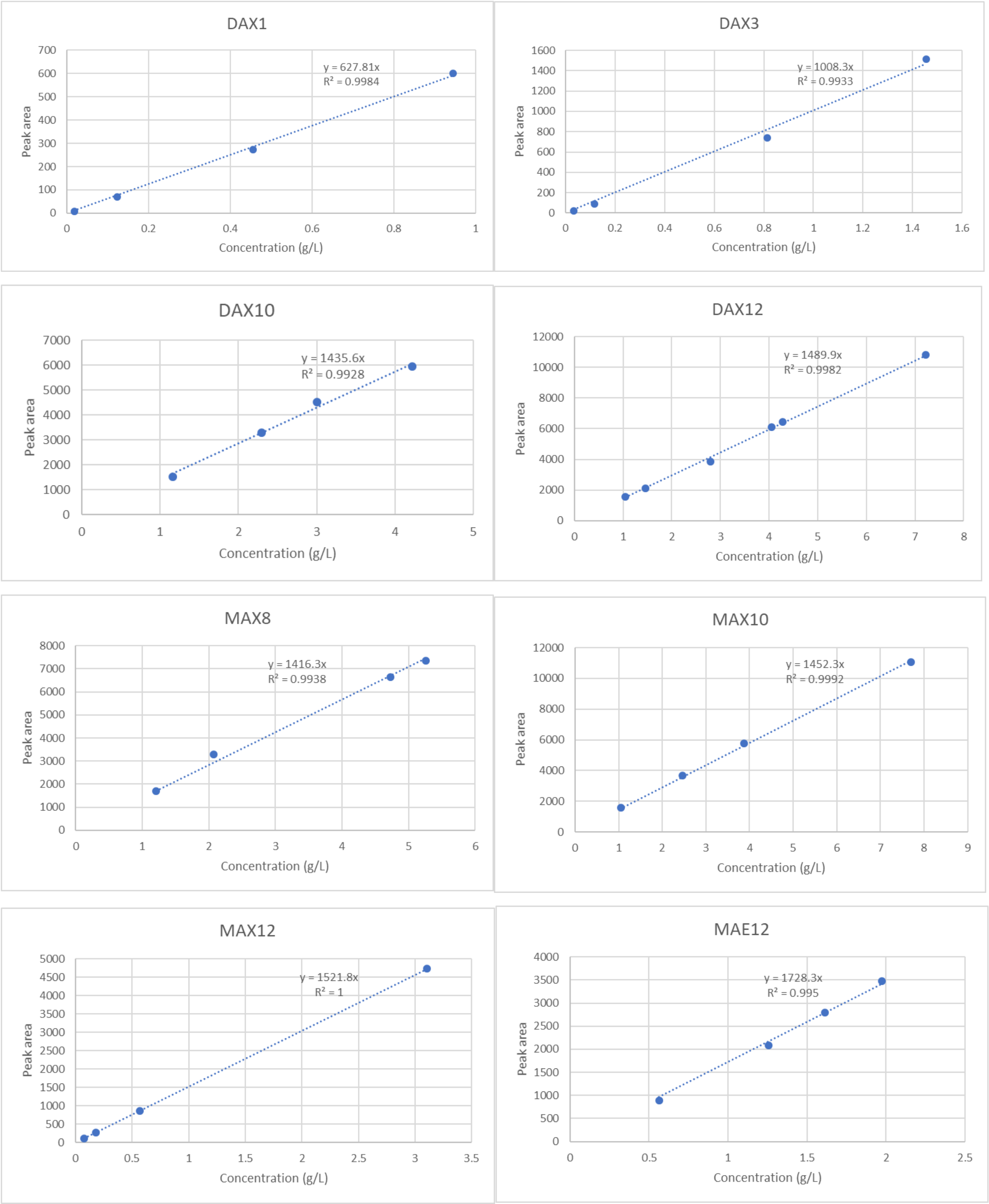

Figure S2. Calibration curves used for the quantification of sugar- and polyol-based mono- and di-acetals by GC-FID.

3.4 HPLC (C18 reverse phase chromatography)

Any unreacted sugars or polyols in the reaction mixture were quantified using an Agilent Infinity 1260 HPLC system equipped with a Pursuit XRs C18 column (150 x 10 mm, 5μm) using H_2O/ACN (90/10) as eluent (flow rate = 2 mL·min^{-1}, V_{inj} = 40 μL) at 25 °C with Refractive Index Detector (RID) (G1362A). Quantifications of individual components were performed using external calibration standards that were commercially available (Figure S3).

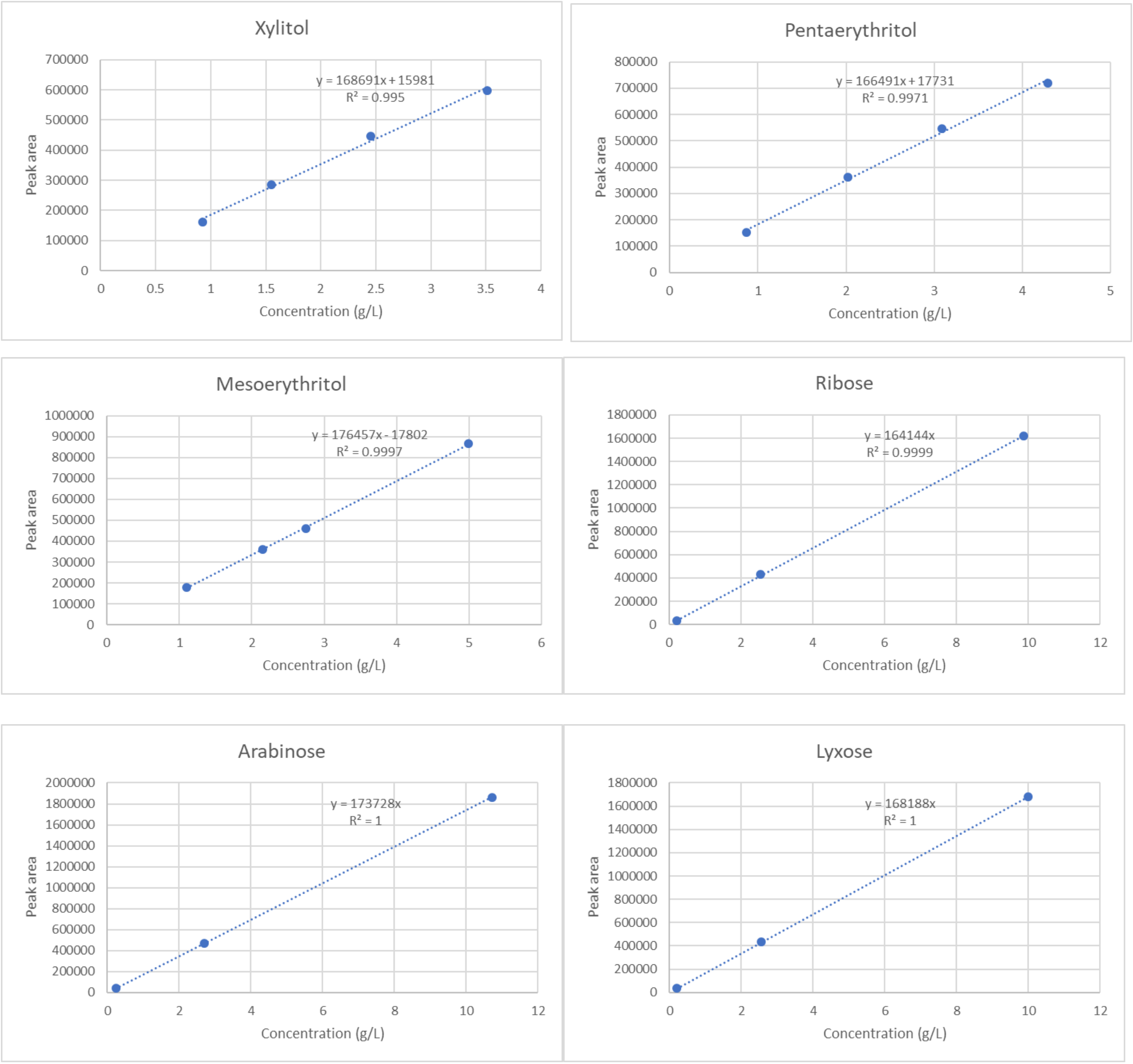

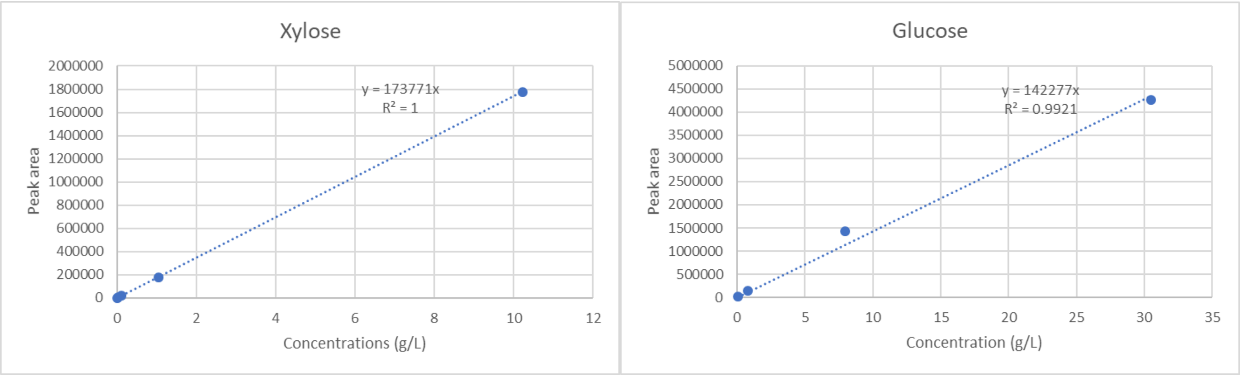

Figure S3. Calibration curves used for the quantification of unreacted sugars and polyols in the reaction mixture by HPLC (C18 reverse phase).

3.5 HPLC (pH 2 aqueous-phase chromatography)

Xylose produced from the accelerated aqueous decomposition experiment was quantified by an Agilent Infinity 1260 HPLC system equipped with an Aminex HPX-87H Column (300 mm x 7.8 mm, column temperature = 60 °C) using pH2 water as eluent (flow rate = 0.6 mL·min^{-1}, V_{inj} = 20 μL) with Refractive Index Detector (RID) (G1362A). The quantifications were done using external calibration standards prepared from commercial samples (as an illustration the calibration curve for xylose is shown below).

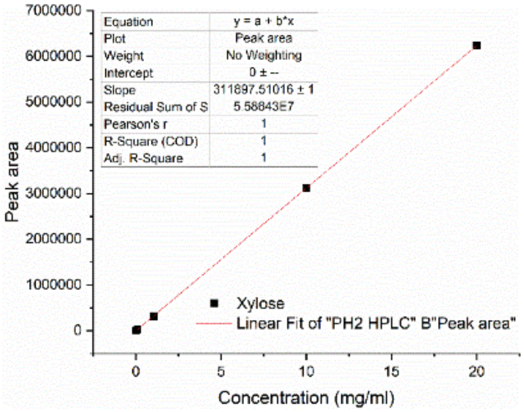

Figure S4. Calibration curve used for xylose quantification by HPLC (pH 2 aqueous phase).

3.6 Pendant drop test

Interfacial tension. We measured the water/oil interfacial tension using the pendant drop test. We prepared a series of different concentration surfactant organic solutions and load in a 1 mL syringe and inserted them onto a Kruss SDA 30 drop shape analyzer. In a typical test, we slowly injected the organic solution containing the surfactants into the water phase with a bent needle of 1 mm diameter. The drops were created and analyzed using the Kruss Advance software (v.1.6.2.0). The critical micelle concentration (CMC) was

obtained by checking the point where the plateau started to form in the interfacial tension-concentration graph.

Surface tension. We measured the surface tension of the water with surfactants using the pendant drop test. We prepared a series of different concentration surfactant aqueous solutions and load in a 1 mL syringe and inserted them onto a Kruss SDA 30 drop shape analyzer. In a typical test, we slowly made a pendant drop of the surfactant aqueous solution and calculated the surface tension of water by analyzing the shape of the pendant drop (Young-Laplace equation) using the Kruss Advance software (v.1.6.2.0). The critical micelle concentration (CMC) was obtained by checking the point where the plateau starts to form in the surface tension-concentration graph.

3.7 Gel Permeation Chromatography (GPC)

Gel permeation chromatography (GPC) analysis was performed on a SECcurity2 GPC system (PSS Polymer Standards Service GmbH, Germany) equipped with a SECcurity2 refractive index detector, a GRAM precolumn of 50 mm length, and three GRAM columns of 300 mm length. All columns had a diameter of 8 mm and a particle size of 20 μm. Sample analysis was performed in dimethylacetamide (DMAc) + 0.1 wt % LiBr at 70 °C at a flow rate of 1.0 mL·min^{-1}, using polystyrene (PS) standards.

3.8 Optical microscopic imaging of emulsions

Emulsions were observed using a Nikon Eclipse TS100 Inverted Microscope. Pictures were taken immediately after the emulsification and every seven days thereafter to observe the droplets' evolution and evaluate the emulsions' stability.

3.9 Dynamic light scattering (DLS)

The polystyrene latex was analyzed by DLS (Nano ZS from Malvern) to find the particle size distribution and zeta potential.

3.10 Determination of Hard Water Tolerance

The surfactants' hard water tolerance was assessed by measuring turbidity and surface tension (refer to Section S3.6: Pendant drop test) upon exposure to water of varying hardness. Standard hard water samples were prepared by dissolving target quantities of $CaCl_2$, and expressed in terms of equivalent hardness of $CaCO_3$. The turbidity created by the introduction of hard water was determined by a Turbidimeter with a testing range of 0-1000 NTU.

3.11 Determination of Krafft temperature

The Krafft point of the surfactants was determined by estimating the extent of counterion dissociation using a conductivity meter (Labor-Konduktometer 703, Knick) that was immersed in the surfactant solution. This meter was capable of measuring both conductivity and temperature. The measurement began at 0 °C and

was gradually heated. Conductivity was measured at every 1 °C increment until it reached a steady value. The Krafft point was identified as the temperature at which the conductivity vs. temperature graph displayed a significant change in slope. Krafft points below zero cannot be measured exactly and are just listed as < 0°C.

4. Synthesis and characterization of acetal products

4.1 General reaction conditions (small scale optimization)

Reactions with homogeneous mineral acid (H_2SO_4)

D-xylose or other polyols (1.67 mmol, 1.0 equiv.), the aldehyde (3.34 mmol, 2 equiv.) and sulfuric acid (0.1 mmol to make 0.02 M H_2SO_4) were added to dioxane (5 mL) in a 10 mL glass reactor. The reaction was conducted at 65 °C for 5 h in most cases. Then, the solution was neutralized with solid $NaHCO_3$ until no gas was produced. Solid $NaHCO_3$ was separated by centrifugation, and all liquor was collected in a 10 mL volumetric flask in preparation for quantitative analysis. The unreacted polyols were analyzed by reverse phase HPLC (SI section 3.4), and quantitative analysis of monoacetal and diacetal were performed using GC-FID after silylation (SI section 3.3).

Reactions with heterogeneous solid acid (Zeolites)

Sugars or other polyols (1.67 mmol, 1.0 equiv.), the aldehyde (3.34 mmol, 2 equiv.) and 0.25 g of the zeolite were added to dioxane (5 mL) in a 10 mL glass reactor. The reaction was conducted at 65 °C for 5 h unless otherwise mentioned. Then, the zeolite catalyst was separated by centrifugation, and the catalyst was washed with fresh dioxane for three times with sonication. All the organic liquor was collected in a 20 mL volumetric flask in preparation for quantitative analysis. The zeolite was then washed three times with MQ water while sonicating. The aqueous liquor was collected in a 5 mL volumetric flask in preparation for quantitative analysis. Both organic and aqueous liquors were analyzed by reverse phase HPLC to quantify the unreacted sugars or polyols (SI section 3.4), and the quantitative analysis of monoacetals and diacetals were performed using GC-FID after silylation of the organic liquor (SI section 3.3).

We set the aldehyde-to-polyol molar ratio at 2 to balance selectivity with yield. A lower ratio improves monoacetalization but reduces conversion due to equilibrium limitations and aldehyde side reactions that further lower its effective concentration. Additionally, maintaining this stoichiometric ratio ensures sufficient aldehyde supply for full conversion to diacetals, eliminating the effect of limiting reagents on the monoacetal selectivity. Therefore, the role of zeolite pore confinement can be assessed with less interference.

Determining the aldehyde partition coefficient in the HY80 zeolite

Various aldehydes were added to 1,4-dioxane to reach a concentration of ca. 0.1 M. In cases where the aldehyde source solution contained a substantial quantity of water (e.g., aqueous formaldehyde and glutaraldehyde solutions), the mixture was pre-dried before being put in contact with the zeolite. Then, 2

mL of the mixture was mixed with 0.1 g of HY80 zeolite and stirred at 25°C for 2h. Experiments were run in duplicate. The mixture was filtered with a 0.2 μm PTFE syringe filter. The concentration of the respective aldehyde before and after this test was analyzed by GC, $C_{initial}$ and C_b, respectively. The measurement was conducted in duplicate. Formaldehyde and valeraldehyde were analyzed by reverse phase HPLC due to peak overlapping on GC spectra. The concentration of aldehyde inside the zeolite pore (C_p) was calculated using the mass balance of the aldehyde, $C_p = \frac{C_{initial} \cdot V - C_b \cdot V}{V_{pore}}$.

Therefore, $C_p / C_b = \frac{C_{initial}/C_b \cdot V - V}{V_{pore}} = \frac{V}{V_{pore}} (C_{initial}/C_b - 1) = \frac{V}{V_{pore}} (A_{initial}/A_b - 1)$.

where $A_{initial}$ and A_b are the peak areas of the respective aldehyde before and after the test, V is the volume of the solution (V=2 mL, assuming constant solvent volume through the sorption test), and the V_{pore} is the pore volume of HY80 as measured by Ar adsorption. Since only relative quantities were used, we did not use concentration calibration curves for GC or HPLC analysis. This method estimates the bulk concentration of aldehyde absorbed inside the zeolite, irrespective of the interaction modes with the zeolite internal surface. Considering the highly porous nature of zeolites (Table S5), the interaction with the external surface is negligible, and therefore not accounted for in this estimation.

4.2 MAXn (monoalkylidene-xylose) preparation (≥ 5 g synthesis)

D-xylose (10 g, 66.6 mmol, 1.0 equiv.), the aldehyde (99.9 mmol, 1.5 equiv.) and HY zeolite (SiO_2:Al_2O_3 = 80:1, 5 g) were added to dioxane (200 mL) in a two-neck 500 mL round bottom flask fitted with a condenser. When (*E*)-2-dodecenal was used, molecular sieve needed to be added to remove water in order to shift the equilibrium to the product. The mixture was then heated to 80 °C for 1.5 h-2 h under nitrogen flow. The resulting solution was cooled down to room temperature (~ 25 °C), and zeolite catalyst was removed by filtration. The filtered zeolite was washed three times with fresh dioxane. All the liquor was then collected and concentrated on a rotary evaporator with a bath temperature of 45 °C under reduced pressure (80 mbar). Subsequently, a small amount of hexane was added to facilitate complete dissolution. The solution was then refrigerated to induce precipitation. MAX-a and MAX-b were precipitated due to their lower solubility in hexane, to afford white waxy solid. Purification can also be done through flash chromatography using EtOAc/hexane (15% - 75% gradient) as eluents.

GC-FID measurement showed 68.8% MAX12-a (3,5-*O*-dodecylidene-xylose) yield, 5.1% MAX12-b (1,2-*O*-dodecylidene-xylose) yield, and 3.9% DDX (didodecylidene-xylose) from D-xylose with dodecanal. The isolated products were a combination of MAX12-a and MAX12-b and represented a cumulative isolated yield of approximately 60%.

4.3 MAEn (monoalkylidene-erythritol) preparation (≥ 5 g synthesis)

Meso-erythritol (5 g, 40.5 mmoles, 1 eq.), dodecyl aldehyde (14.2 mL, 60.8 mmoles, 1.5 eq.) and 2.5 g ZSM5 (SiO_2/Al_2O_3=80/1) zeolite catalyst were added to dioxane (120 mL) in a two-neck 250 mL round bottom flask fitted with a condenser. The mixture was then heated to 80 °C for 1 h under nitrogen flow. The resulting solution was cooled down to room temperature (~ 25 °C), and the zeolite catalyst was removed by filtration. The filtered zeolite was washed three times with fresh dioxane. All the liquor was collected and concentrated on a rotary evaporator with a bath temperature of 45 °C under reduced pressure (80 mbar). After removing most of the solvent, the mixture became wax-like white solid. Then, the white solid was dispersed in hexane and stirred overnight. The product (mixture of 1,2-*O*-dodecylidene-mesoerythritol and 1,3-*O*-dodecylidene-mesoerythritol, ratio:1/1 based on ^{1}H NMR) was collected by filtration. The GC yield was 88.8%, the isolated yield was 83.5%, and the purity was 97.0%.

4.4 Synthesis of 1,2-*O*-carboxylidene-3,5-*O*-dodecylidene-xylose (GMAX) from 3,5-*O*-dodecylidene-xylose (MAX12)

In a single neck round bottom flask, 1 molar equivalent of MAX12 was mixed with 2 molar equivalent of glyoxylic acid monohydrate in dioxane (which was dried with anhydrous Na_2SO_4 or molecular sieves overnight). Amberlyst A15 was used as catalyst and molecular sieves were added to remove any water resulting from the reaction. The reaction was conducted at 80 °C for 4h. Then, the solution was filtered and concentrated on a rotary evaporator with a bath temperature of 45 °C under reduced pressure (80 mbar). Following this, the reaction mixture was purified using column chromatography using hexane-ethyl acetate with 1% acetic acid as the solvent to obtain pale yellow solid (purity: 96%).

Catalyst recycling. Despite the attempt to wash the catalyst after reaction, some products and reactants were suspected to be strongly adsorbed to Amberlyst surface due to the relatively low yield and mole balance. Catalyst recycling was conducted three times to evaluate this hypothesis. After the procedure above, Amberlyst was dried in vacuo at 45 °C over night and recycled to another reaction. The product yields are summaried in Figure S5. The increase in GMAX yield and the improved mole balance upon catalyst recycling suggested that products from the previous cycle likely strongly adsorbed to the catalyst surface and could not be washed off easily. The conversion over the catalyst remained stable over all 3 reaction cycles. Product adsorption is not expected to be a challenge in a contirnous flow reactor as the steady state would be established over time after the catalyst surface adsorption reaches equilibrium.

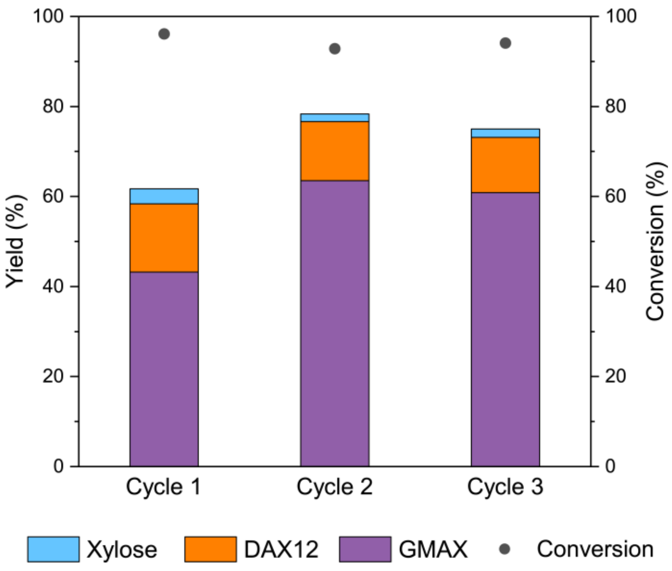

Figure S5. Product yields and MAX12 conversions upon caralyst recycling in GMAX synthesis.

4.5 Synthesis of Sodium 3,5-*O*-dodecylidene-xylose-1-sulfate (MAXS) from 3,5-*O*-dodecylidene-xylose (MAX12)

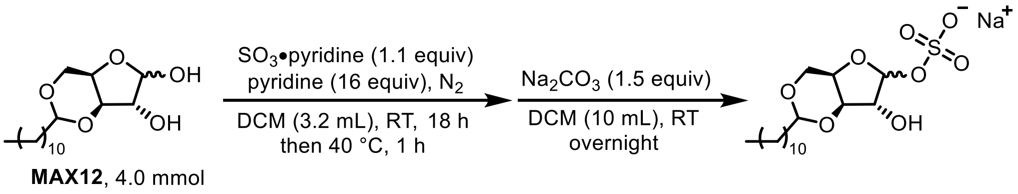

MAX12 (4.0 mmol, 1264 mg) was added to a 50 mL Schlenk flask along with a magnetic stirring bar and SO_3·Py (1.1 equiv, 700 mg). The flask was put under vacuum for 30 mins and charged with N_2. While under N_2, we added dichloromethane (3.2 mL) and pyridine (16 equiv., 5.2 mL) to the flask. The reaction was stirred at room temperature for 18 h and then at 40 °C for 1 h. After cooling down to room temperature, the insoluble solid was filtered off and the solution was evaporated at 55 °C. Then we added Na_2CO_3 (1.5 equiv) and dichloromethane (10 mL) to the crude mixture to exchange the cation. The reaction solution was stirred at room temperature for 18 h. After removal of the solvent through evaporation, flash column chromatography (C18 reverse-SiO_2, MeOH/H_2O) was applied to obtained the purified MAXS. The isolated yield was around 25% (stoichiometric reaction, yield loss occurred during flash chromatography due to the challenge of separating pyridinium). In industry, sulfating can be performed using sulfur trioxide which leads to a much faster reaction rate, high atom economy (100%), no side product, and easy workup.

Table S3. Comparison of the synthesized nonionic surfactants and select commercial surfactants

	Entry	Surfactant	HLB	CMC_{IFT} (ppm)	γ_{IFT} (mN m^{-1})
Monoacetalized Xylose	1	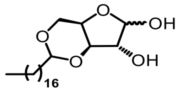 **3,5-*O*-octadecanalidene-xylose (MAX18)**	6.2	1491	2.3
	2	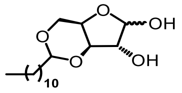 **3,5-*O*-dodecylidene-xylose (MAX12)**	9.0	410	1.9
	3	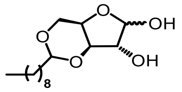 **3,5-*O*-decylidene-xylose (MAX10)**	10.0	1500	2.0
	4	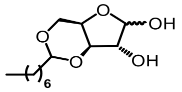 **3,5-*O*-octylidene-xylose (MAX8)**	10.9	2100	0.9
	5	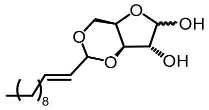 **3,5-O-(E)-dodec-2-en-1-ylidene-xylose (MAX12:1(2))**	9.0	447	2.2
	6	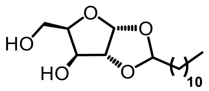 **1,2-*O*-dodecylidene-xylose**	9.0	860	2.6
	7	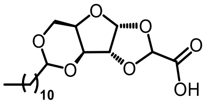 **1,2-*O*-carboxylidene-3,5-*O*-dodecylidene-xylose (GMAX)**	9.9	282	<0.5
Commercial Surfactants	8	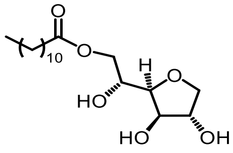 **Sorbitan monolaurate (Span® 20)**	8.6	2630	3.0

9	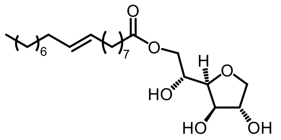 **Sorbitan oleate (Span® 80)**	4.3	660	4.8
10	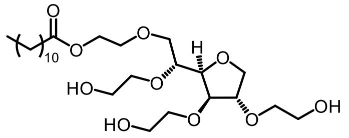 **Polyoxyethylene sorbitan laurate (Tween®20)**	16.7	630	0.6
11	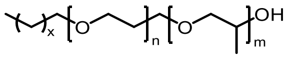 **ECOSURF SA-4**	7.5	2780	<0.5

Table S4. Comparison of the synthesized anionic surfactants and select commercial surfactants

	Entry	Surfactant	HLB	CMC $_{ST}$ (ppm)	γ_{ST} (mN m^{-1})	Krafft Point (°C)
**Anionic Surfactants based on monoacetals**	12	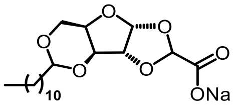 **Sodium 1,2-*O*-carboxylate-3,5-*O*-dodecylidene-xylose (SGMAX)**	20.4	585	30	<0
	13	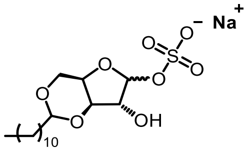 **Sodium 3,5-*O*-dodecylidene-xylose-1-sulfate (MAXS)**	45.8	781	33	<0
	14	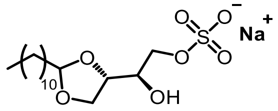 **Sodium 3,4-*O*-dodecylidene-erythritol sulfate (MAES)**	42.6	1072	28	26

**Commercial Surfactants**	15	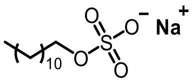<br>**Sodium dodecyl sulfate (SDS)**	40	2032	35	15
	16	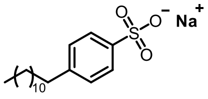<br>**Linear alkylbenzenesulfonates (LAS)**	38.34	697	30	20
	17	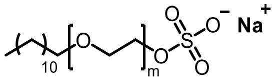<br>**Sodium laureth sulfate (SLES)**	40+0.35m	811	32	<0
	18	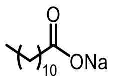<br>**Sodium laurate**	20.9	1950	20	25[5]
	19	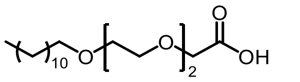<br>**Polyoxyethylene alkyl ether carboxylic acids (AKYPO® RLM25)**	5.4	204	26	-
	20	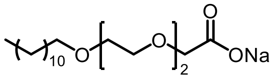<br>**Polyoxyethylene alkyl ether carboxylate**	22.4	1069	33	-
	21	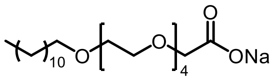<br>**Polyoxyethylene alkyl ether carboxylate (AKYPO® RLM45 CA)**	23.1	687	36	<0

4.6 Characterization data of MAXn/MAEn

NMR

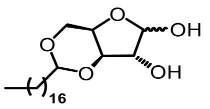

3,5-*O*-octadecylidene-xylose (MAX18)

^{1}H NMR (400 MHz, $CDCl_3$) δ 5.65 (d, J = 3.7 Hz, 0.6H), 5.11 (s, 0.4H), 4.25 – 4.16 (m, 2H), 4.16 – 4.05 (m, 2H), 4.00 – 3.78 (m, 1H), 1.65-1.53 (m, 2H), 1.42 – 1.01 (m, 30H), 0.81 (t, *J* = 6.7 Hz, 3H).

^{13}C NMR (101 MHz, $CDCl_3$) δ 104.32, 100.52, 80.46, 73.94, 71.85, 67.40, 34.94, 34.83, 29.69, 29.64, 29.55, 29.47, 23.69, 22.80, 14.24.

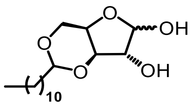

3,5-*O*-dodecylidene-xylose (MAX12)

^{1}H NMR (400 MHz, $CDCl_3$) δ 5.69 (d, *J* = 3.8 Hz, 0.6H), 5.17 (s, 0.4H), 4.51-4.40 (m, 1H), 4.25 – 4.16 (m, 2H), 4.16 – 4.05 (m, 2H), 4.00 – 3.78 (m, 1H), 1.65-1.53 (m, 2H), 1.41 – 1.21 (m, 18H), 0.87 (t, *J* = 6.7 Hz, 3H).

^{13}C NMR (101 MHz, $CDCl_3$) δ 104.32, 100.52, 80.46, 73.94, 71.85, 67.40, 34.94, 34.83, 29.69, 29.64, 29.55, 29.47, 23.69, 22.80, 14.24.

α/β anomer: 60/40

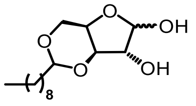

3,5-*O*-decylidene-xylose (MAX10)

^{1}H NMR (400 MHz, $CDCl_3$) δ 5.61 (d, *J* = 3.7 Hz, 0.6H), 5.13 (s, 0.4H), 4.46-4.30 (m, 1H), 4.30 – 4.12 (m, 1H), 4.15 – 4.09 (m, 1H), 4.09 – 4.02 (m, 1H), 4.04 – 3.82 (m, 1H), 3.82-3.73 (m, 1H), 1.59 – 1.48 (m, 2H), 1.36 – 1.10 (m, 14H), 0.82 (t, *J* = 6.7 Hz, 3H).

^{13}C NMR (101 MHz, $CDCl_3$) δ 104.16, 100.42, 80.32, 74.93, 71.58, 67.20, 34.47, 33.96, 29.51, 29.42, 29.39, 29.31, 23.29, 22.80, 14.09.

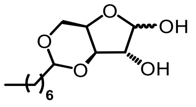

3,5-*O*-octylidene-xylose (MAX8)

^{1}H NMR (400 MHz, $CDCl_3$) δ 5.59 (s, 0.66H), 5.12 (s, 0.34H), 4.44-4.34 (m, 1H), 4.26-4.18 (m, 1H), 4.16-4.06 (m, 3H), 4.07 – 3.99 (m, 1H), 1.72 – 1.43 (m, 2H), 1.40-0.95 (m, 10H), 0.82 (t, *J* = 6.1 Hz, 3H).

^{13}C NMR (101 MHz, $CDCl_3$) δ 104.11, 100.37, 80.27, 73.81, 71.52, 67.14, 34.65, 31.71, 29.31, 29.28, 29.12, 29.10, 23.58, 22.57, 14.02.

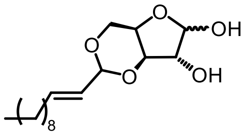

3,5-*O*-(*E*)-dodec-2-en-1-ylidene-xylose (MAX12:1(2))

^{1}H NMR (400 MHz, $CDCl_3$) δ 7.12 – 6.69 (m, 1H), 6.20 – 5.78 (m, 1H), 5.66 (dd, *J* = 8.5, 3.8 Hz, 1H), 5.38 (ddt, *J* = 5.7, 2.0, 1.1Hz, 1H), 4.82 (dd, *J* = 4.2, 3.3 Hz, 10H), 4.64 – 3.62 (m, 5H), 2.36 – 1.84 (m, 2H), 1.20 (d, *J* = 12.3 Hz, 14H), 0.81 (t, *J* = 6.6 Hz, 3H).

^{13}C NMR (101 MHz, $CDCl_3$) δ 159.34, 136.95, 136.85, 132.93, 125.80, 125.43, 124.62, 104.29, 99.30, 99.14, 97.74, 92.71, 80.52, 79.35, 79.12, 77.34, 77.23, 77.03, 76.71, 75.30, 73.60, 71.56, 66.85, 61.76, 32.76, 32.06, 32.04, 31.89, 31.85, 29.51, 29.50, 29.46, 29.44, 29.35, 29.31, 29.28, 29.26, 29.22, 29.18, 29.14, 28.57, 27.84, 22.67, 22.66, 14.11.

The characterization of MAXn, including HSQC-NMR, is shown in Figure S6, and the GC-MS results are presented in Figures S7–S9.

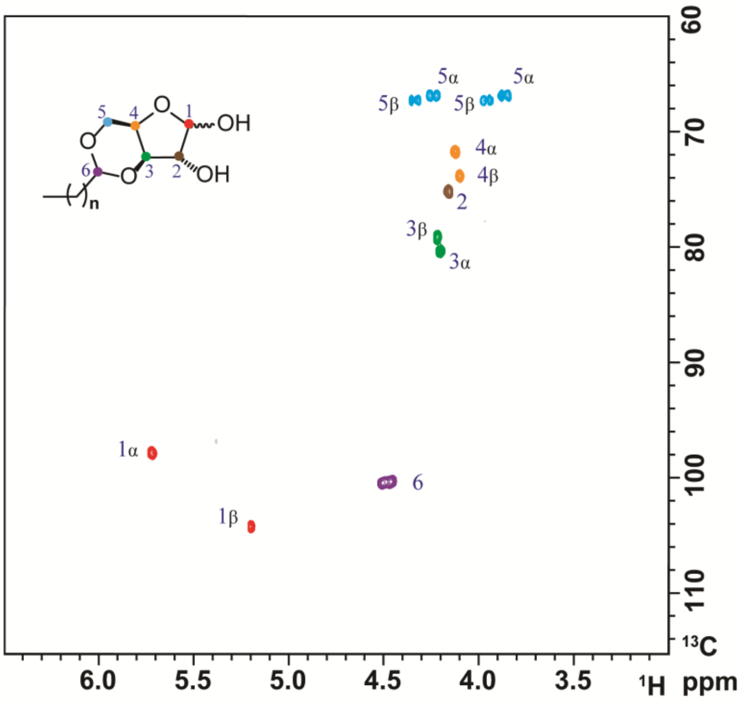

Figure S6. Characterization of 3,5-*O*-dodecylidene-xylose (MAX12) via HSQC-NMR.

GC-MS

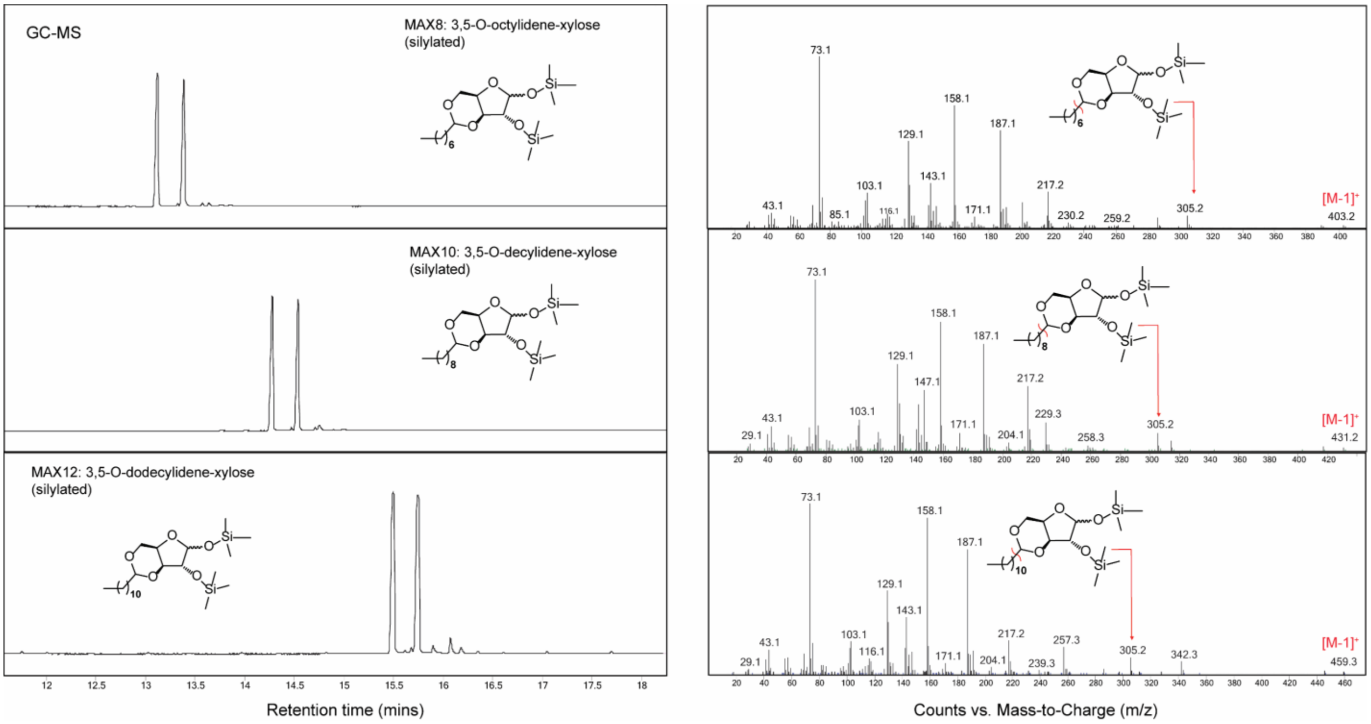

Figure S7. GC-MS chromatogram of purified MAXn (n=8,10,12) isomers and mass spectrum of MAXn (n=8,10,12) isomers.

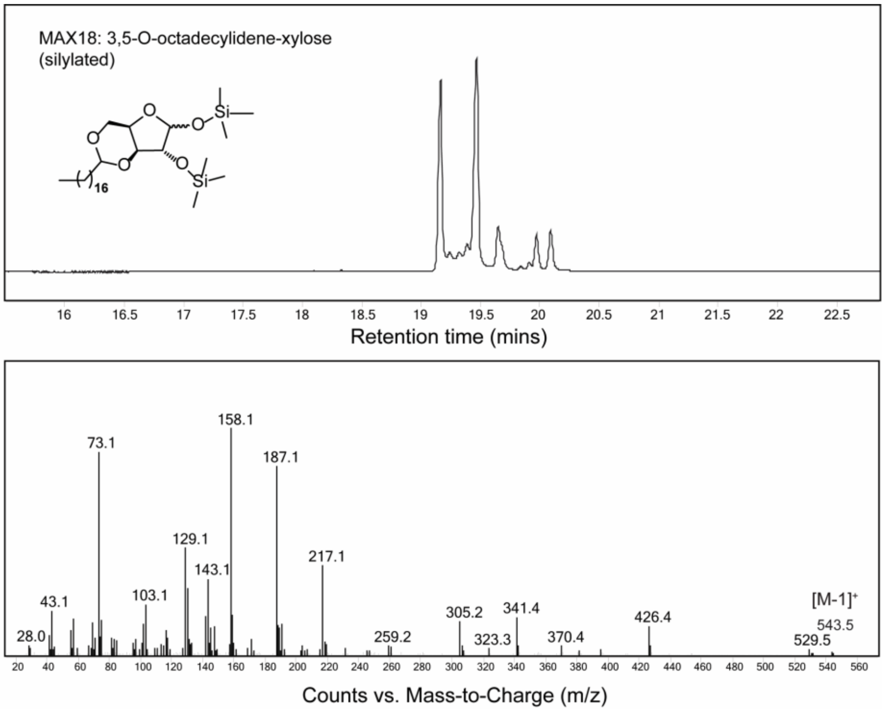

Figure S8. GC-MS chromatogram of purified MAX18 isomers and mass spectrum of MAX18 isomers.

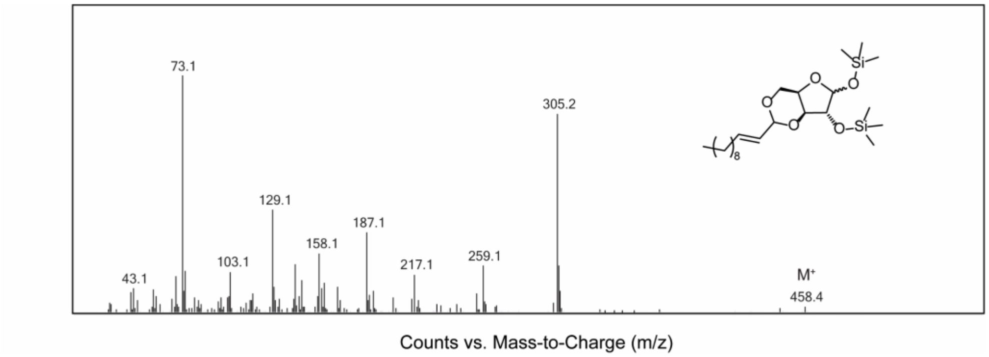

Figure S9. Mass spectrum of MAX12:1(2).

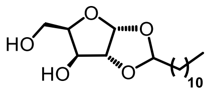

1,2-*O*-dodecylidene-xylose

^{1}H NMR (400 MHz, $CDCl_3$) δ 5.96 (d, *J* = 3.7 Hz, 1H), 5.17 (t, *J* = 4.7 Hz, 0.52H), 4.92 (t, *J* = 4.8 Hz, 0.48H), 4.49 (d, *J* = 3.6 Hz, 1H), 4.42 – 4.31 (m, 2H), 4.16 – 3.93 (m, 4H), 1.16 – 1.42 (m, 18H), 0.86 (t, *J* = 6.7 Hz, 6H).

^{13}C NMR (101 MHz, $CDCl_3$) δ 106.91, 105.62, 104.65, 104.55, 86.43, 86.22, 81.47, 78.79, 77.09, 76.96, 61.33, 61.20, 34.72, 34.09, 32.03, 29.76, 29.75, 29.73, 29.63, 29.62, 29.56, 29.54, 29.48, 29.46, 23.85, 23.65, 22.80, 14.24.

HRMS (nanochip-ESI/LTQ-Orbitrap) m/z: $[M + H]^+$ Calcd for $C_{17}H_{33}O_5^+$ 317.2323; Found 317.2318.

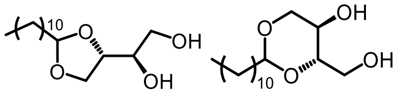

1,2-*O*-dodecylidene-mesoerythritol, 1,3-*O*-dodecylidene-mesoerythritol (MAE12) (ratio:1/1) mixture

^{1}H NMR (400 MHz, $CDCl_3$) δ 4.98 (t, *J* = 4.9 Hz, 0.5H), 4.85 (t, *J* = 4.8 Hz, 0.5H), 4.16 (dd, *J* = 8.5, 6.2 Hz, 0.5H), 4.10 – 3.95 (m, 1.5H), 3.95 – 3.75 (m, 3H), 3.75 – 3.60 (m, 1.5H), 1.70-1.56 (m, 2H), 1.43 – 1.19 (m, 18H), 0.94 – 0.80 (m, 3H).

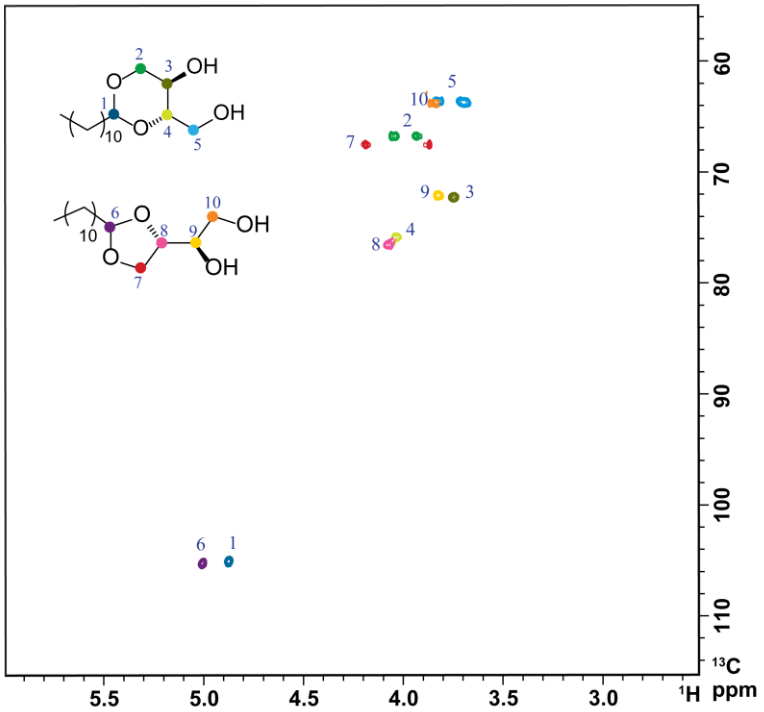

Figure S10. Characterization of the MAE12 mixture via HSQC-NMR.

4.7 Characterization data of GMAX

NMR

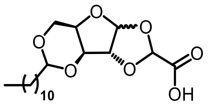

1,2-*O*-carboxylidene-3,5-*O*-dodecylidene-xylose (GMAX)

^{1}H NMR (400 MHz, $CDCl_3$) δ 6.09 (d, *J* = 3.8 Hz, 1H), 5.31 (s, 1H), 4.58 (dd, *J* = 59.7, 3.8 Hz, 1H), 4.37 (dt, *J* = 5.2 Hz, 1H), 4.22 (dd, *J* = 23.7, 2.8 Hz, 1H), 4.17 (dd, 1H), 3.99 (dt, 1H), 3.83 (ddd, *J* = 13.4, 8.3, 2.1 Hz, 1H), 1.49 (ddq, *J* = 9.7, 5.6, 2.6 Hz, 2H), 1.28 – 1.10 (m, 18H), 0.79 (t, *J* = 6.7 Hz, 3H).

^{13}C NMR (101 MHz, $CDCl_3$) δ 171.46, 170.66, 106.52, 106.44, 100.42, 100.34, 100.04, 99.69, 85.83, 84.58, 77.82, 73.33, 73.31, 67.05, 65.85, 34.60, 34.57, 31.91, 29.63, 29.62, 29.53, 29.48, 29.35, 23.75, 22.69, 20.60, 14.12.

The characterization of GMAX, including NMR analysis, is shown in Figure S11, and the GC-MS results are presented in Figure S12.

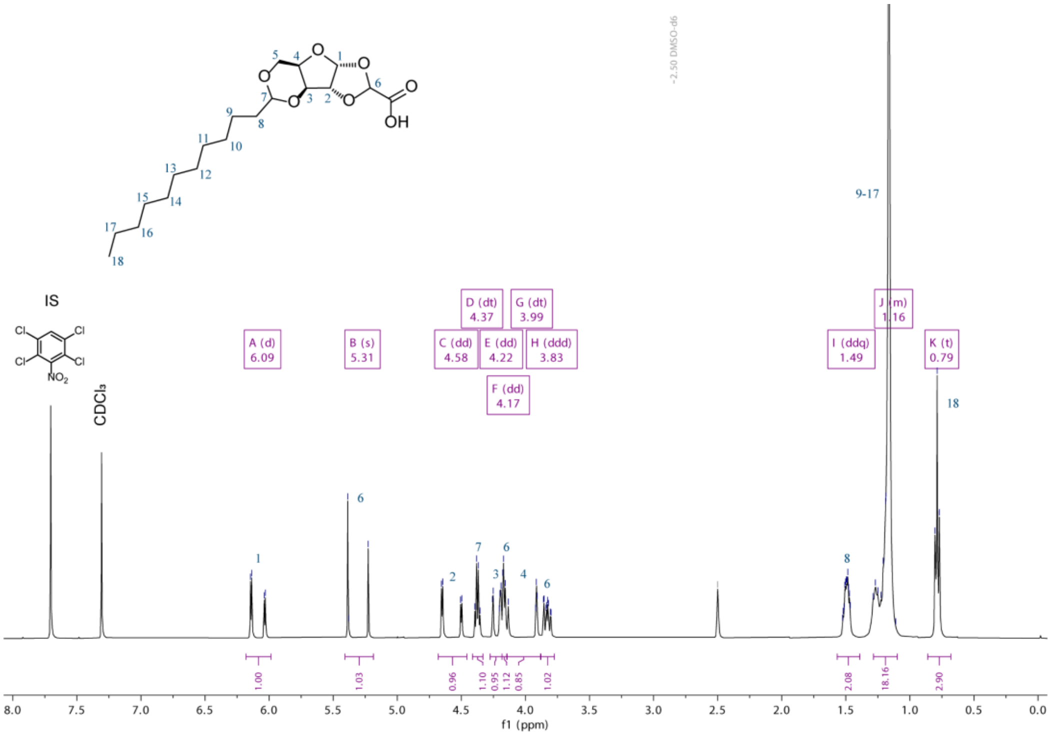

Figure S11. NMR spectrum of 1,2-O-carboxylidene-3,5-O-dodecylidene-xylose (GMAX).

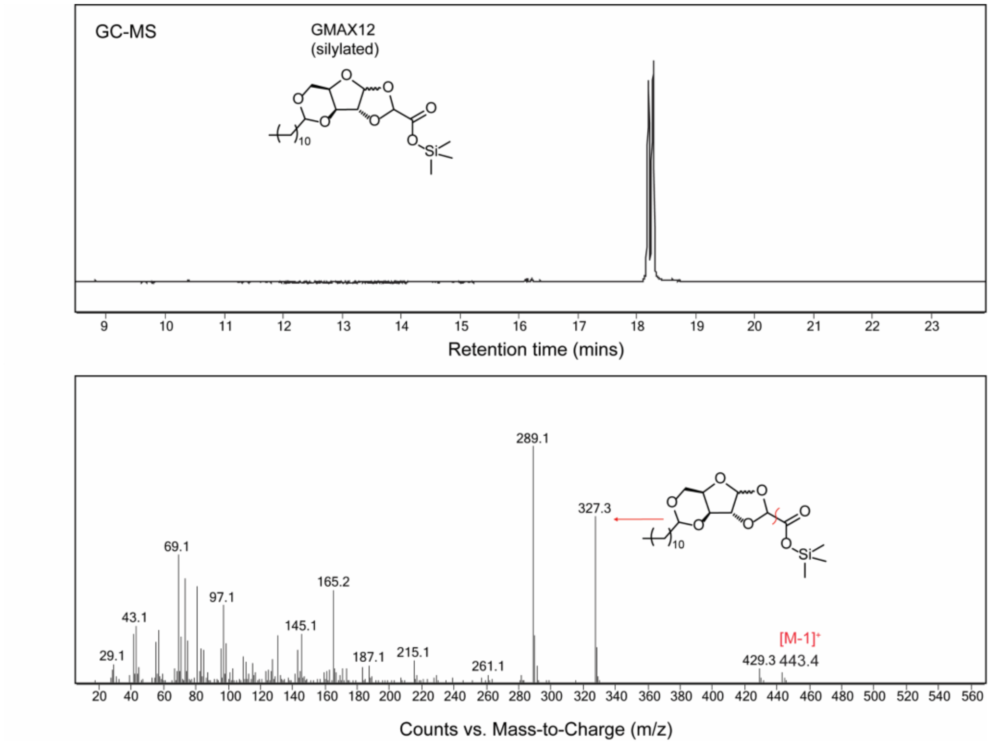

Figure S12. GC-MS chromatogram of purified GMAX12 isomers and mass spectrum of GMAX12 isomers.

4.8 Characterization data of MAXS and MAES

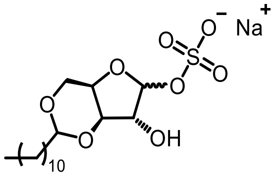

Sodium 3,5-*O*-dodecylidene-xylose-1-sulfate (MAXS)

^{1}H NMR (400 MHz, $CDCl_3$) δ 5.67 (d, *J* = 3.8 Hz, 0.4H), 5.39 (s, 0.6H), 4.68-4.49 (m, 3H), 4.23 – 3.88 (m, 3H), 1.65-1.53 (m, 2H), 1.41 – 1.21 (m, 18H), 0.87 (t, *J* = 6.7 Hz, 3H).

HRMS (ESI/QTOF) m/z: $[M]^-$ Calcd for $C_{17}H_{31}O_8S^-$ 395.1745; Found 395.1752.

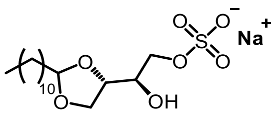

Sodium 3,4-*O*-dodecylidene-erythritol sulfate (MAES)

^{1}H NMR (800 MHz, d-Dioxane) δ 4.84 (t, *J* = 4.9 Hz, 0.5H), 4.73 (t, *J* = 4.9 Hz, 0.5H), 4.01 (dd, *J* = 8.6, 6.5 Hz, 0.5H), 3.97 – 3.88 (m, 1.5H), 3.86 – 3.75 (m, 2H), 3.71 (dd, *J* = 8.7, 6.9 Hz, 0.5H), 3.62 (m, 0.5H), 1.58 – 1.38 (m, 2H), 1.27 – 1.08 (m, 18H), 0.75 (t, *J* = 7.0 Hz, 3H).

HRMS (ESI/QTOF) m/z: $[M]^+$ Calcd for $C_{16}H_{31}O_7S^-$ 367.1796; Found 367.1806.

5. Catalyst characterization

5.1 Characterization methods

5.1.1 NH_3-TPD (Ammonia Temperature-Programmed Desorption)

The concentration of acid sites was calculated from NH_3 temperature programmed desorption (NH_3-TPD) performed on a Micromeritics Autochem 2920 II instrument. Typically, the sample (ca. 100 mg) was loaded to a U-shaped cell and dried for 1 h under He flow (50 mL·min^{-1}) at 450 °C (5 °C·min^{-1}). The procedure was modified for the never regenerated 6-time recycled HY80 which was degassed at 105°C for 6 h to avoid organic contaminants being pyrolyzed before NH_3 sorption. After cooling down to 150 °C, the flow was switched to a 1:99 (volumetric ratio) NH_3:He mixture for 1 h to saturate the sample with NH_3. The sample was then flushed with He (50 mL·min^{-1}) at 150 °C for 1 h to remove physisorbed NH_3 and the temperature was ramped to 800 °C at a rate of 10 °C·min^{-1}. During this process, ammonia desorption was monitored using a calibrated thermal conductivity detector as well as MKS Cirrus II mass spectrometer calibrated to mass 16. The physisorbed NH_3 was assumed to be removed during the flushing step as the mass signals attributed to NH_3 remained very low at the beginning of the temperature ramp and up to 200 °C (see Figure S14).

The calibration of NH_3 using the mass spectrometer was conducted by increasing the concentration of NH_3 stepwise and integrating the area below the profile of m/z=16 during the static flow. The obtained area was correlated to the total amount of NH_3 flowing through the chamber (Figure S13).

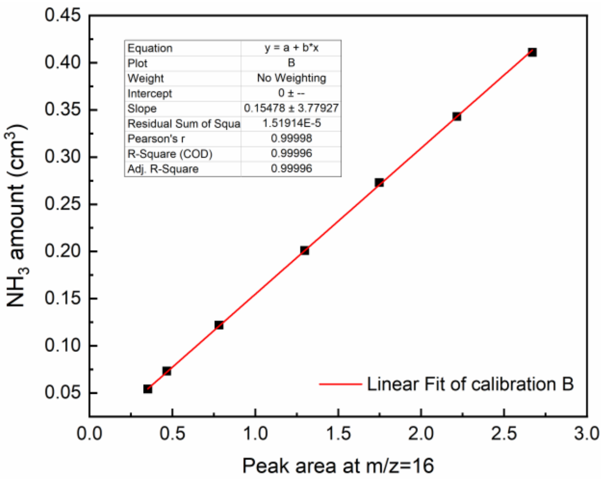

Figure S13. Calibration curve used for NH_3 quantification during the NH_3-TPD analysis with the MKS Cirrus II mass spectrometer.

5.1.2 FTIR

Diffuse reflectance infrared Fourier transform spectroscopy (DRIFTS) spectra were recorded using a high temperature Harrick DRIFTS cell on a Perkin Elmer Frontier spectrometer equipped with a mercury cadmium telluride detector. Spectra were typically collected with 128 scans at a resolution of 4 cm^{-1}. The undiluted and freshly calcined zeolite was manually loaded in sample holder of the DRIFTS cell. The sample surface was smoothed by compressing it with a polished stainless-steel surface to enhance IR reflection. The sample was first degassed under 0.01 mbar at 450 °C for 1.5 h. An exception was the never regenerated 6-time recycled HY80 which was degassed at 105 °C over night. The choice of the probe molecule depended on the zeolite pore size. Pyridine was used as the probe molecule except for the noted exceptions. Specifically, due to the small channel dimensions of ZSM-22 and ZSM-23, deuterated acetonitrile (CD_3CN) was used instead of pyridine because pyridine would have likely experienced significant diffusion limitation when entering the zeolite pores[6].

For CD_3CN-FTIR, helium was flushed at 30 mL·min^{-1} once the temperature was reduced to 25 °C after degassing. A pre-adsorption spectrum was recorded. Helium was then bubbled through a bath of anhydrous CD_3CN at room temperature to saturate the samples with the probe molecule for 75 min at 25 °C. The gas atmosphere was then switched to He at 30 mL·min^{-1} for 1 h to remove physisorbed CD_3CN at 25 °C, after which a post-adsorption spectrum was recorded.

For pyridine-FTIR, helium was flushed at 30 mL·min^{-1} once the temperature was reduced to 150°C after degassing. A pre-adsorption spectrum was recorded. Helium was then bubbled through a bath of anhydrous pyridine at room temperature to saturate the samples with the probe molecule for 75 min at 150°C. The gas atmosphere was then switched to He at 30 mL·min^{-1} for 1 h to remove physisorbed pyridine at the same temperature, after which a post-adsorption spectrum was recorded.

5.1.3 Solid-state NMR (ssNMR)

MAS ssNMR spectra were recorded on a 900 MHz Bruker Avance spectrometer (21.14 T) with an Avance Neo console equipped with a CP-MAS wide bore HCND quadruple channel 3.2 mm probe using a 3.2 mm zirconia rotor with Vespel caps at a spinning speed of 20 kHz. ^{1}H spectra were recorded using a Hahn-echo sequence of $\pi/2$-τ- π -aq. where the echo delay τ corresponded to 1 rotor period of 0.05 ms and $\pi/2$ was 3.25 µs. ^{27}Al spectra were recorded using a Hahn-echo sequence with τ = 0.05 ms and $\pi/2$ = 3 µs without {^{1}H} decoupling. Decoupling was attempted but showed negligible improvement in spectrum quality. The recycle delay was selected to be 1.3 T1. ^{27}Al chemical shifts were relative to 1.1 *m* $Al(NO_3)_3$ in water and ^{1}H in reference to the secondary reference of adamantane at 1.82 ppm.

5.1.4 Thermogravimetric Analysis (TGA)

Carbonaceous deposits on catalysts during the xylose acetalization reaction was characterized with Thermogravimetric Analysis (TGA) on a Perkin Elmer TGA 8000 instrument. Typically, a sample weighing approximately 3 mg was placed in a ceramic crucible and subjected to a 2 h drying step at 105 °C to eliminate any residual solvents. Subsequently, the temperature was gradually increased to 800 °C at a rate of 5 $°C \cdot min^{-1}$, with the experiments conducted under both air and nitrogen atmospheres.

5.1.5 Ar physisorption analysis

A Micromeritics 3Flex Surface Characterization Analyzer was used for Ar physisorption tests. All the samples were dried at 250°C under vacuum ($<10^{-3}$ mbar) overnight. One exception was that the never-regenerated 6-time recycled HY80 catalyst was dried at 105°C for 3 days to avoid the degradation of organic contaminants at 250°C. Notably, Ar was used instead of N_2 as the sorbate because i) N_2 interacts strongly with micropores at low P/P_0, rendering the measurement hard to control, and ii) the monoatomic Ar molecule has an unambiguous cross-sectional dimensions compared to cylindrical N_2 molecules whose cross-sectional dimensions depend on the orientation. The adsorption measurement was conducted at 77K. While IUPAC recommends argon physisorption at 87 K for micropore analysis due to improved accuracy and kinetics[7], liquid argon was not available at our facility. To address this limitation, we used a validated method tailored for Ar adsorption at 77 K. Specifically, we applied the fitting model developed by Tarazona et al.[8][9], which accounts for argon adsorption behavior in zeolites with cylindrical pores. To improve equilibration accuracy at 77 K, small argon dosing steps (as low as 3 cm^3/g STP) and a tight equilibration threshold (0.01%) were used. Importantly, the same experimental conditions and analysis protocols were applied across all samples, ensuring consistency in relative comparisons of surface area and porosity.

The Brunauer-Emmett-Teller (BET) surface area and Barrett-Joyner-Halenda (BJH) mesoporous volume were calculated using the equipment software (3Flex Version 5.01) without further parameter modification. Pore size distributions were obtained using nonlocal density functional theory (NLDFT) as implemented in the software, based on the 77 K Ar adsorption model developed by Tarazona et al.[8][9]. The average pore size of each catalyst was calculated as the first moment of the pore size distribution using numerical integration.

5.1.6 Powder X-Ray Diffraction (XRD)

The regular XRD measurements for zeolite samples were performed on a Panalytical Empyrean X-Ray polycrystalline diffractometer in Bragg-Brentano geometry, equipped with long-focused sealed X-Ray tube of Cu ($\lambda K\alpha$ = 1.5418 Å), and a PIXcel 1D X-Ray detector. All patterns were collected in continuous mode between 5 and 60 degrees (2θ), with the step-size of 0.02626 degrees. The Match! Version 4 software was

used to process XRD patterns. To calculate the crystallite size, LaB_6 was first used as the standard to calibrate the instrumental peak broadening. Zeolite patterns were then fitted, and the average crystallite sizes were calculated against the instrumental standard using the Scherrer equation.

5.2 Zeolite physicochemical properties: surface Area, porosity, and acidity

Table S5. Textural and acidic properties of the various catalysts

**Catalyst**	**Total S_{BET} (m^2/g)**	**Micropore area (m^2/g)**	**Total Pore volume (cm^3/g) @0.95 P/P_0**	**Micropore volume (cm^3/g)**	**Pore diameter (Å)**	**Crystallite size (Å)**	**LAS density (µmol/g)**	**BAS density (µmol/g)**
NaY5.2	748.8 ± 11.8	687.9	0.35	0.31	7.01	654.2	150.8	0.0
HY5.2	512.0 ± 7.2	457.9	0.26	0.20	7.49	490.6	193.3	125.1
HY80	784.3 ± 15.7	558.3	0.58	0.29	7.78	458.7	80.2	33.9
Hβ30	608.4 ± 7.1	411.3	0.32	0.18	6.85	145.0	185.0	290.1
Hβ150	692.6 ± 8.2	456.7	0.42	0.19	6.92	225.3	100.8	52.5
ZSM22	404.4 ± 4.2	275.1	0.26	0.12	5.65	244.3	488.5	326.4
ZSM23	233.6 ± 2.2	132.2	0.13	0.05	5.86	319.8	194.9	188.6
ZSM5	448.9 ± 6.0	365.8	0.24	0.16	6.03	441.4	132.0	270.1
ZSM11	296.9 ± 3.4	202.1	0.19	0.09	6.03	226.8	97.1	525.6
ZSM12	327.5 ± 4.2	249.5	0.19	0.11	6.30	210.5	175.8	134.3
MCM22	400.2 ± 4.2	267.3	0.28	0.11	6.54	170.2	169.9	339.8
HY80 6 times calcined	694.4 ± 9.1	424.2	0.38	0.18	7.68	-	85.0	34.1
HY80 6 times uncalcined	413.4 ± 2.9	226.8	0.23	0.09	6.76	-	64.5	0.0

Total acid density is calculated by integrating the signal area by NH_3-TPD, and the ratio of Brønsted and Lewis acid sites is provided by pyridine-FTIR spectroscopy.

BAS stands for Brønstad acid site, and LAS for Lewis acid site.

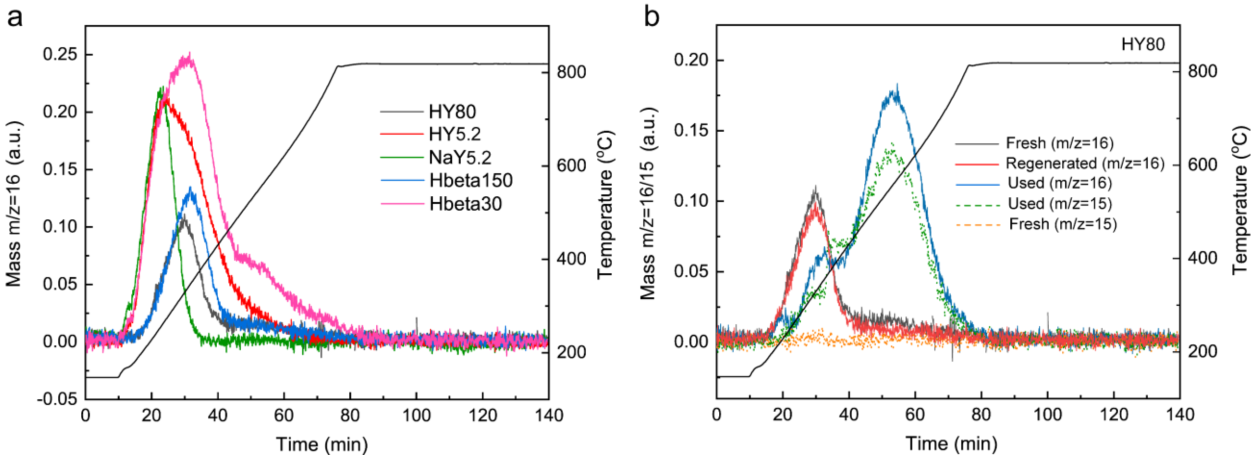

Figure S14. NH_3-Temperature Programmed Desorption (TPD)-MS profile of (a) different zeolites, (b) fresh, used, and regenerated HY80 zeolite with the signal of m/z=16, and 15.

When the never regenerated 6-time recycled HY80 sample was analyzed, the organic contaminants left in the sample were inevitably pyrolyzed during the temperature ramp, producing a large amount of methane which also contributed to the mass fragment m/z=16. This increased the MS signal of m/z=16, leading to an overestimation of NH_3 desorption. The pyrolysis may also generate CO, CO_2 gases. However, since we set the detection over a mass range of m/z 15 to 18 for NH_3 detection, we did not check for signals corresponding to CO or CO_2. To subtract the methane influence, we calibrated the methane mass fragment ratio of m/z=15 to m/z=16 at various concentrations (Figure S15). We assumed that methane was solely responsible for the signal of m/z=15 during the TPD and could then calculate the contribution of methane to the mass fragment ratio of m/z=16 and subtract it. In fact, ammonia produced over 30 times more of the mass fragment m/z=16 compared to m/z=15, which was measured during the NH_3 calibration, while methane produced m/z=15 and 16 fragments nearly at a 1 to 1 ratio. Therefore, the ammonia contribution to m/z=15 is only likely to be significant when the methane contribution is small and unlikely to influence m/z=16 thus ensuring that our approach remains accurate.

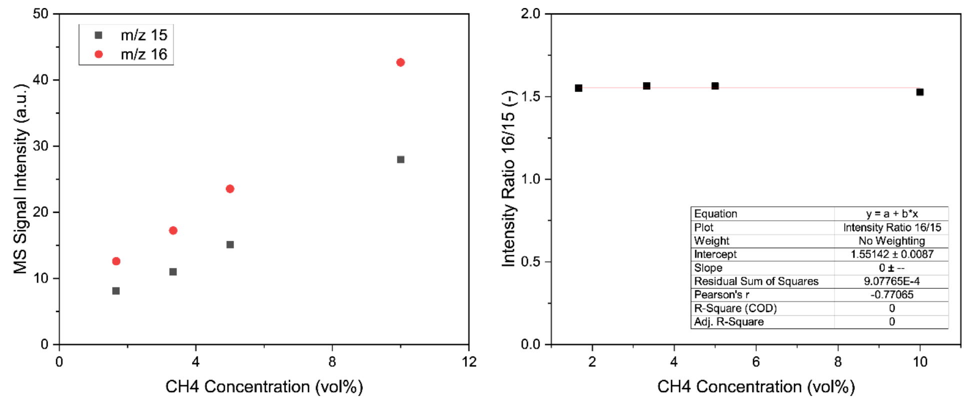

Figure S15. Methane mass fragment calibration in TPD. The mass fragment ratio of m/z=16 to m/z=15 was fitted across varying methane concentrations.

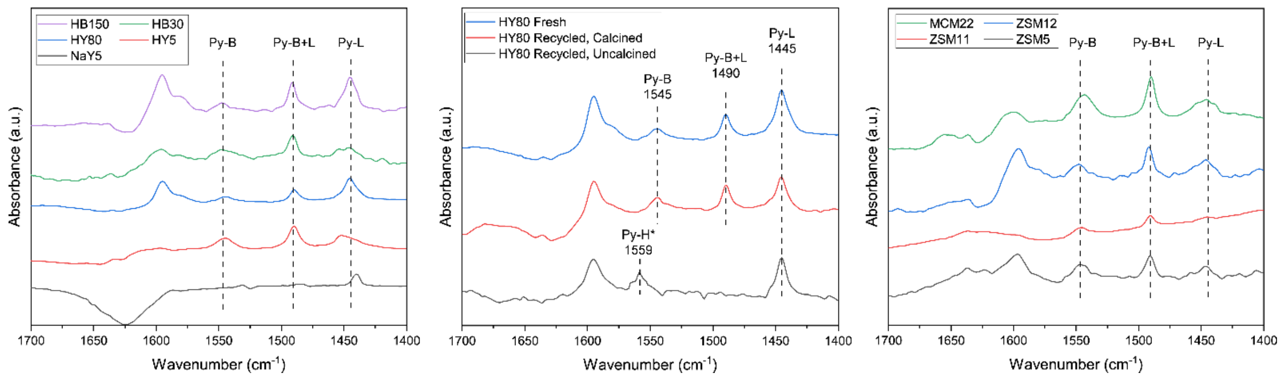

Figure S16. FT-IR spectra of various medium and large pore zeolites after pyridine adsorption. B: Brønsted acid. L: Lewis acid. H*: proton donated by surface contaminants.

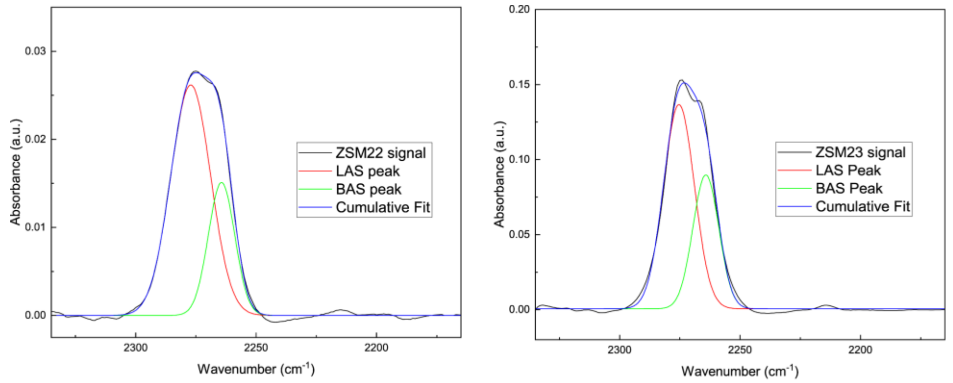

Model	Gauss	
Equation	y=y0 + (A/(w*sqrt(pi/2)))*exp(-2*((x-xc)/w)^2)	
Plot	Peak1(Absorbance)	Peak2(Absorbance)
y0	0 ± 0	0 ± 0
xc	2277.16452 ± 0.65976	2264.37194 ± 0.4761
w	16.5658 ± 0.75382	10.92829 ± 0.65384
A	0.54351 ± 0.03925	0.20679 ± 0.0384
Reduced Chi-Sqr	6.20646E-7	
R-Square (COD)	0.9873	
Adj. R-Square	0.98708	

Model	Gauss	
Equation	y=y0 + (A/(w*sqrt(pi/2)))*exp(-2*((x-xc)/w)^2)	
Plot	Peak1(Absorbance)	Peak2(Absorbance)
y0	6.04507E-4 ± 2.33988E-4	6.04507E-4 ± 2.33988E-4
xc	2275.38489 ± 0.66614	2264.21817 ± 0.72312
w	12.80519 ± 0.66178	10.77248 ± 0.65974
A	2.18342 ± 0.2245	1.20358 ± 0.22285
Reduced Chi-Sqr	1.41534E-5	
R-Square (COD)	0.98827	
Adj. R-Square	0.98803	

Figure S17. FT-IR spectra of small pore zeolites ZSM22 and ZSM23 after adsorption of deuterated acetonitrile. The peak fitting statistics are provided below each plot. BAS: Brønsted acid site. LAS: Lewis acid site.

5.3 Catalyst regeneration and Thermogravimetric analysis (TGA)

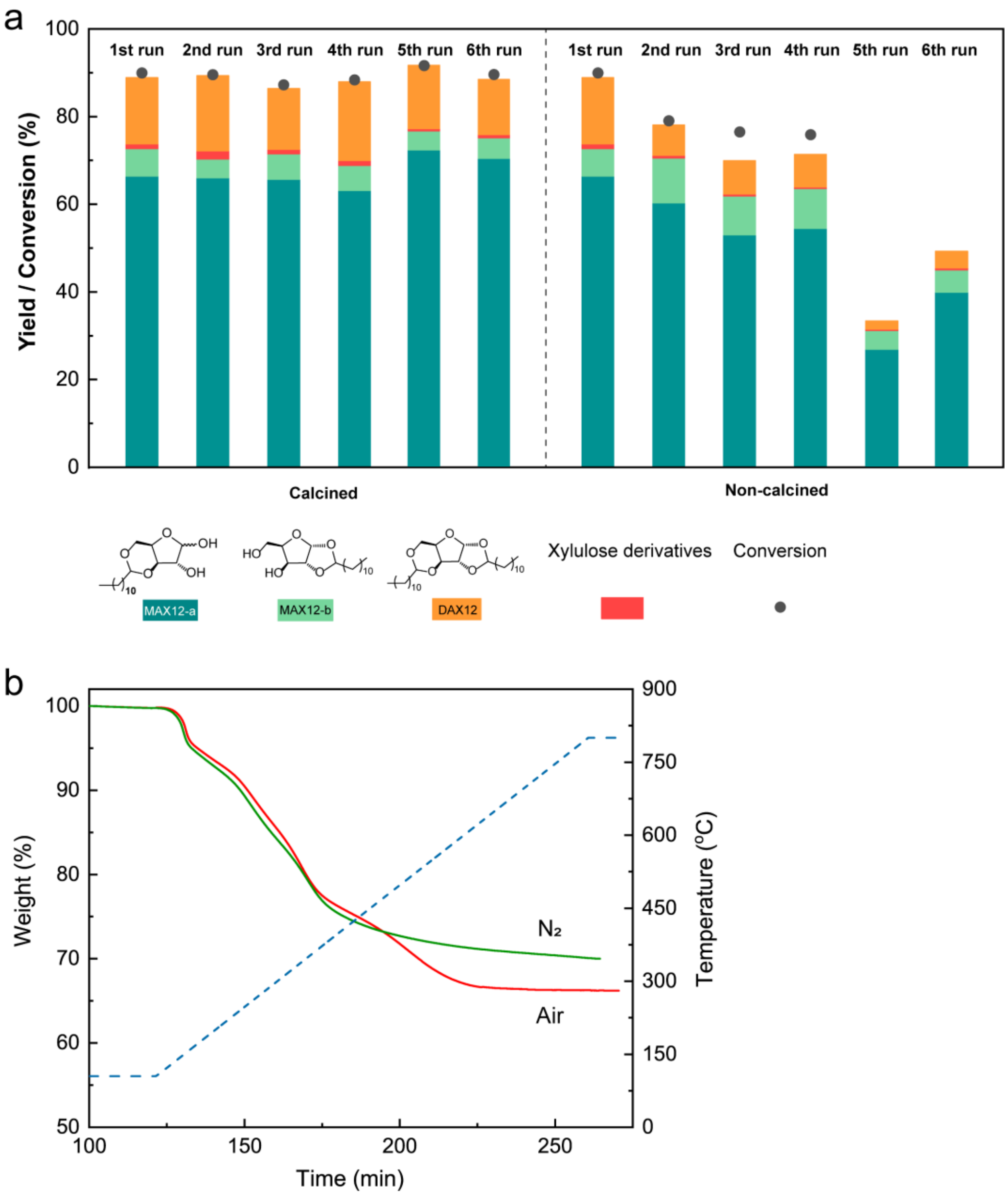

Figure S18. (a) Recyclability of HY80 with and without calcination between each run, and (b) Thermogravimetric analysis (TGA) profiles of used HY80 under air and N_2.

*Calcined: calcination (air, 550 °C, 6 h) after each run; non-calcined: washed with dioxane and dried in vacuum overnight.

The deactivation of catalysts during the xylose acetalization reaction, probably due to the accumulation of organic residues, was assessed with Thermogravimetric Analysis (TGA). Given that the acetalization reaction occurs in organic solvents and at low temperatures, the formation of graphitic or amorphous coke is unlikely. Instead, larger condensed molecules may form on acidic sites, blocking micropores.

The spent zeolite was filtered from the reaction mixture, washed with dioxane, and dried before TGA. Under an inert nitrogen atmosphere, the TGA revealed that carbohydrate pyrolysis initiated at 150 °C, as evidenced by a significant mass loss (approximately 30% at 800 °C) and the detection of methane (m/z = 15) during NH_3-TPD. Under an air atmosphere, a distinct calcination process also commenced at 150 °C, resulting in a mass loss of approximately 34%. This calcination process reached a plateau at around 600 °C. Based on the weight profile during calcination, each catalyst regeneration cycle was conducted at 550 °C for 6 h to effectively burn off the organic deposits while preserving the zeolite framework.

The regeneration protocol was implemented as follows: after each catalytic cycle, the zeolite catalyst was separated by centrifugation and washed three times with fresh dioxane (3 × 5 mL) under sonication to remove organic residues. The washed catalyst was then dried at 45 °C under vacuum for 15 h. This dried catalyst was reused in the next catalytic run without further treatment (referred to as "non-calcined"). For the calcined version, the dried catalyst was subsequently heated in air at 550 °C for 6 h in a muffle furnace and cooled to room temperature before reuse. To minimize cumulative material loss over multiple runs, duplicate reactor setups were prepared in parallel at the beginning of the recycling experiment. Catalyst amounts were not normalized between runs, as nearly quantitative recovery was consistently achieved.

Notably, as depicted in Figure S18, the calcination process effectively restored the reactivity of the catalysts thanks to their good thermal stability. The reduction in acid density observed in the used, non-calcined catalysts is likely attributed to the presence of organic residues, which could be successfully removed through the calcination process. The increased number of recycling and regeneration did not affect the xylose conversion and product yields, provided that the catalyst was regenerated between reactions. However, conversion significantly dropped between cycle 4 and 5 when the catalyst was never regenerated. There also existed unreacted sugar after the reaction upon visual examination. This amount of sugar could not be effectively dissolved with organic solvents and washing with water could have damaged the zeolite structure and was avoided. The sugar residue was mixed with zeolite powder, making physical removal difficult. Therefore, the unreacted sugar was kept with the recycled zeolite for the 6th run, introducing more substrate at the beginning of the reaction. This explains why the perceived product yields in the 6th run were higher than in the 5th run. Due to this difficulty in residual xylose quantification, the xylose conversion was omitted for the last two reaction cycles for the never regenerated zeolite. Nonetheless, the sharp difference in the activities of zeolites with and without inter-reaction regeneration clearly demonstrated the effectiveness of simple calcination to regenerate the catalyst.

Table S6. Textual and acidic properties of fresh, never regenerated, and regenerated HY80 zeolites

Catalyst	S_{BET} (m^2/g)	Micropore area (m^2/g)	Total Pore volume @0.95 p/p_0 (cm^3/g)	Micropore volume (cm^3/g)	LAS density (µmol/g)	BAS density (µmol/g)
**HY80 fresh**	784.3	558.3	0.58	0.29	80.2	33.9
**HY80 6 times regenerated**	694.4	424.2	0.38	0.18	85.0	34.1
**HY80 6 times never regenerated**	413.4	226.8	0.23	0.09	64.5	0.0

BAS stands for Brønstad acid site, and LAS for Lewis acid site.

We compared the Py-FTIR spectra of fresh HY80, recycled but regenerated HY80, and recycled but never regenerated HY80 (Figure S16). After being recycled 6 times, the FTIR spectrum of the catalyst with inter-reaction regeneration was largely similar to the fresh catalyst. However, significant difference can be seen with the never regenerated catalyst: the disappearance of the absorption bands at 1490 cm^{-1} and 1545 cm^{-1} could be attributed to a near complete contamination of BAS by organic compounds. A new peak at 1559 cm^{-1} also appears, likely due to the interactions between pyridine and organic contaminants. The lack of the peak at 1490 cm^{-1} and 1545 cm^{-1} resembles the Py-FTIR spectrum of NaY5, which does not have BAS. This results points to interactions between BAS and the contaminants as a main catalyst deactivation mechanism. Despite a total loss of active sites for the isolated catalyst, this catalyst can still lead to an over 30% product yield, suggesting that some contaminants can dissociate with the BAS during the reaction. However, this contamination-dissociation process is unlikely to be fully reversible, leading to gradual catalyst deactivation. The BAS could be restored to the original level with inter-reaction calcination. Although Lewis acid sites did not appear to play a role in catalyzing this reaction, the loss in the Lewis acid site density also suggested that there were interactions between the organic contaminants and Lewis acid sites.

5.4 XRD (X-ray Powder Diffraction)

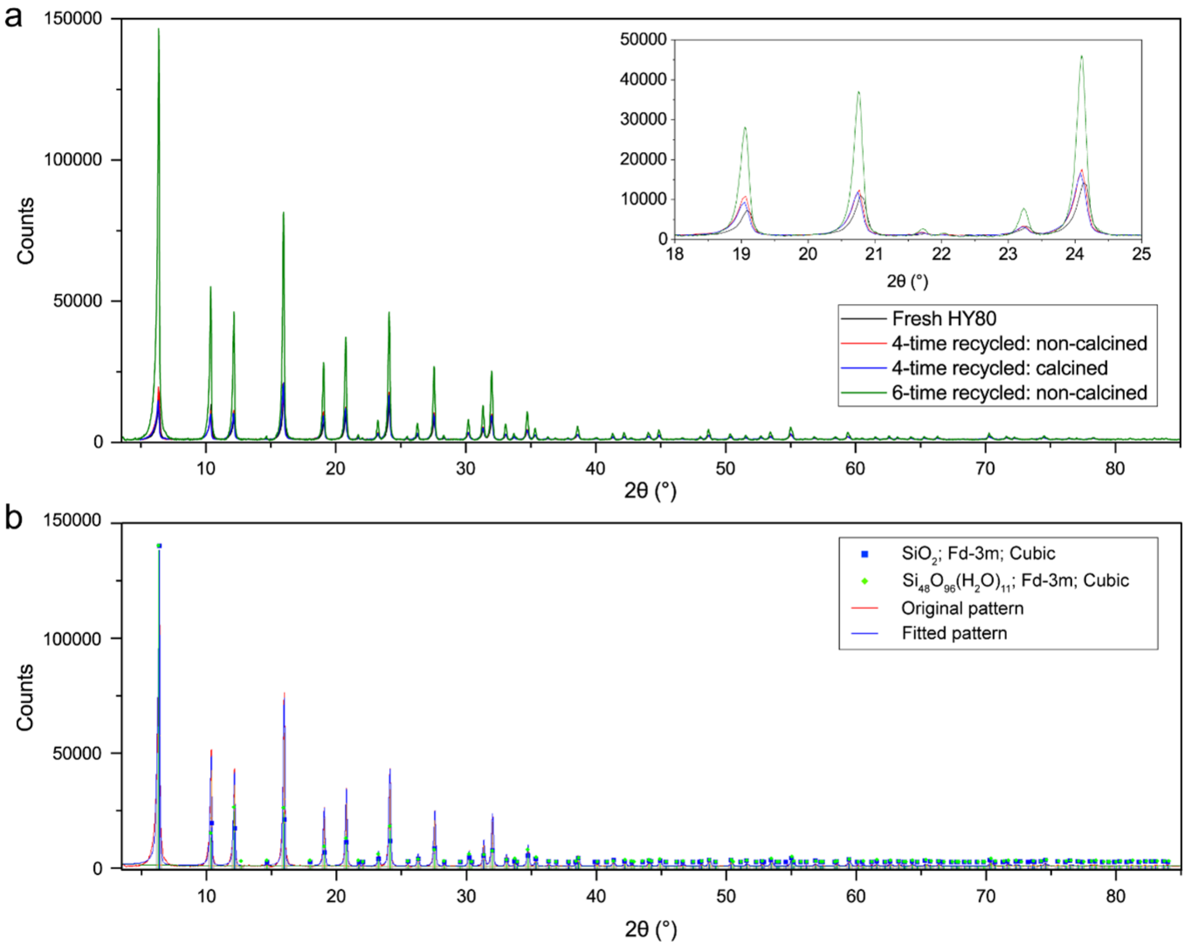

Figure S19. (a) X Ray Diffraction (XRD) Analysis of fresh and after 4th run (calcined, and non-calcined) and after 6th run (calcined) HY80. (b) An example XRD pattern of 6-time recycled HY80 with inter-reaction regeneration fitted to quantify the phase composition after Rietveld refinement.

Table S7. Crystalline compositions of prestine and recycled HY80 zeolite

	SiO_2	$Si_{48}O_{96}$ $(H_2O)_{11}$
Fresh HY80	66%	34%
4-time recycled HY80 (both calcined and non-calcined)	55%	45%
6-time recycled HY80 (calcined)	55%	45%

The crystallinity of zeolites following multiple calcination cycles was checked with polycrystalline X-ray diffraction (PXRD) on a Panalytical Empyrean powder diffractometer, equipped with Cu *K*α X-ray source and PIXCel^{1D} detector (Figure S19). Rieltveld refinement was performed before phase quantification. Peaks in the PXRD patterns of both calcined or non-calcined used zeolites (blue, red, and green) are clearly shifted to lower angles compared to peaks in the PXRD pattern of the fresh HY zeolite (black). According to the

Bragg's Law, $n \cdot \lambda = 2 \cdot d \cdot \sin(\theta)$ (where θ denotes the diffraction angle, λ represents the wavelength of X-rays, and d stands for the lattice spacing), diffraction peaks shifted to lower 2θ values indicates a slight expansion of the crystal lattice.

One plausible explanation for this phenomenon is the increased presence of crystalline water within the crystal structure. During the reaction, water molecules could be captured in the crystal lattice as guest molecule, leading to alterations of the lattice parameters[10]. Remarkably, this guest water appears to withstand the 550 °C calcination process (Table S7). The composition of the hydrated silica phase also did not change between the 4-time and 6-time recycled material.

5.5 SSNMR (Solid-state NMR)

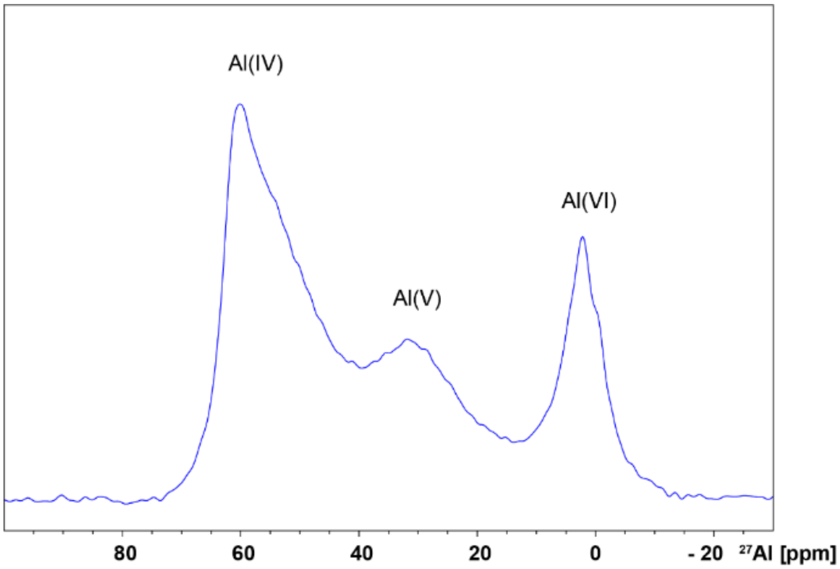

Figure S20. Example ^{27}Al SSNMR on fresh HY12 catalyst. The chemical shift assignment of different aluminum phases is based on Li *et al*[11].

The acid sites of zeolite are related to the different local structures of surface aluminum. Solid-state NMR with magic-angle spinning (MAS) was used to identify the coordination number of aluminum species and quantify their compositions as each Al species can be resolved at a distinctive chemical shift. An example ^{27}Al spectrum of HY12 zeolite is shown in Figure S20. The other spectra are compiled in the Zenodo database. Spectra were recorded on calcined zeolite and are without additional hydration. Despite using the MAS technique to enhance signal resolution, peak broadening may be attributed to the amorphous phase in the fine zeolite powder and strong quadrupolar interactions[12]. The corresponding chemical shifts were retrieved from literature values[13–15]. Among the tested zeolites, aluminum exists as i) fully four-coordinated framework Al, Al (IV), with a chemical shift around 60 ppm, ii) five-coordinated extra-

framework Al, Al(V), with a chemical shift around 30 ppm, and iii) six-coordinated extra-framework Al, Al (VI), with a chemical shift around 0 ppm. The framework aluminum species, Al (IV), is known to give rise to Brønsted acid sites at the bridging oxygen between Al and Si atoms, whereas the extra-framework Al species (Al (V) and Al (VI)) can lead to Lewis acid sites[11]. Due to the different coordination numbers and different forms of Al species, the different types of extra-framework Lewis acid sites may have different strengths and, therefore, favor different reactions[14][16][17]. The location of these extra-framework Al species also affects their accessibility and, hence, their effective contribution to the reactions[18]. So far, studies on biomass valorization with zeolites mostly focused on differentiating catalytic effect of Brønsted and Lewis acid sites, whereas the role of coordination structures around extra-framework Al that constitutes Lewis acid sites received little attention. Though identifying the interactions between different aluminum species and xylose and intermediates is well beyond the scope of this work, future work in this direction could be of particular interest for targeted catalyst design and synthesis to optimize the yield and selectivity of a desired product.

NMR peak integration was performed using Dmfit[19] to calculate the compositions of Al species in the zeolite samples (Table S8). Interestingly, comparing with the product distribution reported in Figure 4b in the main text, the best performing HY80 zeolite that produced the least xylulose derivatives showed no Al (VI) in its structure, whereas the HY zeolites yielding more xylulose derivatives contained more Al (VI). Additionally, the Al (V) fractions in HY80 and HY60 were not lower than those in HY30 and HY5.2, but the former only produced a small amount of xylulose derivatives. On the other hand, in absence of Al (V) species in Hβ150 and Hβ38, they still produced a large amount of xylulose derivatives. Hence, it may be hypothesized that Lewis acid sites that stemmed from the extra-framework Al (VI) species was the main active site that catalyzed xylose isomerization to xylulose.

Table S8. The compositions of aluminum phases in the tested zeolites

	Species molar compositions (%)		
	Al (IV)	Al (V)	Al (VI)
**Hβ25**	64.7%	10.6%	24.7%
**Hβ30**	64.6%	19.1%	16.3%
**Hβ38**	78.3%	0.0%	21.7%
**Hβ150**	95.8%	0.0%	4.2%
**MOR19**	84.0%	0.0%	16.0%
**NaY5**	100.0%	0.0%	0.0%
**HY5.2**	40.9%	43.3%	15.9%
**HY12**	66.8%	18.4%	14.9%
**HY30**	61.3%	21.3%	17.3%
**HY60**	47.2%	41.6%	11.2%
**HY80**	54.0%	46.0%	0.0%

5.6 Zeolite acid strength

Temperature programmed desorption of ammonia (NH_3-TPD) was used to compare the acid strength of various zeolites used in this study. The temperature at which the probe molecule desorbs from an acid site is known to be well correlated with acid strength[20]. The zeolites in this study mostly exhibit three desorption peaks (Figure S21): a peak below ca. 300°C corresponding to a weak acid site, a medium strength site from which NH_3 desorbs between 300 and 600°C, and a strongly acidic site corresponding to a desorption peak above ca. 600°C, which is consistent with literature reports[21]. The weak acid site is often considered to have trivial catalytic activities[22]. Indeed, NaY5 only has low strength acid sites (Figure S22) and was found inactive catalyzing acetalization of sugars and polyols (Figure 4b in main text). The large medium-temperature peak and the smaller high-temperature peak can be assigned to NH_3 desorption from strong Brønsted and Lewis acid sites which have catalytic activities, as is well reported in literature[21][22][23]. Notedly, the acid sites of HY80 and HBeta150 are among the weakest, while those of ZSM5 and ZSM23 are among the highest, which order concurs with other literature reports [20].

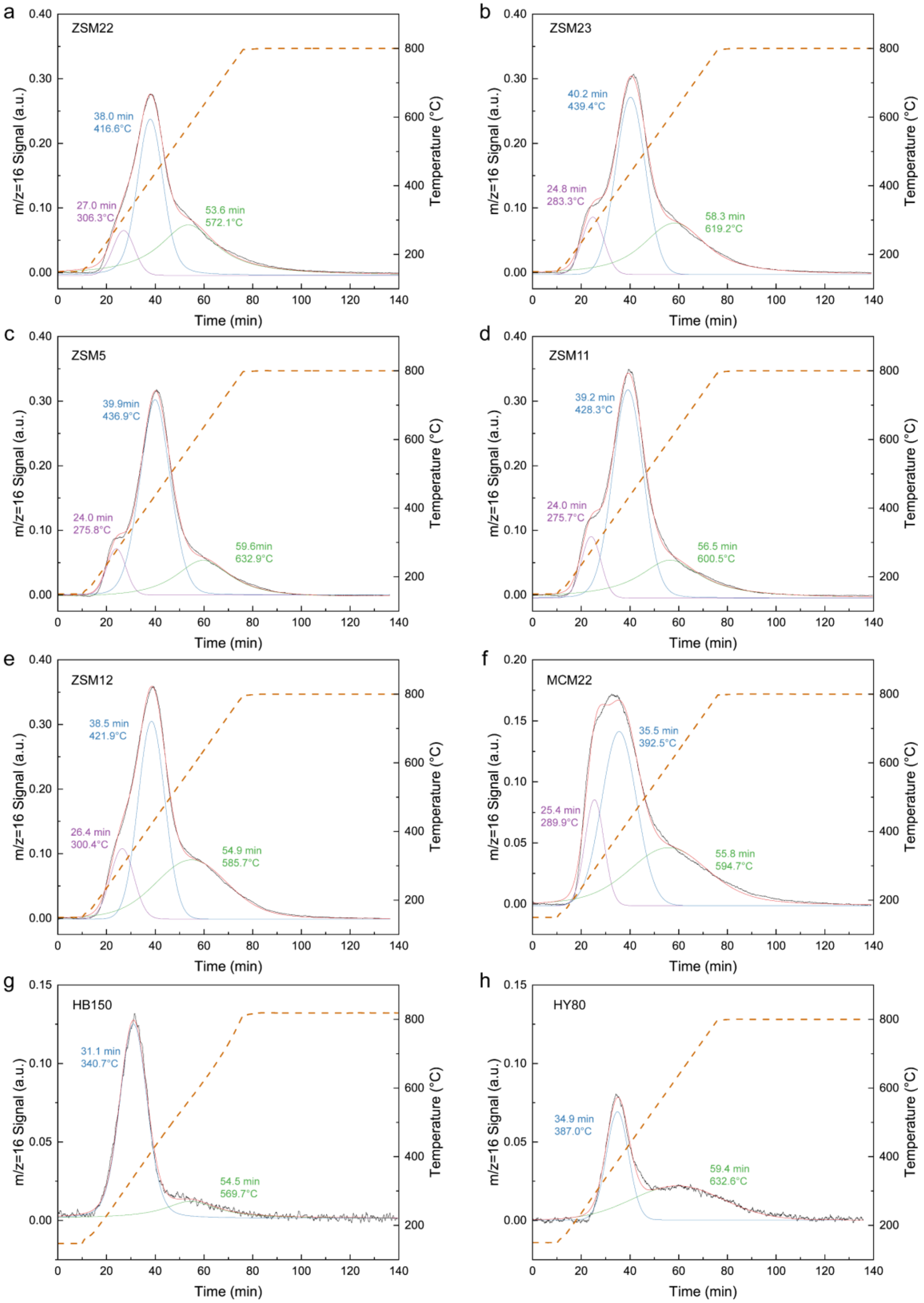

Figure S21. Temperature programmed desorption (TPD) of NH_3 on the zeolites shown in Figure 2. Desorption peaks were deconvoluted using the Voigt function. The maximum desorption time and the corresponding temperature are labelled for each deconvoluted peak.

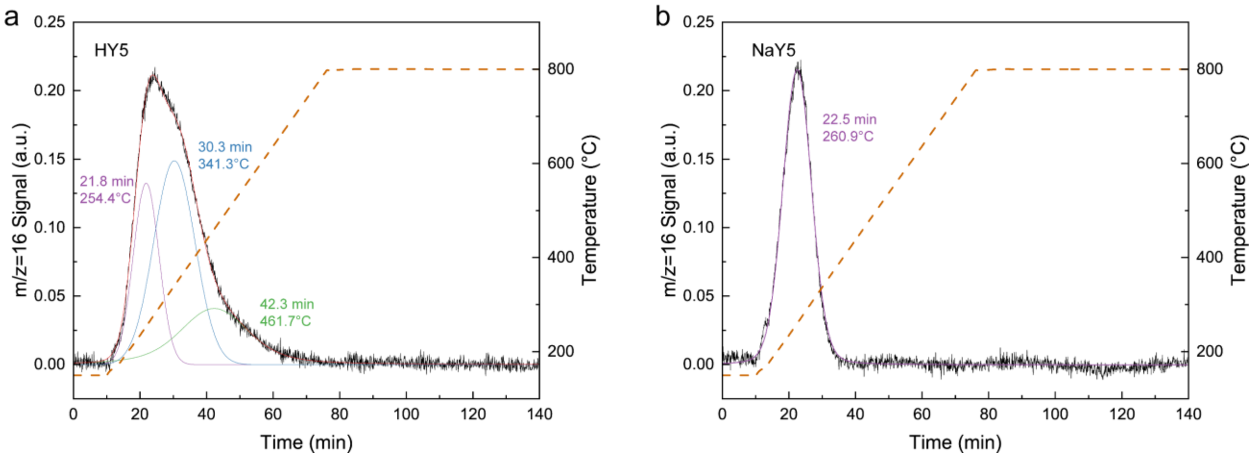

Figure S22. Comparison of NH_3-TPD profiles of HY5 and NaY5.

6. Substrate and zeolite dimensions and selectivity correlations

6.1 Substrate and zeolite dimensions

Table S9. Substrate kinetic diameter estimations

Substrate	Kinetic diameter, σ (Å)	Ref.
Mesoerythritol	5.6	Eq. S5
Threitol	5.6	Eq. S5
Pentaerythritol	6.0	Eq. S5
Xylitol	6.6	[24]
D-xylose	6.8	[25]
L-arabinose[a]	6.8	-
D-Glucose	8.6	[24]

The kinetic diameter (σ) is estimated from the properties of the fluid at the critical point (*c*), shown in Eqs. S4 and S5 according to Bird *et al.*[26]:

Equation S4: $\sigma = 0.841(V_c)^{1/3}$, or Equation S5: $\sigma = 2.44(T_c/P_c)^{1/3}$,

where V_c is the critical volume in $cm^3\ mol^{-1}$, T_c is the critical temperature in Kelvins, and P_c is the critical pressure in atmospheres. Critical point data were obtained from the Cheméo database[27].

[a]The kinetic diameter of L-arabinose is assumed to be similar to that of D-xylose.

Table S10. Physico-chemical properties of zeolites used in this study obtained from the International Zeolite Association[28] and through Ar physiosorption isotherms.

Catalysts		ZSM22	ZSM23	ZSM5	ZSM11	ZSM12	MCM22	BETA*	Y
Topology		TON	MTT	MFI	MEL	MTW	MWW	BEA	FAU
Maximum diameter of a sphere that can diffuse along: (Å)	a	2.31	5.07	4.7	5.19	1.27	4.92	5.94	7.35
	b	1.56	2.2	4.46	5.19	5.68	4.92	5.94	7.35
	c	5.11	1.53	4.46	5.19	2.41	2.6	5.94	7.35
Internal pore diameter (Å)		5.71	6.19	6.36	7.72	6.08	9.69	6.59	11.24
Ring size (MR)		10	10	10	10	12	10, 12	12	12
Average pore diameter (Å)[a]		5.65	5.86	6.03	6.03	6.30	6.54	6.92	7.78
Channel dimensions (Å)		4.6×5.7	4.5×5.2	5.1×5.5<br>5.3×5.6	5.3×5.4	5.7×6.1	4.0×5.5<br>4.1×5.1	6.6×6.7<br>5.6×5.6	7.4×7.4
SiO_2/Al_2O_3 ratio		65-80	100	80	50	100	7	150	80

*BETA zeolite has a disordered framework structure. The dimensions of the A polymorph are shown here as representative values.
[a]The average pore diameter was obtained through the pore size distribution calculated from Ar physisorption isotherms (SI, Section 5.1.5), and the pore size distributions are present in Figure S23.

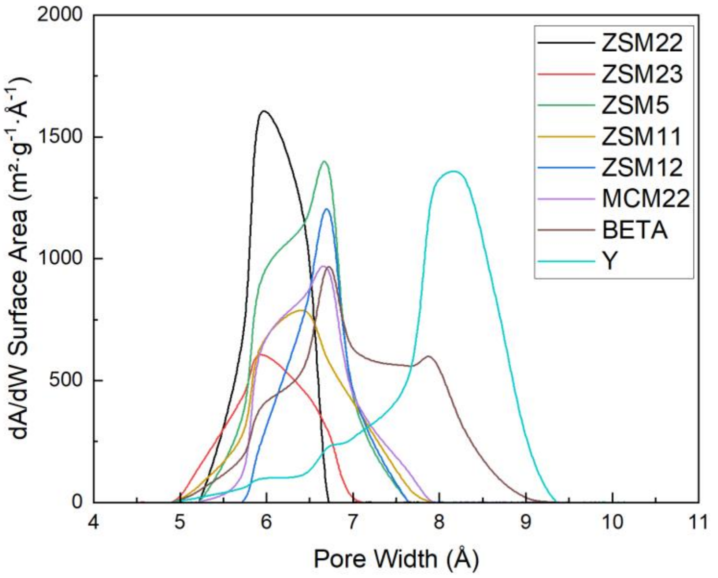

Figure S23. Pore size distributions of the zeolites used in this study

6.2 Correlations between pentaerythritol monoacetal selectivity and various zeolite properties

In order to comprehensively survey the effect of zeolite properties on monoacetal selectivity, we plotted monoacetal selectivity against various zeolite properties, including the average pore size, the total Brønsted acid site (BAS) loading in the reaction batch, the total BET area, and the average crystallite size of each catalyst (the characteristics are listed in section 5.2) used in the corresponding reaction with pentaerythritol and dodecanal (Figure 2 in the main text). The Lewis acid loading was not considered as we know from experience that Lewis acid sites are not active in forming multi-cyclic acetals. We clearly see that only the average pore size is visibly correlated with monoacetal selectivity. To further quantify the correlation result, we performed a two-tailed Spearman's correlation test between monoacetal selectivity and the selected zeolite property (Table S11). There existed a strong correlation between monoacetal selectivity and the zeolite pore size, with a correlation coefficient of -0.95 at a confidence level of 99.95%. Correlations with all the other zeolite properties had confidence levels below 60%, indicating poor correlations. Correlations between product yields and BAS loading also lacked statistical significance.

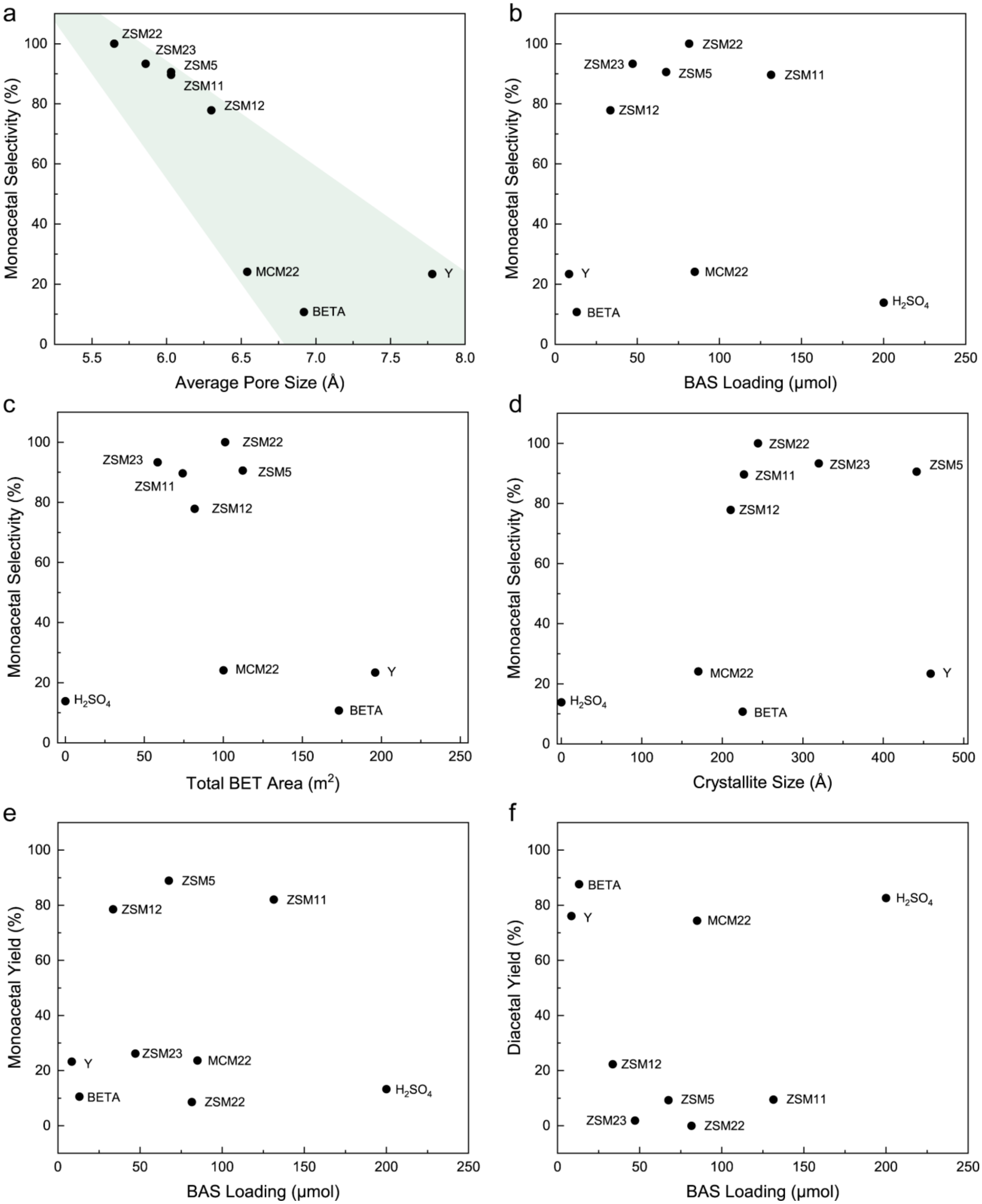

Figure S24. Effect of various zeolite properties on (a-d) monoacetal selectivity, (e) monoacetal yield and (f) diacetal yield in the reaction between pentaerythritol and dodecanal. Only the correlations between monoacetal selectivity and average pore size is shown in (a) as it was the only statistically significant correlation. The shaded area in panel a serves only to highlight the trend in the data.

Table S11. Correlation statistics between monoacetal selectivity and zeolite properties

Correlation Variables	Spearman's Correlation Coefficient, r_s	Degree of Freedom	P Value
a. Pore size vs. monoacetal selectivity	-0.95	6	$2.6x10^{-4}$
b. BAS loading vs. monoacetal selectivity	0.17	7	0.67
c. BET area vs. monoacetal selectivity	-0.18	7	0.64
d. Crystallite size vs. monoacetal selectivity	0.45	7	0.22
e. BAS loading vs. Monoacetal yield	0.083	7	0.83
f. BAS loading vs. Diacetal yield	-0.17	7	0.67

6.3 Correlation of Knudsen diffusion with product yields and selectivity

Table S12. Knudsen diffusion coefficients of molecule-zeolite pairs in Figure 2

**Molecules**	**MW (g/mol)**	**Knudsen diffusion coefficient x10^8, D_{kn} (m^2/s)**							
		**ZSM22**	**ZSM23**	**ZSM5**	**ZSM11**	**ZSM12**	**MCM22**	**BETA**	**Y**
Pentaerythritol	136.15	4.3	4.5	4.6	4.6	4.8	5.0	5.3	5.9
MAE-12	302.46	2.9	3.0	3.1	3.1	3.2	3.4	3.6	4.0
DAE-12	468.77	2.3	2.4	2.5	2.5	2.6	2.7	2.8	3.2

Table S13. Knudsen diffusion coefficients of molecule-zeolite pairs in Figure 3

Reactants	Molecules	MW (g/mol)	Knudsen diffusion coefficient x10^8, D_{kn} (m^2/s)	
			ZSM5	Y
Mesoerythritol	Mesoerythritol	122.12	4.9	6.3
	Monoacetal-12	288.43	3.2	4.1
	Diacetal-12	454.74	2.5	3.3
Threitol	Threitol	122.12	4.9	6.3
	Monoacetal-12	288.43	3.2	4.1
	Diacetal-12	454.74	2.5	3.3
Pentaerythritol	Pentaerythritol	136.15	4.6	5.9
	Monoacetal-12	302.46	3.1	4.0
	Diacetal-12	468.77	2.5	3.2
Xylitol	Xylitol	152.15	4.4	5.6
	Monoacetal-12	318.46	3.0	3.9
	Diacetal-12	484.77	2.4	3.1
D-Xylose	D-Xylose	150.13	4.4	5.7
	Monoacetal-12	316.434	3.0	3.9
	Diacetal-12	482.737	2.4	3.2
L-Arabinose	L-Arabinose	150.13	4.4	5.7
	Monoacetal-12	316.44	3.0	3.9
	Diacetal-12	482.75	2.4	3.2
D-Glucose	D-Glucose	180.16	4.0	5.2
	Monoacetal-12	346.48	2.9	3.7
	Diacetal-12	512.80	2.4	3.1

Table S14. Knudsen diffusion coefficients of molecule-HY80 pairs in Figure 4

Aldehydes	Products	MW (g/mol)	Knudsen diffusion coefficient x10^8, D_{kn} (m^2/s)
C1	MAX1	162.14	5.4
	DAX1	174.15	5.3
C3	MAX3	190.20	5.0
	DAX3	230.26	4.6
C5	MAX5	218.25	4.7
	DAX5	286.37	4.1
C8	MAX8	260.33	4.3
	DAX8	370.52	3.6
C10	MAX10	288.38	4.1
	DAX10	426.63	3.4
C12	MAX12	316.43	3.9
	DAX12	482.74	3.2
C18	MAX18	400.60	3.5
	DAX18	650.74	2.7

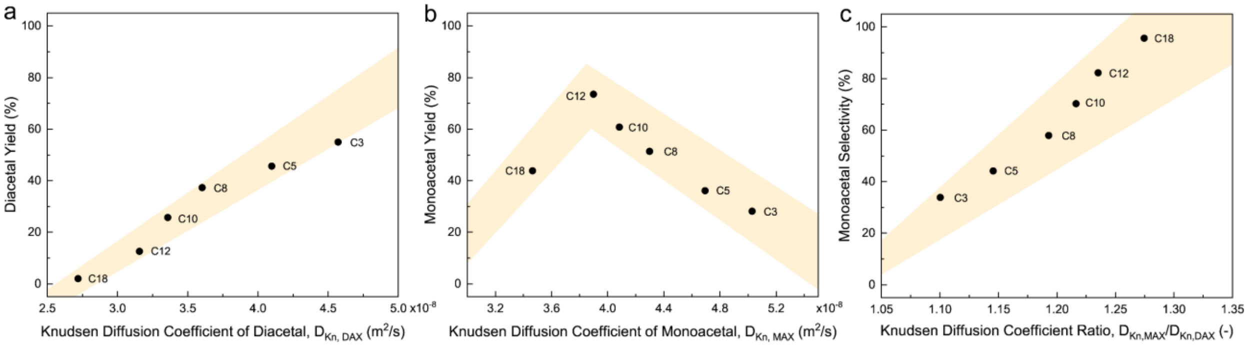

Figure S25. Correlations between (a) diacetal yields, (b) monoacetal yields, and (c) monoacetal selectivities during D-xylose acetalization with various aldehydes using HY80, and the Knudsen diffusion coefficients for the corresponding products in zeolite pores. The results correspond to the Figure 4 in the main text. The shaded areas serve only to highlight the trend in the data.

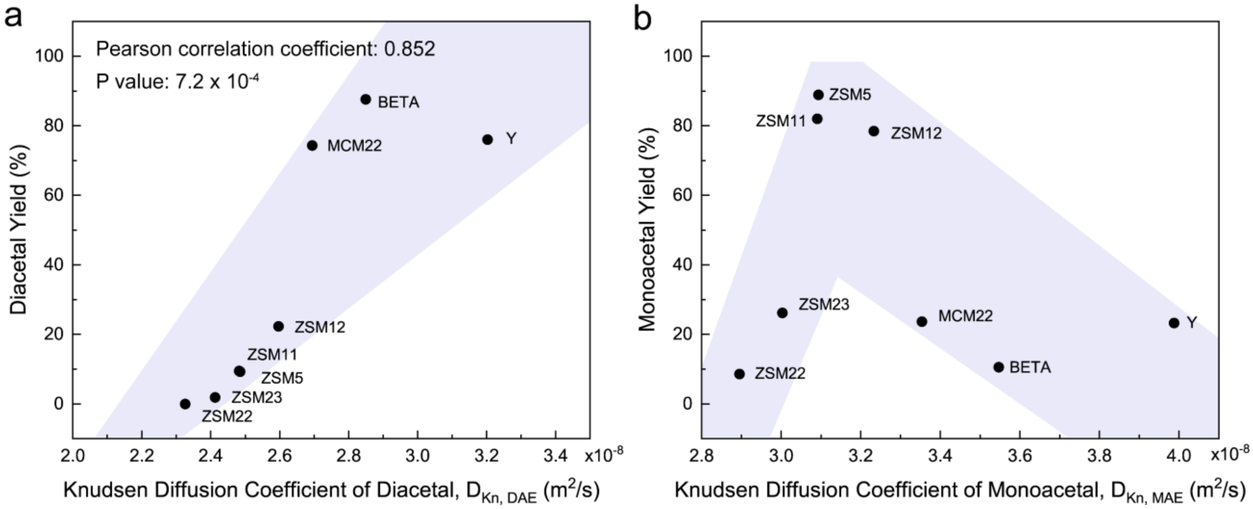

Figure S26. Correlations between the yields of (a) diacetal and (b) monoacetal during pentaerythritol acetalization with dodecanal, and their Knudsen diffusion coefficients in the respective zeolite pores. The results correspond to Figure 2 in the main text. The shaded areas serve only to highlight the trend in the data.

Although the reaction selectivity and yield correlate well with Knudsen diffusion coefficients within the same type of reactants (e.g., pentaerythritol acetalized by dodecanal in Figure 2, or xylose acetalized by linear aldehydes in Figure 4), direct comparison of these Knudsen diffusion coefficients for different types of reactants gives inconclusive results. For instance, the Knudsen diffusion coefficients were virtually the same for xylitol and xylose for their respective acetal-zeolite pairs, but their reaction yields were very different (Table S13). Xylose barely reacted using ZSM-5, while xylitol had a nearly full conversion. HY80 favoured monoacetals of xylose, while it favoured diacetals of xylitol. These differences and inconsistency with Knudsen diffusion results from the challenge of estimating intrinsic size effect of a molecule. The

Knudsen diffusion coefficient uses the molecular weight as an estimate, which means that it assumes the same size effect of all molecules of the same molecular weight without differentiation of specific structures (e.g. linear vs cyclic, axial vs. equatorial, bond rigidity, angular stiffness, etc.). For example, xylose and xylitol have very similar molecular weights, but different structures. Xylose is predominantly cyclic, while xylitol is linear. Although Knudsen diffusion coefficients can evidence the pore-size selectivity in this work, additional factors need to be considered to better define molecular sizes if one wishes to establish a comprehensive correlation in future studies.

7. Xylose acetalization reaction

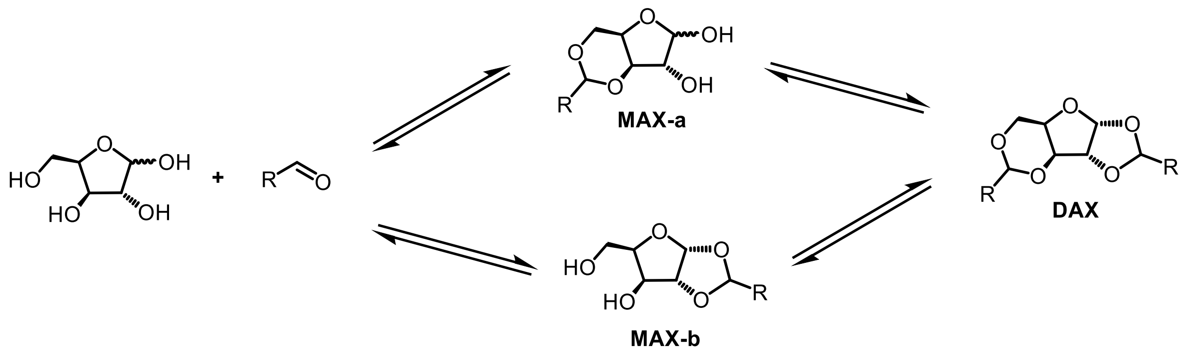

Scheme S1. Equilibrium reaction of xylose acetalization via MAX-a or MAX-b to DAX.

7.1 Reaction condition screening

The optimization procedure for producing monoacetalized xylose with a homogeneous acid catalyst (H_2SO_4) is shown in Figure S27. During our investigations, we observed an initial increase followed by a decrease in MAX12 yield as the reaction time progressed. Although a slight enhancement (approximately 25%) in the maximum MAX12 yield was achieved by reducing both the reaction temperature (from 80 °C to 65 °C) and the acid concentration (from 0.15 M to 0.02 M), this improvement remained limited. Furthermore, increasing the stoichiometric ratio of xylose to dodecanal from 1:2 to 1:1 led to low conversion due to equilibrium constraints (Figure S28). Additionally, when employing H_2SO_4 as the catalyst, we observed an increased occurrence of aldol condensation with dodecanal compared to zeolite catalysts, resulting in a lower mole balance. In general, DAX12 formation appears to be more favorable without the confinement effect imposed by microporous materials.

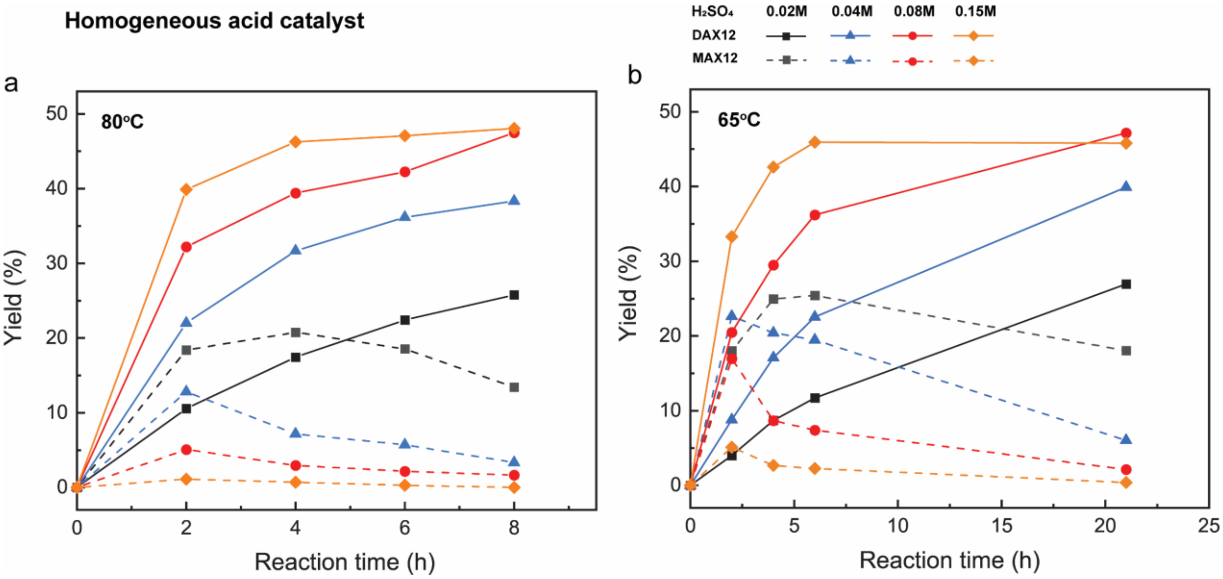

Figure S27. MAX12 and DAX12 yield with different concentration of H_2SO_4 acid catalyst and temperature. Reaction conditions: 80 °C or 65 °C, 1 : 2 equivalent (xylose : dodecanal).

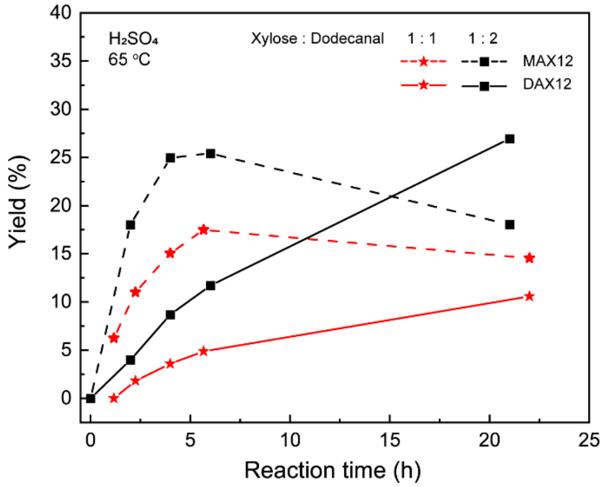

Figure S28. MAX12 and DAX12 yields with different stoichiometric ratios of xylose to dodecanal (1:1; 1:2) over 0.02M H_2SO_4 at 65 °C.

Likewise, the optimization of monoacetalized xylose using a heterogeneous acid catalyst (zeolite) is depicted in Figure S29. The reported yields are conservative, as they do not account for adsorbed products within the zeolite. Therefore, we refer to these values as "mole fractions" rather than "yields". Much like in homogeneous catalysis, we observed a similar trend in the mole fraction of MAX12 with HY80 zeolite, where it initially increased and then decreased with the progression of the reaction over time. However, what sets this apart is the selectivity (defined here as Eq. S6), which exhibited a noteworthy increase. In homogeneous catalysis, the selectivity stood at 74.2% (at 65 °C for 4.25 h) with a maximum yield of 25.0%,

while in heterogeneous catalysis (with zeolite washing during workup), it significantly improved to 88.4% (at 80 °C for 0.5 h) with a maximum yield of 67.2%.

$$\text{Equation S6: Selectivity (mol\%)} = \frac{\text{monoacetal (mol)}}{\text{monoacetal (mol)+diacetal (mol)}}$$

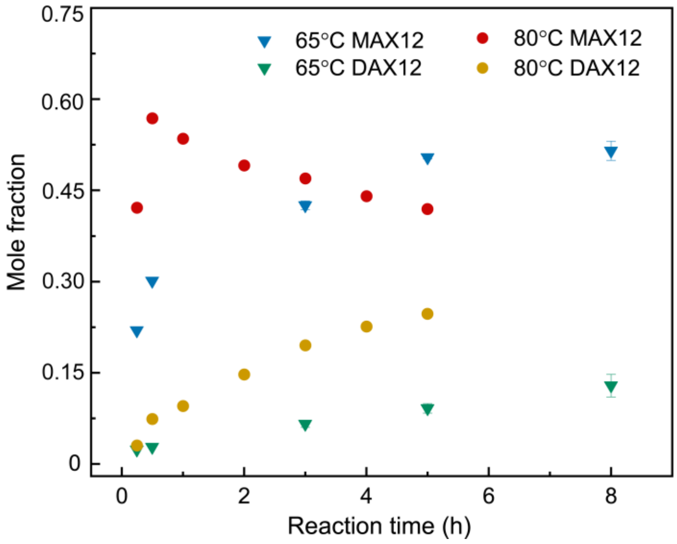

Figure S29. MAX12 and DAX12 mole fraction over HY80 along with the reaction time under different reaction temperature. Reaction conditions: 80 °C/65 °C, 1 : 2 equivalent (xylose : dodecanal).

*Y axis is not yield because the adsorbed products by zeolites weren't considered.

7.2 Solid acid (zeolite) catalyst screening

To study the xylose acetalization with dodecanal, we investigated zeolites with different topologies, SiO_2/Al_2O_3 ratios, and pore dimensions, such as 12 MR 3-D (3-dimentional) with cavities (HY), 12 MR 3-D without cavities (H-BETA), 12 MR 1-D (ZSM-12), 10 MR 3-D (ZSM-5, ZSM11), and 10 MR 1-D (ZSM-22). The reaction conversion and product yields are shown in Table S15. In the case of medium-pore microporous zeolites, such as ZSM5, ZSM11, ZSM22, and ZSM12, we did not observe significant product yields, likely due to the size of xylose being too large (xylose's kinetic diameter is 6.8 Å[29]) to enter the zeolite pores. The observed conversion might be attributed to xylose becoming lodged inside these pores. Large-pore microporous zeolites, like Y zeolites, particularly those with high SiO_2/Al_2O_3 ratios like HY60 and HY80, led to the highest MAX12 yields. Conversely, lower SiO_2/Al_2O_3 ratio HY zeolites tend to lead to higher yields of xylulose derivatives (identified by the HSQC spectra in Figure S30). These products likely resulted from xylose isomerization catalyzed by Lewis acids[29], followed by xylulose acetalization reactions catalyzed by Brønsted acids. Notably, xylulose is another example of a sugar capable of selectively forming monoacetals without the need for confinement. BETA zeolites even led to higher production of xylulose derivatives compared to MAX12 due to high Lewis acid site densities (Table S5).

To gain deeper insights into the influence of aluminum coordination structures within zeolite on Lewis acid sites, we conducted solid-state NMR analysis to quantitatively assess the composition of aluminum phases. We suggest that Lewis acid sites stemmed from the extra-framework Al (VI) species serve as the main active sites for catalyzing the isomerization of xylose to xylulose (see detailed discussion in SI section 5.5). Notably, across all these cases, as the SiO_2/Al_2O_3 ratio decreases, although overall acid density increases (as seen in NH_3-TPD, Figure S14), there is a decrease in reaction conversion and DAX12 yield. This phenomenon can be attributed to reduced Brønsted acid strength[30]. Another possibility lies in differences in catalyst hydrophobicity. Since water is a product of the reaction, its accumulation inside zeolite pores can shift the equilibrium towards reactants. Consequently, zeolites with higher hydrophobicity can theoretically achieve higher DAX12 yields by repelling water molecules out of the pores [31]. Hydrophobicity generally increases with the SiO_2/Al_2O_3 ratio due to the nonpolar nature of the Si-O-Si surface[32]. As exemplified by the high SiO_2/Al_2O_3 ratio Hβ150 zeolite, a higher DAX12 yield is observed compared to other catalysts.

Table S15. Acetalization reaction of xylose and dodecanal over different zeolites

Entry	Catalysts	Maximum diameter of sphere (Å)*	Conversion (%)	MAX12-a (%)	MAX12-b (%)	Xylulose derivatives	DAX12
1	HY80	7.35	89.2 ± 2.0	67.6 ± 1.4	5.3 ± 0.9	1.2 ± 0.1	13.5± 0.6
2	HY60		91.2	67.2	3.2	2.9	14.2
3	HY30		89.0	42.3	5.9	19.5	4.5
4	HY5.2		83.4	48.2	10.9	14.9	2.3
5	HY5.2§		91.4 ± 3.8	44.3 ± 7.1	5.9 ± 0.8	36.3 ± 2.4	1.4 ± 0.4
6	NaY5.2§		1.0 ± 0.0	0.1 ± 0.0	0.0 ± 0.0	0.0 ± 0.0	0.0 ± 0.0
7	Hβ150	5.95	91.1	27.4	3.4	22.8	28.7
8	Hβ38		90.3	29.7	4.4	32.3	9.6
9	Hβ30		93.4	35.2	4.0	28.4	14.9
10	Hβ25		76.3	24.8	4.8	22.2	2.4
11	ZSM5-80	4.7	20.1	2.8	0	0	0
12	ZSM11-50	5.19	17.3	0	0	0	0
13	ZSM22-80	5.11	21.7	0	0	0	0
14	ZSM12-100	5.68	22.2	3.2	1.3	0	0

Reaction conditions: 65 °C, 5 h, 1 : 2 mol. equivalent (xylose : dodecanal).
*Maximum diameter of sphere (Å), can diffuse along
Yields were obtained after zeolite washing
§Reaction conditions: 80 °C, 2 h, 1 : 2 mol. equivalent (xylose : dodecanal).
Error margins represent standard deviations from three independent experiments.

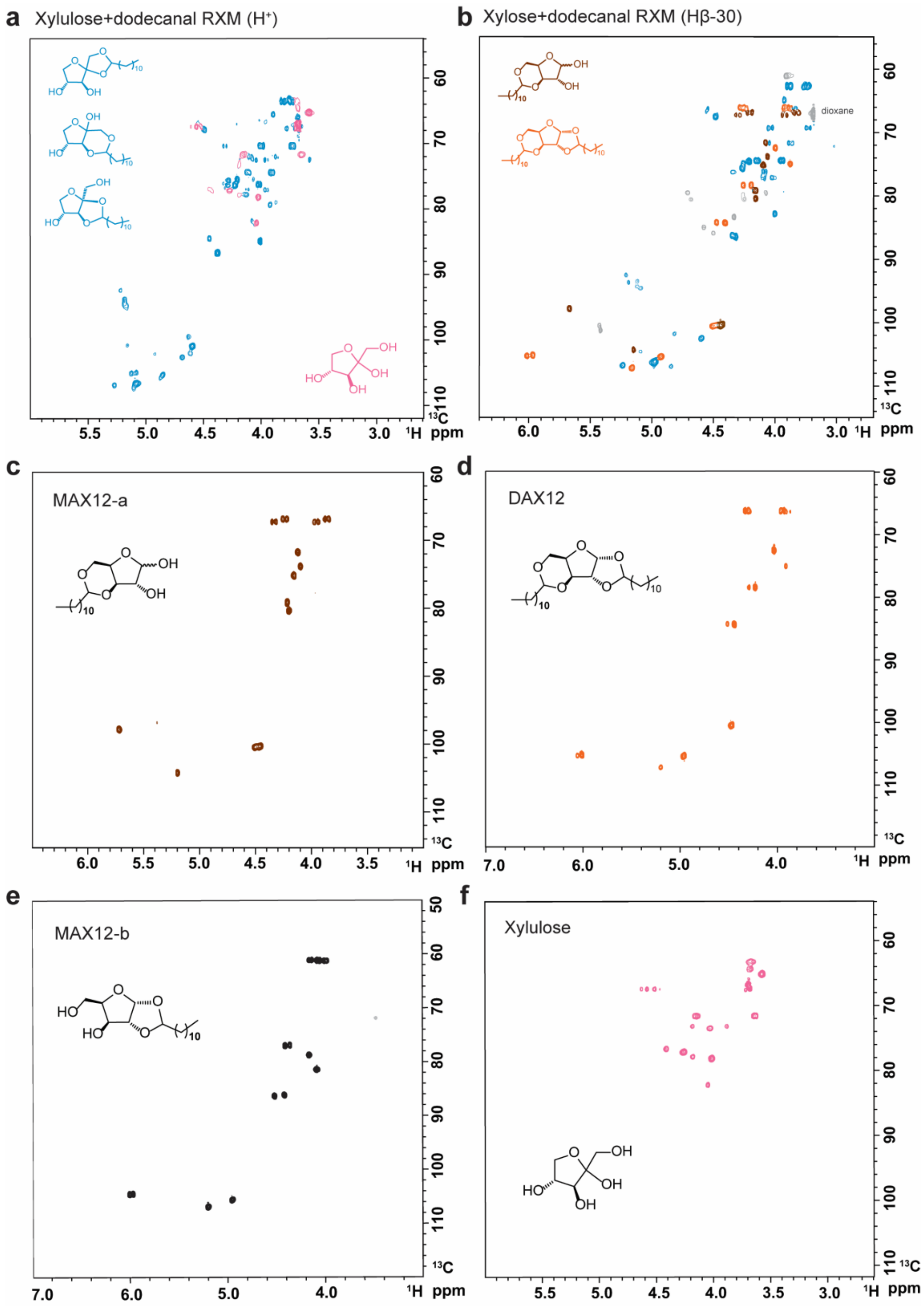

Figure S30. (a) HSQC spectrum of the reaction mixture of xylulose and dodecanal. (b) HSQC spectrum of the reaction mixture of xylose and dodecanal catalyzed by Hβ-30. (c) HSQC spectrum of MAX12-a. (d) HSQC spectrum of DAX12. (e) HSQC spectrum of MAX12-b. (f) HSQC spectrum of xylulose.

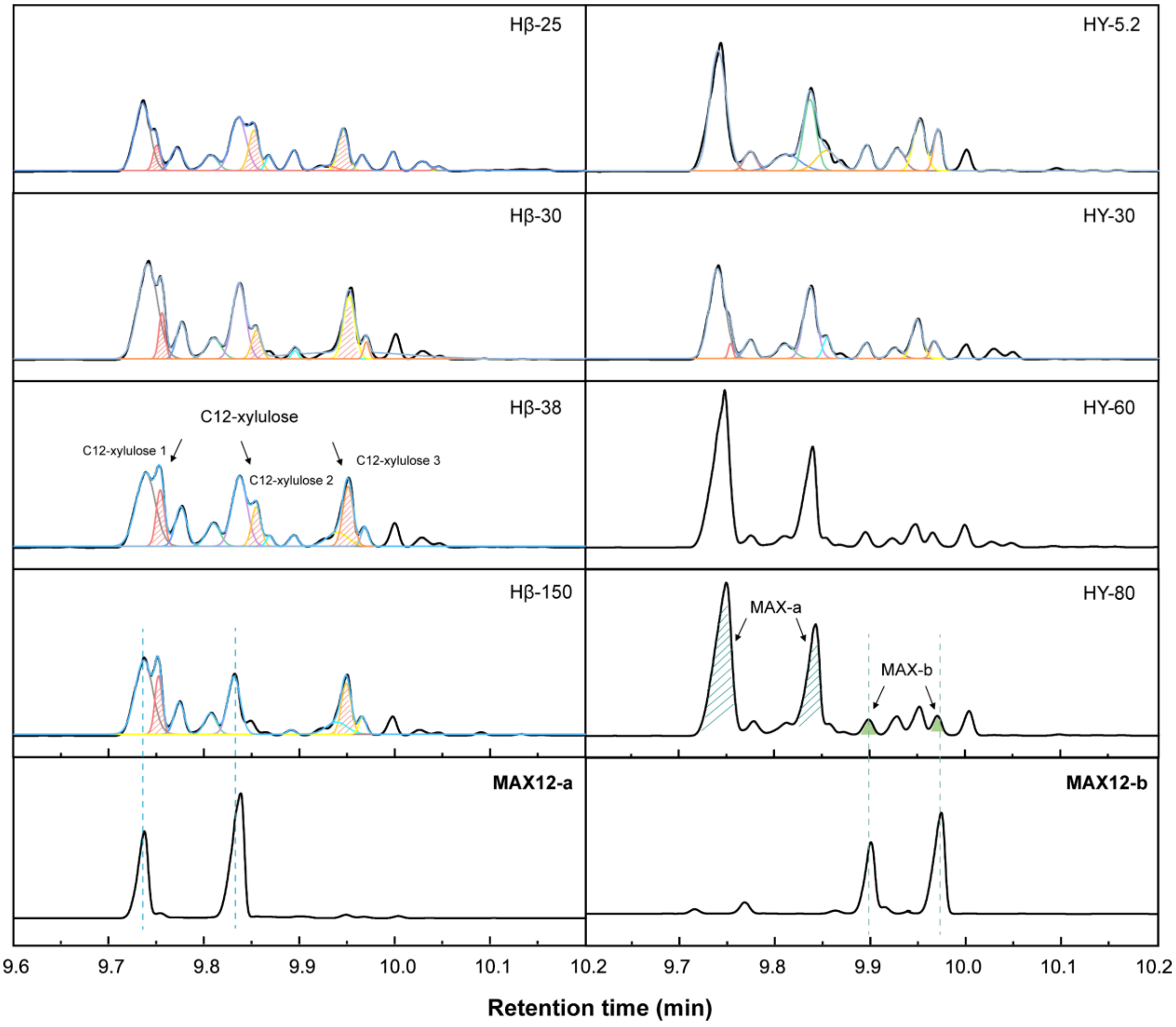

Figure S31. Assigning product species by deconvoluting GC-FID peaks. Peak assignment of MAX12-a and MAX12-b were confirmed based on retention time alignment with the reference compounds, and xylulose derivative identification via GC-MS mass spectra (refer to Figure S32).

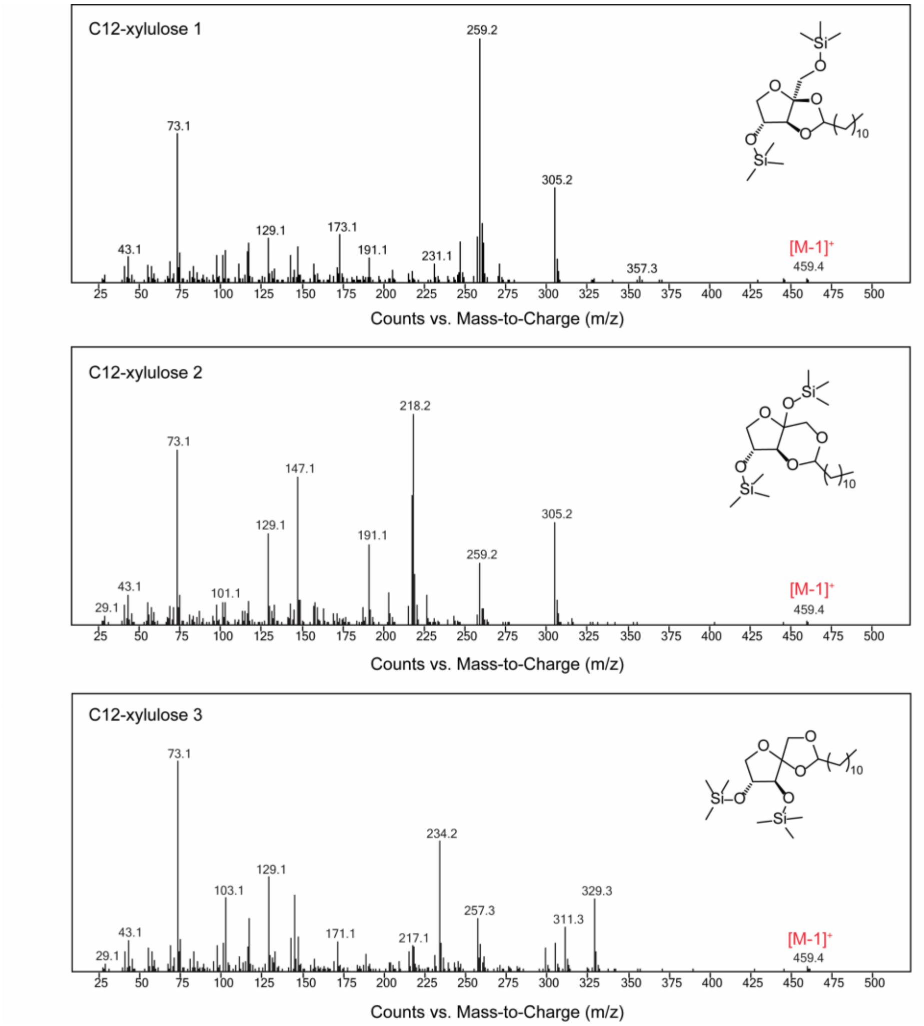

Figure S32. Mass spectra of GC peak with retention times of 9.76 min (C12-xylulose 1), 9.86 min (C12-xylulose 2), 9.95 min (C12-xylulose 3) (see corresponding peaks in Figure S31).

7.3 Xylose acetalization of with different aldehydes over HY80 and H_2SO_4

We compared the acetalization reaction of xylose with different aldehydes over H_2SO_4, and HY80—the large-pore microporous zeolites with lower Lewis acid density—to eliminate the influence of xylose isomerization. We explored aldehydes that included saturated aliphatic aldehydes from C1 formaldehyde to C18 octadecanal, unsaturated aliphatic C12 aldehydes ((*E*)-2-dodecenal, (*Z*)-4-dodecenal), as well as aromatic aldehyde (benzaldehyde), dialdehyde (glutaraldehyde), and ketone (acetone) (summarized in Table S16). Our aim was to explore various factors such as aldehyde size, electrophilicity, and the presence of additional functional groups

When employing HY80 zeolite as a catalyst, the di-acetalization of xylose to DAX was favored when the aldehyde was small in size (< C8). Conversely, for larger aldehydes (≥ C8), HY80 zeolite tended to favor

the mono-acetalization of xylose. This phenomenon can be explained as follows: first, the partition coefficient (C_p/C_b) of the aliphatic aldehyde concentration within the zeolite pores (C_p) compared to the bulk solution (C_b) (Figure 4d and Table S16) varied with the size of the aldehyde. Ranging from formaldehyde to octadecanal, C_p/C_b decreased from 4.0 to 0.5. Since the concentration of reactants significantly influences the reaction equilibrium, the primary product shifted from DAX to MAX when the reaction used octanal or larger aldehydes. Second, the confined environment of the zeolite pores restricts the flexibility of MAX, making it less susceptible to further acetalization. Additionally, the formation of hydronium ions and negatively charged framework aluminum tetrahedra inside the zeolite pores were found to induce a high local ionic strength, which favoured the production of more polar compounds such as MAXs with two free hydroxyl groups[31]. However, this effect appeared not pronounced in the cases where smaller aldehydes were used, likely due to the predominant influence of high aldehyde concentrations in the zeolite pores.

Another observation is that the yield of MAX-a was usually much greater than that of MAX-b, likely due to the energy difference between 3,5-*O*-dioxane acetal and 1,2-*O*-dioxolane acetal. Notably, when formaldehyde was used, the majority of monoacetals formed were in the conformation of xylopyranose with the pair of hydroxyl groups in trans positions (Figure 4d), which is likely attributed to high electrophilicity of formaldehyde stabilizing the less sterically favoured isomers. These xylopyranose monoacetals were not observed in measurable quantities with other aldehydes.

Table S16. Acetalization reaction of xylose and different aldehydes over HY80 zeolite and H_2SO_4

				**Heterogeneous yield (%)**			**Homogeneous yield (%)**		
**Entry**	**Reagent**		**C_p/C_b (25 °C, 2 h)**	**MAX-a**	**MAX-b**	**DAX**	**MAX-a**	**MAX-b**	**DAX**
1	Formaldehyde	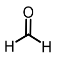	4.0	17.7	4.1	34.4	7.9	2.3	2.5
2	Propionaldehyde	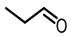	2.9	27.9 ± 0.2	0.3 ± 0.0	55.0 ± 0.3	42.3 ± 0.3	0.3 ± 0.0	16.9 ± 0.1
3	pentanal	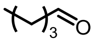	2.6	12.1	1.5	57.4	18.3	0.4	21.4
4	Trimethylacetaldehyde	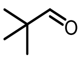	1.3	18.0	18.1	45.7	9.9	10.6	10.5
5	Octanal	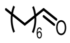	1.2	50.3 ±1.0	1.1 ±0.1	37.3 ±0.7	39.2 ±0.6	0.4 ± 0.0	24.1 ± 0.3

6	Decanal	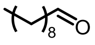	0.7	58.8 ± 2.0	2.0 ± 0.1	25.8 ± 0.1	24.5 ± 0.9	0.9 ± 0.0	47.8 ± 1.7
7	Dodecanal	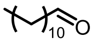	0.6	67.6 ± 1.4	5.3 ± 0.9	13.5 ± 0.6	29.2 ± 0.9	1.6 ± 0.0	28.6 ± 0.3
8	Octadecanal	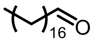	0.5	38.3 ± 1.3	5.5 ± 0.2	2.0 ± 0.1	26.3 ± 1.3	1.8 ± 0.1	18.7 ± 0.9
9	(*E*)-2-dodecenal	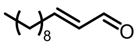	2.2	3.7	3.6	2.4	0.4	2.2	3.7
				11.4*	8.1*	12.5*	0.4*	2.3*	4.6*
10	(Z)-4-dodecenal	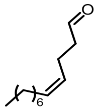	3.5	42.9	2.6	14.0	24.9	0.6	11.2
				38.3*	2.4*	25.4*	16.2*	0.9*	6.2*
11	Glutaraldehyde	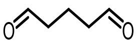	2.3	13.9		6.9	13.6		5.3
12	Benzaldehyde	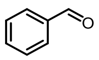	1.9	7.8	3.0	28.1	7.1	1.4	11.5
				7.5*	2.2*	55.1*	19.6*	1.4*	10.1*
13	Acetone	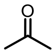	2.2	9.0	1.2	2.6	1.0	0	0.6
				1.1*	0.1*	13.3*	-	-	-

Reaction conditions: 65 °C, 5 h, 1 : 2 mol. equivalent (xylose: aldehyde), HY80 zeolite: 0.05 g·mL^{-1}. H_2SO_4: 0.02 mol·L^{-1}.
*with additional 100 g·L^{-1} 4Å molecular sieve;
Partition coefficient C_p/C_b (where C_p denotes the concentration in the zeolite pore: $C_p = \frac{C_{initial}\cdot V - C_b\cdot V}{V_{pore}}$, C_b stands for the bulk concentration after adsorption, and v_{pore} is the pore volume as measured by N_2 adsorption).
Error margins represent standard deviations from three independent experiments.

Table S17. Xylose acetalization with formaldehyde using $AlCl_3$

	**Xylose conversion (%)**	**Diacetal yield (%)**
Ambient conditions (no drying procedure)	100	60.4
Dry conditions	96.1	2.0

Although NaY5 zeolite can be used to elucidate the lack of catalytic activity associated with weak Lewis acid sites (Figure 4b), the role of a stronger Lewis site requires further investigation, especially considering that most zeolites used in this work may contain strong Lewis sites (Figure S21). To this end, $AlCl_3$ was used as to understand the effect of strong Lewis acids in catalyzing xylose acetalization with formaldehyde (Table S17). Since $AlCl_3$ can rapidly release HCl, a strong Brønsted acid, upon exposure to trace moisture, it was impractical to eliminate any Brønsted acid in the reaction system. Instead, a pair of comparison experiments were conducted with varying moisture levels: the first was run under ambient conditions using regular solvents, and the other using dehydrated solvent in a sealed reactor prepared in a glovebox. By decreasing the moisture content, the product yield sharply decreased, which suggested that $AlCl_3$ alone was not sufficient to catalyze xylose acetalization despite being stronger than the Lewis acidity of NaY5. These findings support the conclusion that Brønsted acid sites in zeolites are the dominant active species in this reaction system. The high xylose conversion in both of these control experiments may be attributed to $AlCl_3$ catalyzing side reactions such as xylose isomerization and subsequent degradation, as evidenced by the dark coloration of the reaction mixture.

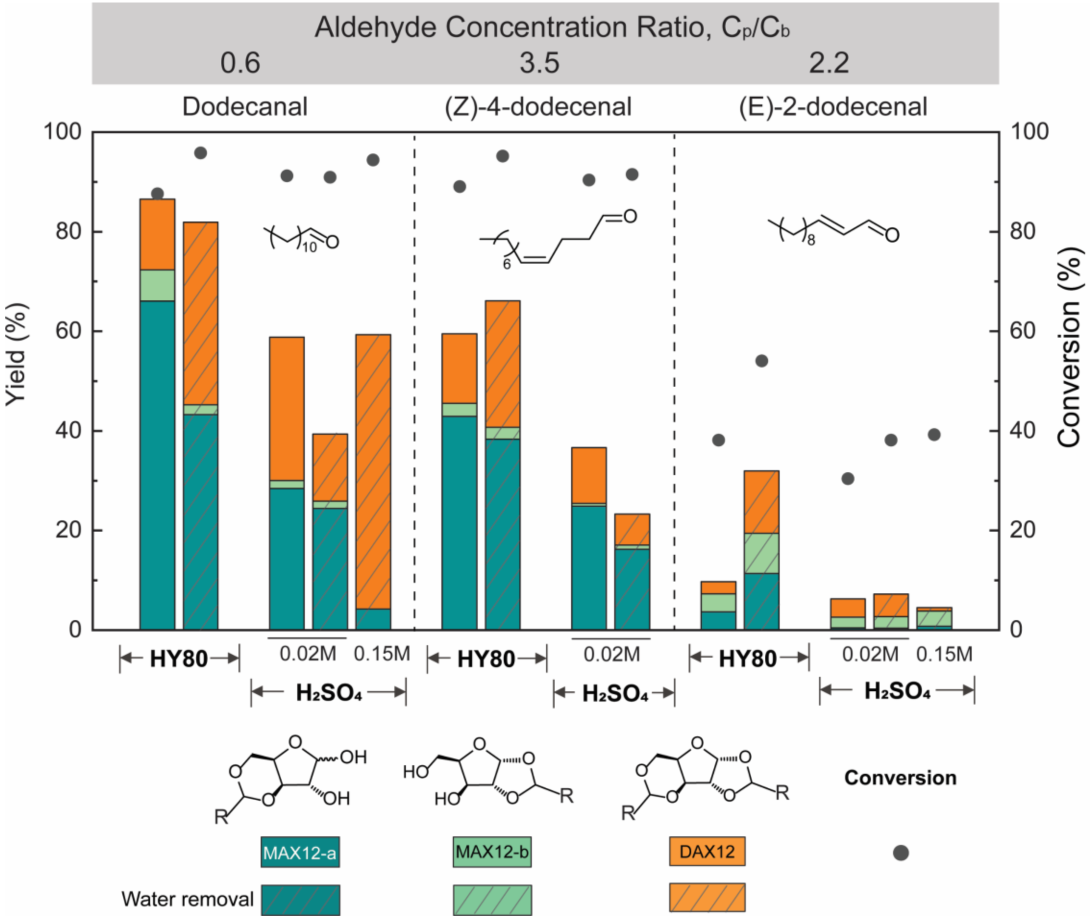

Figure S33. Acetalization reaction of xylose and C12 aldehydes with different electrophilicity over HY80 zeolite and H_2SO_4: saturated aliphatic aldehyde versus unsaturated aliphatic aldehyde with double bond at position 2 and 4. Water removal is achieved by introducing 4 Å molecular sieve.

Maintaining the same aldehyde chain length, we explored the impact of the presence of double bonds on aldehyde molecules, specifically comparing (*E*)-2-dodecenal and (*Z*)-4-dodecenal. The presence of a double bond tends to diminish the electrophilic nature of the aldehyde, and this effect is particularly pronounced in the case of (*E*)-2-dodecenal, where the double bond is located at the α position. Consequently, the yields of desired products exhibit a significant decrease, with the highest total yield reaching a mere 7.3% when employing 0.02 M H_2SO_4 with water removal. Remarkably, we observed an improvement in yields when using HY80 (with water removal) as a catalyst compared with H_2SO_4, although the overall yield still remains relatively modest at 32%. This enhancement can be attributed to the π-interactions between unsaturated aldehydes and Lewis acid sites within the catalyst. These interactions lead to greater adsorption of aldehydes within the porous structure (C_p/C_b=2.2) of the catalyst, resulting in increased reactant concentrations and a shift in the equilibrium toward the products. It's noteworthy that this π-interaction phenomenon has practical applications, such as the separation of olefins from paraffins[33][34]. As an

illustrative example, benzaldehyde, which possesses a C_p/C_b ratio of 1.9, led to a DAX yield of 55% with HY80, whereas it only led to yields around 10% with H_2SO_4.

Additionally, we discovered that the removal of water from the reaction mixture enhances DAX selectivity when utilizing zeolites as catalysts. In contrast, when H_2SO_4 is employed as the catalyst, the introduction of 4Å molecular sieve has an adverse effect on the mole balance. This may be attributed to unidentified side reactions catalyzed by leached metal ions. Exposure of the molecular sieve to strong acid could result in the dissolution of metal ions contained within, altering its surface properties and affecting its water adsorption capabilities.

7.4 Solvent screening

We also explored the use of various environmentally friendly solvents, such as CPME, 2MeTHF, and GVL, as reaction media. We observed a strong dependency of the reaction rate on the solubility of xylose in these solvents. In general, at the onset of the reaction, xylose may not fully dissolve initially due to its high hydrophilicity. As the reaction progresses, xylose gradually dissolves. If the solubility of xylose in a particular solvent is too low, resulting in a low concentration of available reactants, the thermodynamics of the reaction favor the reactants, and the reaction rate may be low due to the kinetics. This phenomenon explains the relatively low reaction conversion observed in solvents like CPME, 2MeTHF, and THF with the same reaction conditions. In contrast, GVL shows slightly higher solubility for xylose when compared to 1,4-dioxane. As a result, under identical reaction conditions, we observed comparable conversion rates but a greater yield of DAX12 with GVL compared to 1,4-dioxane.

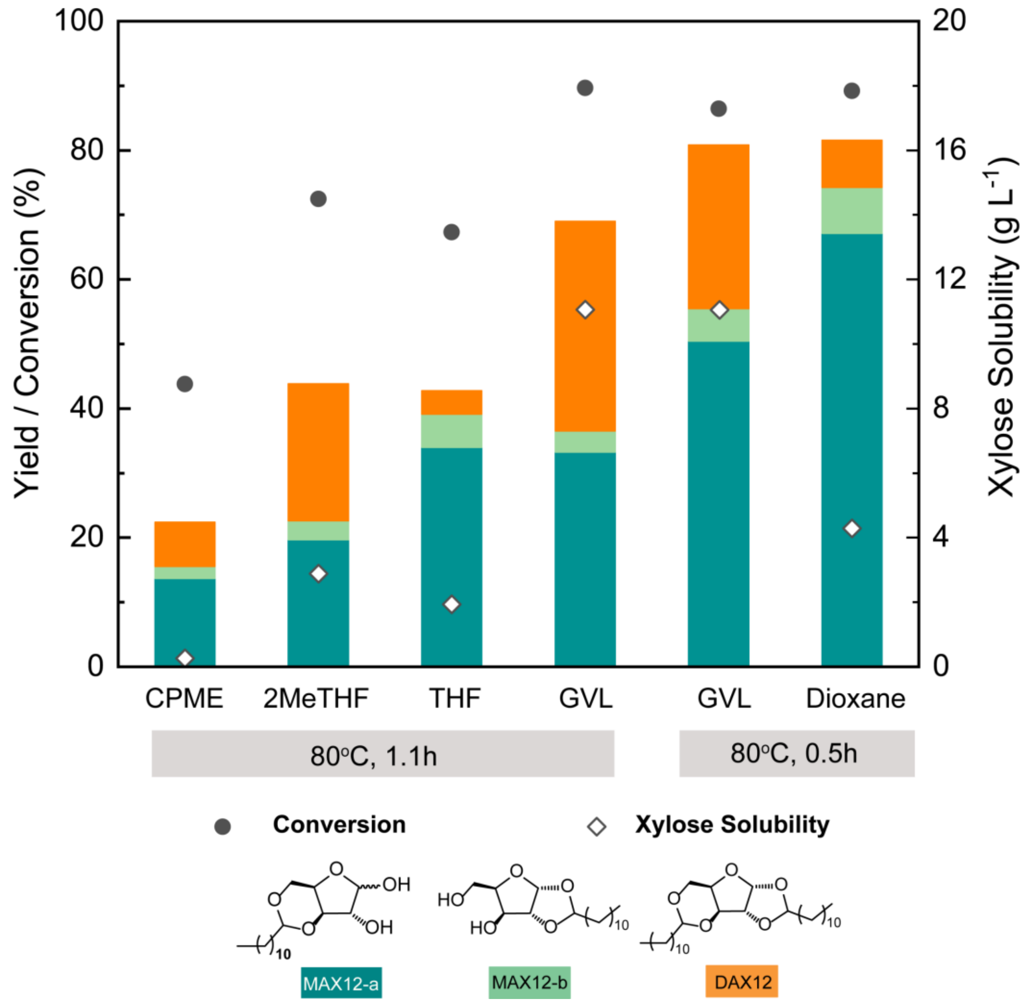

Figure S34. Reaction solvents screening. Xylose acetalization reaction yield/conversion and xylose solubility (tested at 80 °C) in different solvents.

Reaction conditions: 80 °C, 1 : 2 mol. equivalent (xylose : dodecanal).

7.5 Acetalization reaction of other pentoses

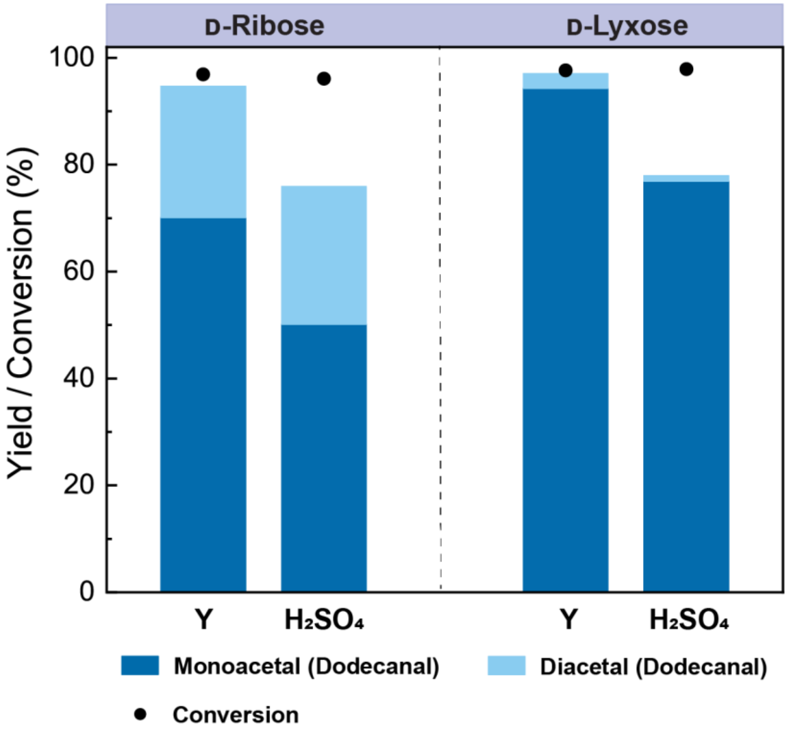

Figure S35. Acetalization of D-ribose and D-lyxose with dodecanal over HY80 zeolite and H_2SO_4.

Reaction conditions: 65 °C, 5 h, 1:2 mol. equivalent (sugar : dodecanal), zeolite: 0.05 g · mL^{-1}, H_2SO_4: 0.02 mol · L^{-1}.

In the acetalization of D-ribose and D-lyxose with dodecanal, there was no significant difference in selectivity between the HY80 and H_2SO_4 catalysts. Both primarily yield monoacetals as the main product, especially when D-lyxose is used as the substrate. This preference for monoacetal formation, even without confinement, can be attributed to the inherent high ring-strain in the trans configuration during the formation of a second cyclic acetal, or the significant steric hindrance associated with the formation of a "boat-like" tricyclic fused ring (Figure S1). In such instances, pore confinement is considered unnecessary for the production of monoacetals.

8. Performance of xylose monoacetal surfactants (MAXn, GMAX, MAXS)

8.1 Amphiphilic properties test

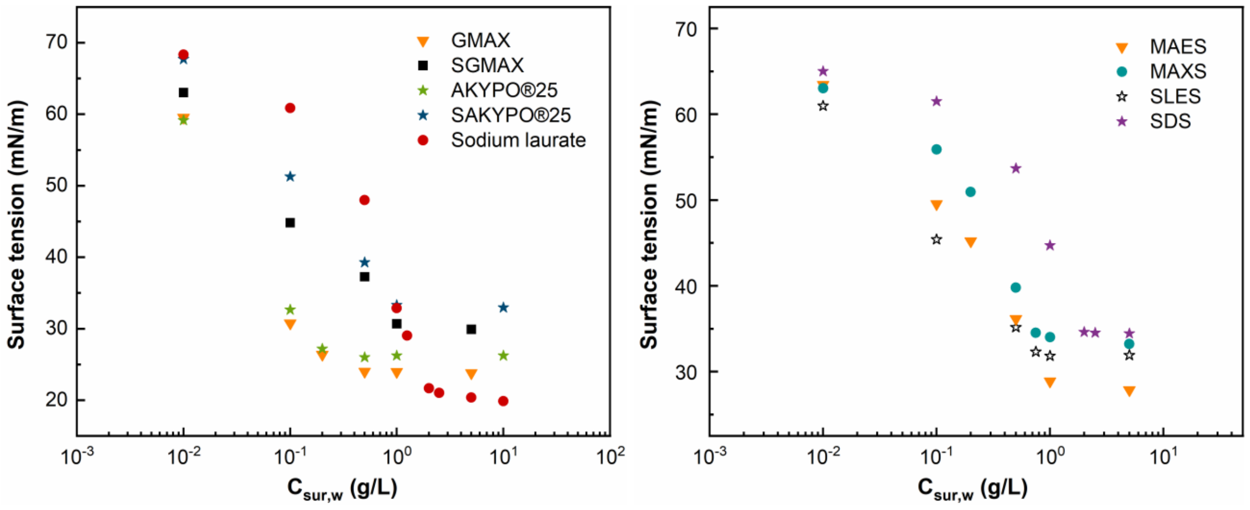

Figure S36. Surface tension of water with different concentrations of GMAX, SGMAX, MAXS, MAES and compared with the commercial anionic surfactants possessing the same ionic groups (acronyms prefixed with 'S' designates sodium carboxylates).

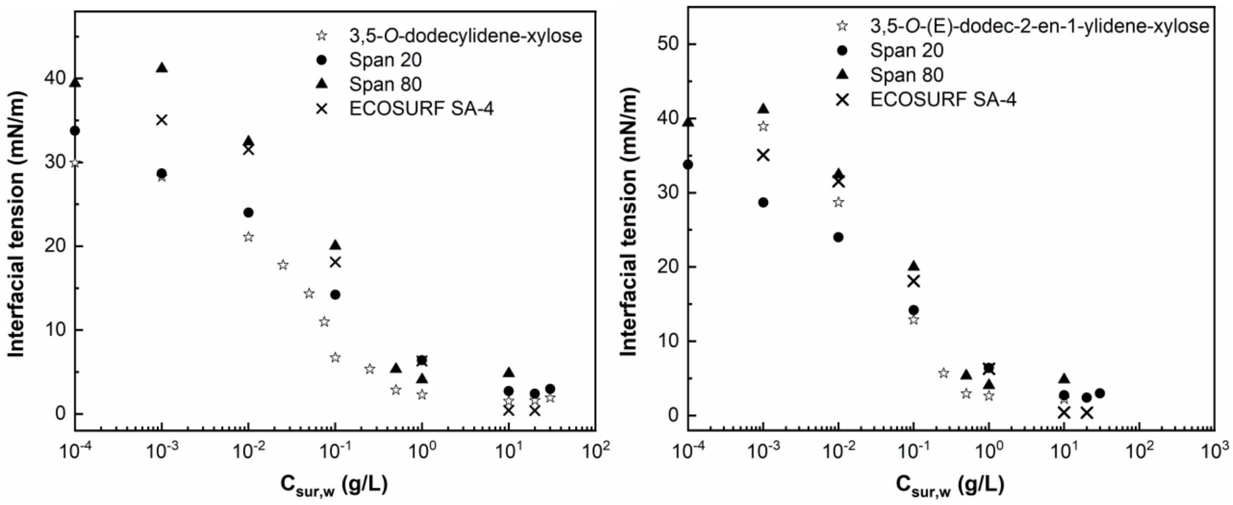

Figure S37. Interfacial tension of cyclohexane-water (50.2mN/m) interface at different concentrations of 3,5-O-dodecylidene-xylose (MAX12), 3,5-O-(*E*)-dodec-2-en-1-ylidene-xylose (MAX12:1(2)), and some of the most common commercial surfactants.

8.2 pH-responsive GMAX

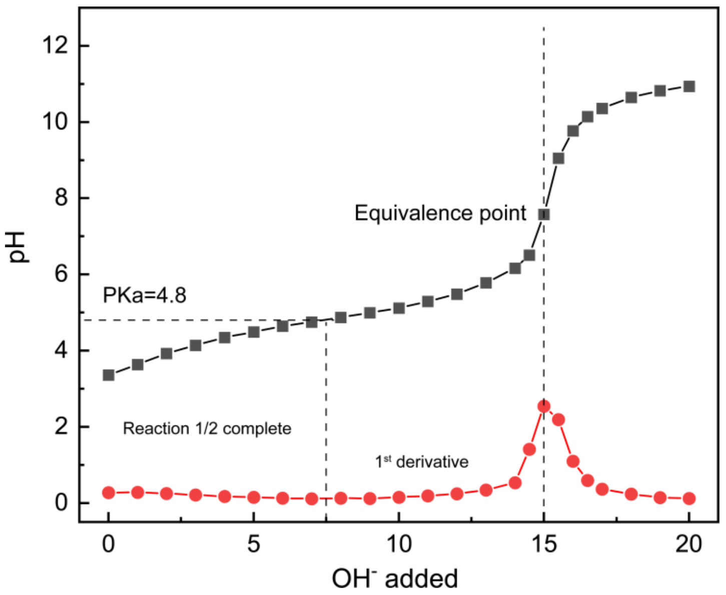

Figure S38. pKa measurement of GMAX through acid-base titration experiment.

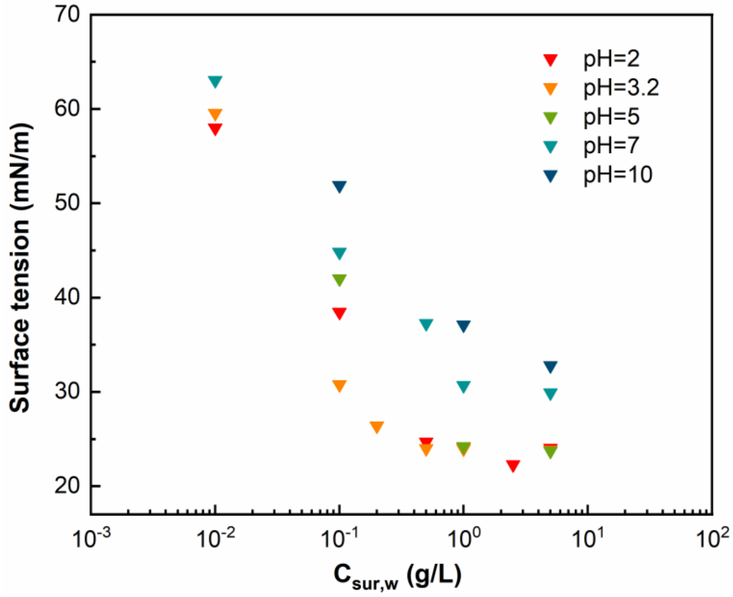

Figure S39. Surface tension of water with different concentrations of GMAX at different pH.

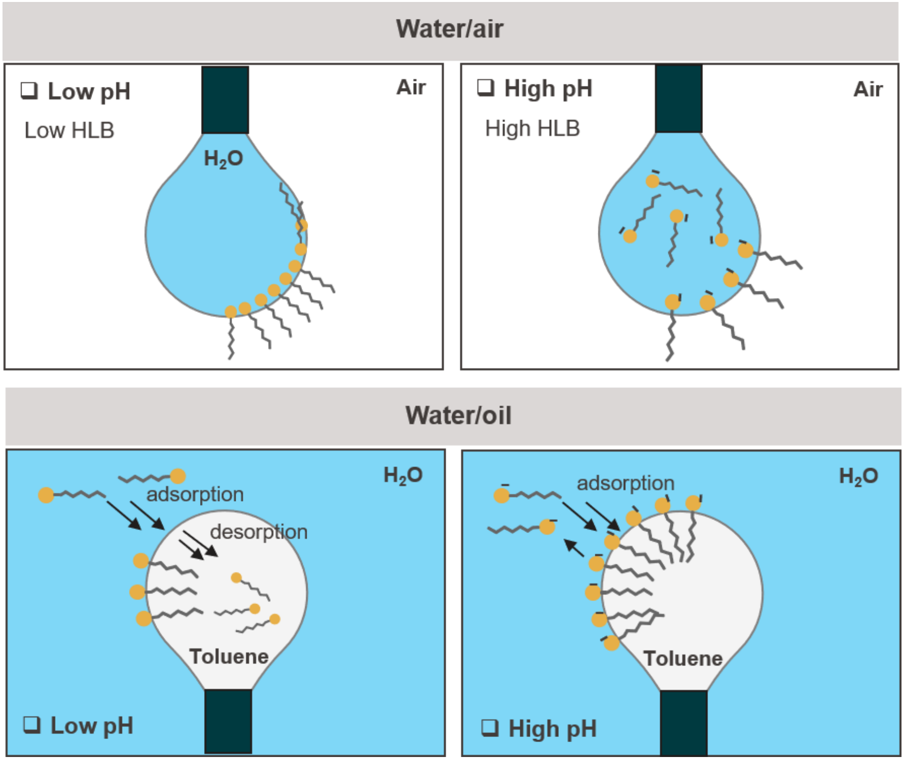

Figure S40. Schematic diagram of pH responsive property of GMAX in the water/air and water/oil system.

Additional discussion:

Reduction in ST/IFT by amphiphilic substances is influenced by several factors, including the density of surface-active molecules adsorbed at the interface and the area occupied by each molecule on the interface[35]. When one part of a molecule prefers the polar phase, while the other part prefers the nonpolar phase, there is a higher tendency for these molecules to accumulate at the interface. At low pH, GMAX is considered as “nonionic”, exhibiting a preference for the nonpolar phase. Therefore, it tends to accumulate on the water surface in the test of ST, or favors to diffuse to the nonpolar liquid phase in the test of IFT; Conversely, at high pH, GMAX is deemed “ionic”, possessing much improved hydrophilicity. Consequently, it doesn’t remain confined to the water surface in the test of ST, and it tends to accumulate at the interface for its balanced hydrophilic and hydrophobic moieties in the test of IFT.

8.3 Emulsion polymerization with GMAX and SDS

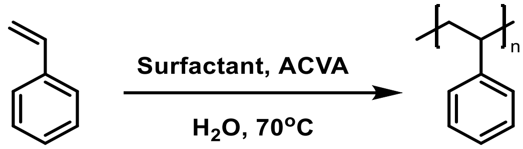

We utilized GMAX in the application of emulsion polymerization, and compared it with SDS, a commonly used surfactant for this application, following the protocol published by Molina-Gutiérrez et al.[36]. The emulsion polymerization was carried out with thermal initiator using 4,4'-azobis (4-cyanopentanoic acid) (ACVA) at 70 °C. The surfactant solution (SDS or GMAX, 4 wt. % based on monomer) was prepared with $NaHCO_3$ (0.14 mmol) to adjust the pH. Meanwhile, the initiator ACVA (0.07 mmol) solution was also prepared right before usage. All the water solution and styrene monomer were purged under N_2 for 30 min. Then the initiator and the monomer (9.6 mmol) were transferred to the surfactant solution using a syringe to afford a latex at 10-12 wt % solids content. After this, the reactor was heated to 70 °C. Monomer conversion was followed through ^{1}H NMR using $CDCl_3$ as the deuterated solvent.

Table S18. Emulsion polymerization of styrene using GMAX or SDS

Surfactants	Monomer conversion (%) by NMR	M_n (6 h)	M_w (6 h)	*Đ*	Particle diameter (nm)/ PdI	Zeta potential
GMAX	76% (5h)	403 000	824 000	2.04	68.0/0.031	-78.8 mV
SDS	80% (5h)	350 000	766 000	2.18	74.8/0.081	-87.1 mV

Table S19. Particle size of the styrene latex and its stability

	Particle diameter (nm)			PdI		
	SDS-9.1	GMAX-9.1	GMAX-9.5	SDS-9.1	GMAX-9.1	GMAX-9.5
Day 0	74.8	68.0	67.2	0.081	0.031	0.038
Day 7	72.8	68.8	76.7	0.122	0.058	0.192
Day 14	69.2	70.2	74.5	0.148	0.059	0.099
Day 28	78.0	70.0	75.3	0.128	0.028	0.105

8.4 Resistance to hard water

Hard water contains dissolved salts, commonly Mg^{2+} and Ca^{2+} ions, which interact with surfactants and decrease their detergency efficiency[37]. Table S20 listed the classification for hard and soft water according to the United States Geological Survey[38]. The hardness level of 50,000 ppm tested in this work represents an extreme condition, surpassing even the hardness of seawater, which typically measures around 5800-7500 ppm.

Table S20. Water hardness scale

Concentration as $CaCO_3$ (ppm)	Classification
0-60	Soft water
60-120	Moderately hard water
120-180	Hard water
>180	Very hard water
5800-7500	Seawater[39]

Recognized hard water-resistant surfactants, exemplified by SLES and AKYPO, share a pivotal structural feature—the incorporation of a polyethylene oxide (PEO) segment. This PEO segment has the capacity to form weak complexes with Mg^{2+} and Ca^{2+} ions[40], which weakens the combination between Mg^{2+}/Ca^{2+} ions and sulfates/carboxylates[41],[42]. Additionally, the presence of PEO significantly increases the solubility of the surfactant (*e.g.*, surfactanat calcium salt). This dual effect reduces the tendency for precipitation when encountered with counter ions under hard water conditions[43]. Besides, recently published surfactants known for their high hard water resistance, such as oleo-furansulfonate (OFS)[44] and sulfonated alkyl furoates (SAF)[45], possess a furan ring. This structural feature is presumed to contribute to a chelating effect.

In this work, the insertion of a sugar core likely functions similarly to the PEO moiety by enhancing the water solubility of surfactants, and providing chelation sites for metal cations. SGMAX demonstrates improved hard water tolerance compared to sodium laurate (Figure S41-43), primarily due to its enhanced water solubility. However, SGMAX's resistance to hard water is lower than that of polyoxyethylene alkyl ether carboxylic acids (AEC), likely due to the difficulty of chelating metal cations caused by the rigidity of its tricyclic fused ring structure, as opposed to the flexibility of PEO. In contrast, MAXS, with its free hydroxyl group within the sugar core, offers a more flexible structure for chelating metal cations. This flexibility allows MAXS to exhibit robust hard water stability, comparable to that of SLES.

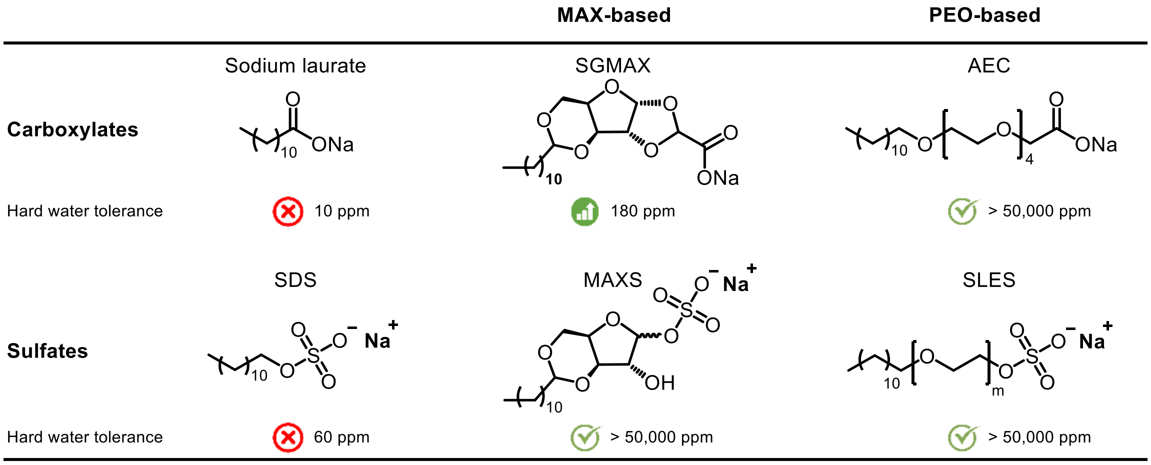

Figure S41. Hard water tolerance of carboxylate/sulfate surfactants compared to those with xylose sugar core or PEO moiety insertion. The values of hard water tolerance above are obtained from Figure S42.

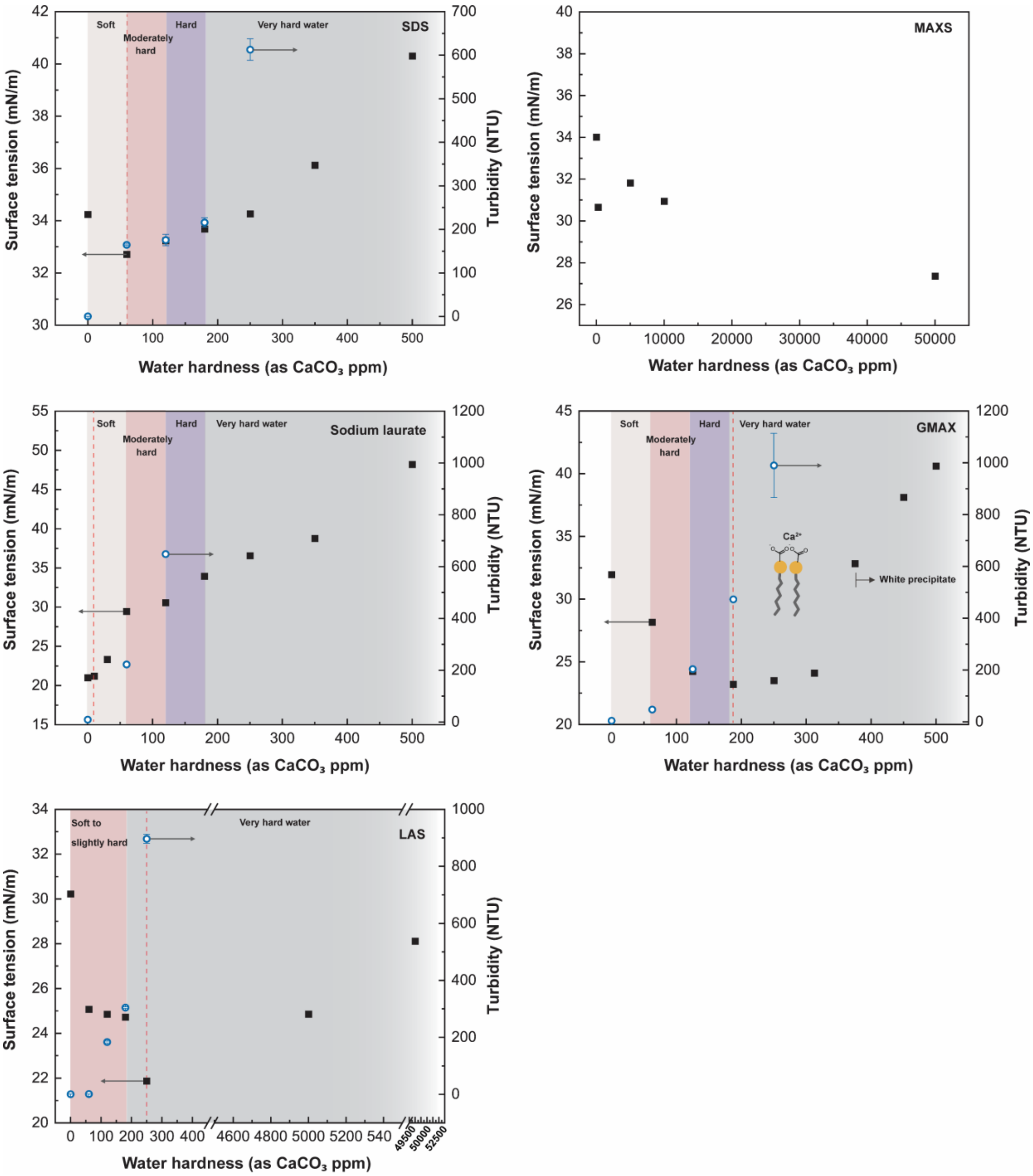

Figure S42. Surface tension vs. $CaCl_2$ concentration of the SGMAX, MAXS and commercial LAS, SDS surfactants (Concentration of the surfactant: 1.5-2 times of CMC, Micelle stability concentration: calcium concentration at the increasing point of the surface tension indicated by the red dashed line).

Standard hard water samples were prepared by dissolving calculated amount of $CaCl_2$, and expressed in terms of equivalent hardness of $CaCO_3$. The turbidity created by the introduction of hard water was determined by a Turbidimeter with a testing range of 0-1000 NTU. The turbidity test typically measures up to 250 ppm water hardness due to exceeding the detection limit (1000 NTU) beyond this level. Sodium laurate detection ends at 120 ppm due to exceeding the detection limit beyond this threshold.

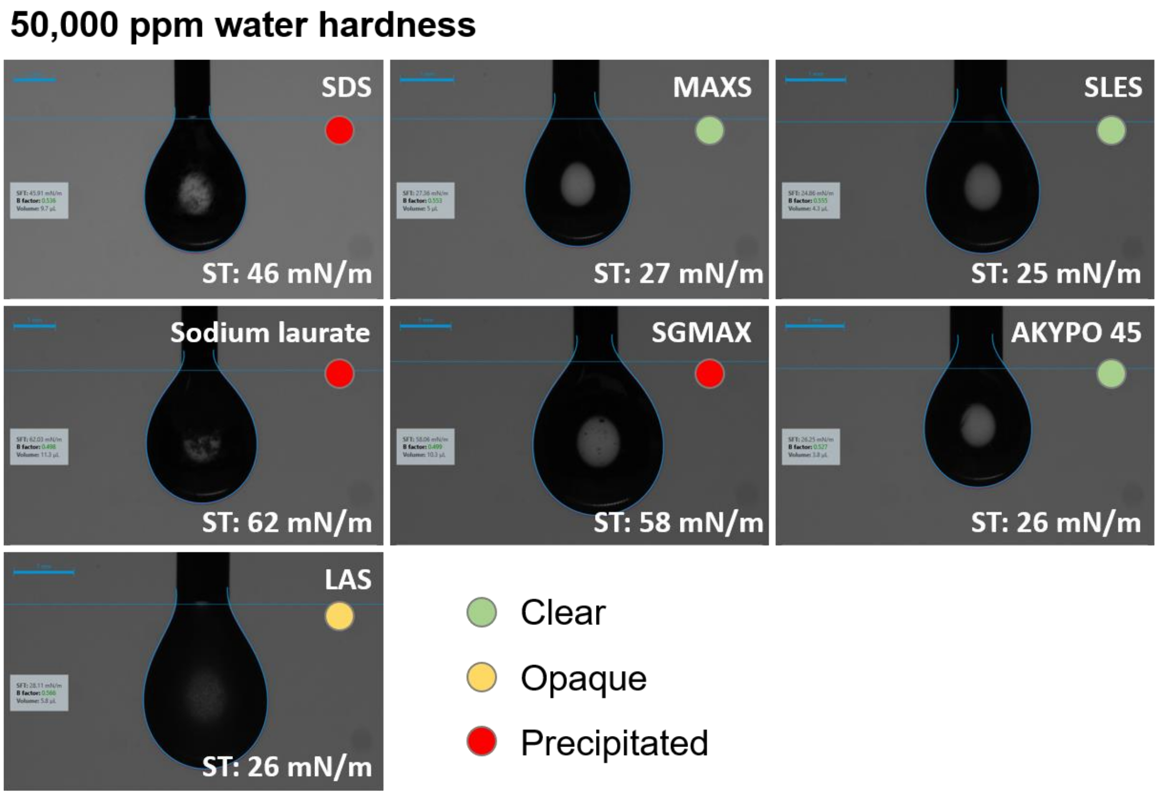

Figure S43. The ST of different surfactants exposed to 50,000 ppm hard water.

8.5 Emulsion stability test

8.5.1 MAX12

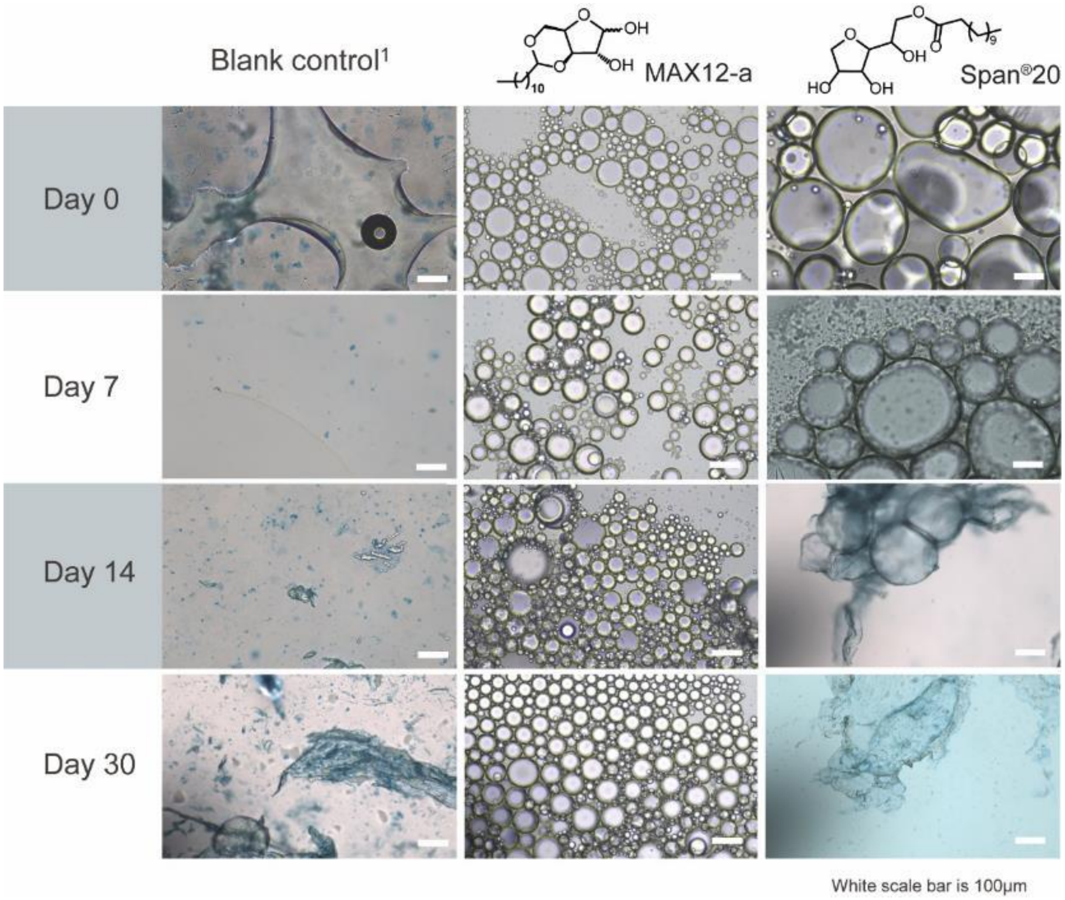

Figure S44. Optical microscopy images of water-in-oil emulsions (2 mL cyclohexane with 1 mg·mL^{-1} surfactant + 1 mL water containing 1 mg·mL^{-1} Alcian blue) at days 0, 7, 14, and 30.

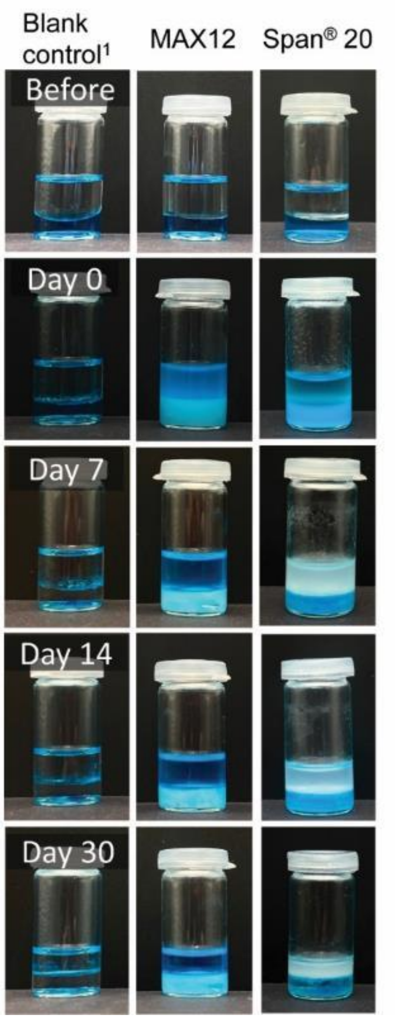

Figure S45. The visual appearance of water-in-oil emulsions stored at 25 °C at 0, 7, 14, and 30 days. 2 mL cyclohexane with 1 mg·mL^{-1} surfactant + 1 mL water containing 1mg·mL^{-1} alcian blue.

[1]Blank control: 2 mL cyclohexane + 1 mL water containing 1 mg·mL^{-1} alcian blue dye.

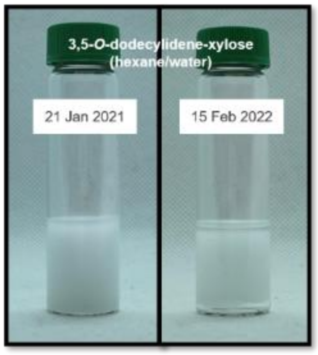

Figure S46. Images of water/hexane emulsions (10 mg·mL^{-1}) taken after 390 days.

8.5.2 GMAX

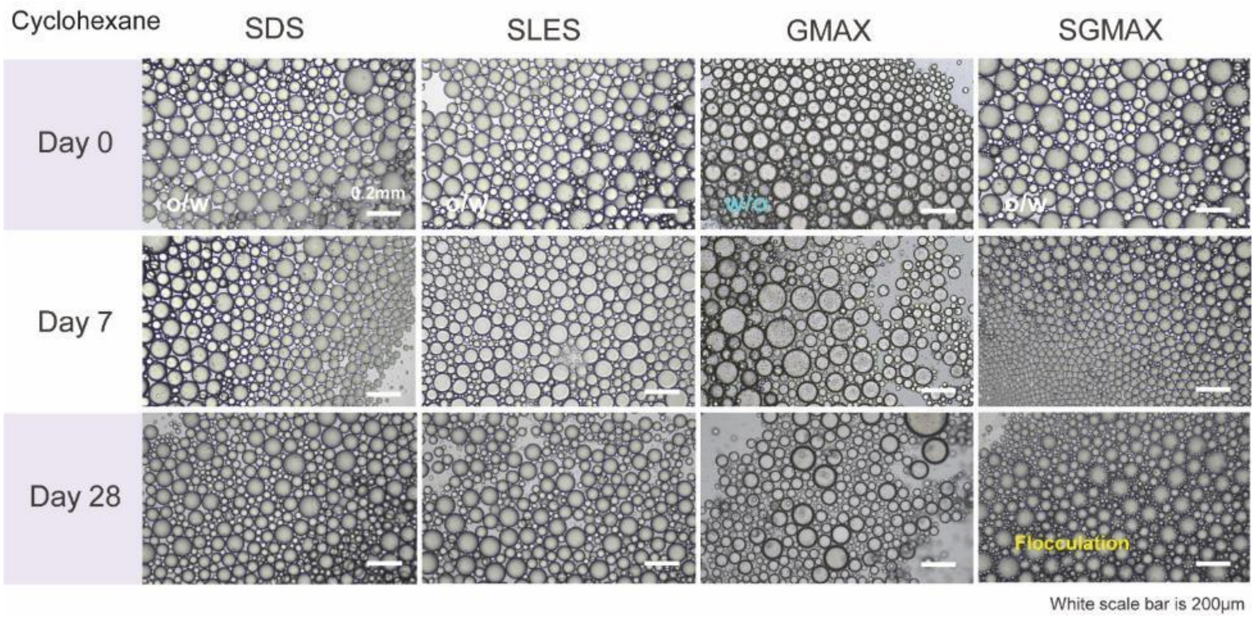

Figure S47. The optical microscopy images of emulsions (1 mL cyclohexane with 0.05 mg·mL^{-1} Sudan black B + 1 mL water containing 5 mg·mL^{-1} surfactant) at days 0, 7, and 28.

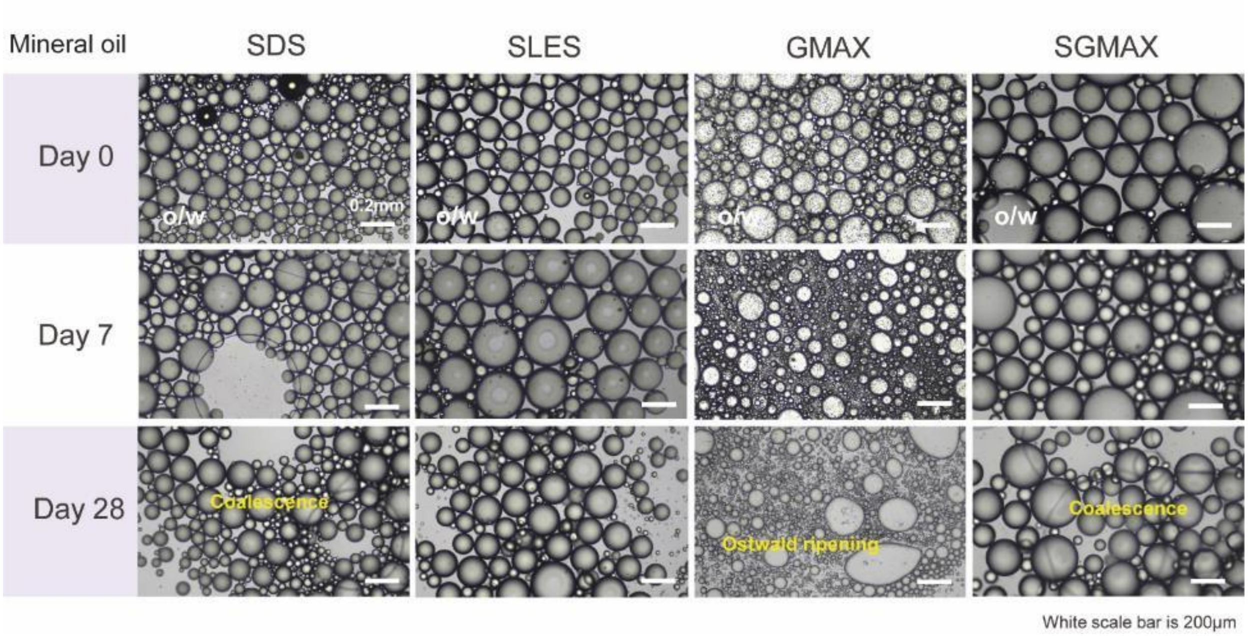

Figure S48. The optical microscopy images of emulsions (1 mL mineral oil with 0.05 mg·mL^{-1} Sudan black B + 1 mL water containing 5 mg·mL^{-1} surfactant) at days 0, 7, and 28.

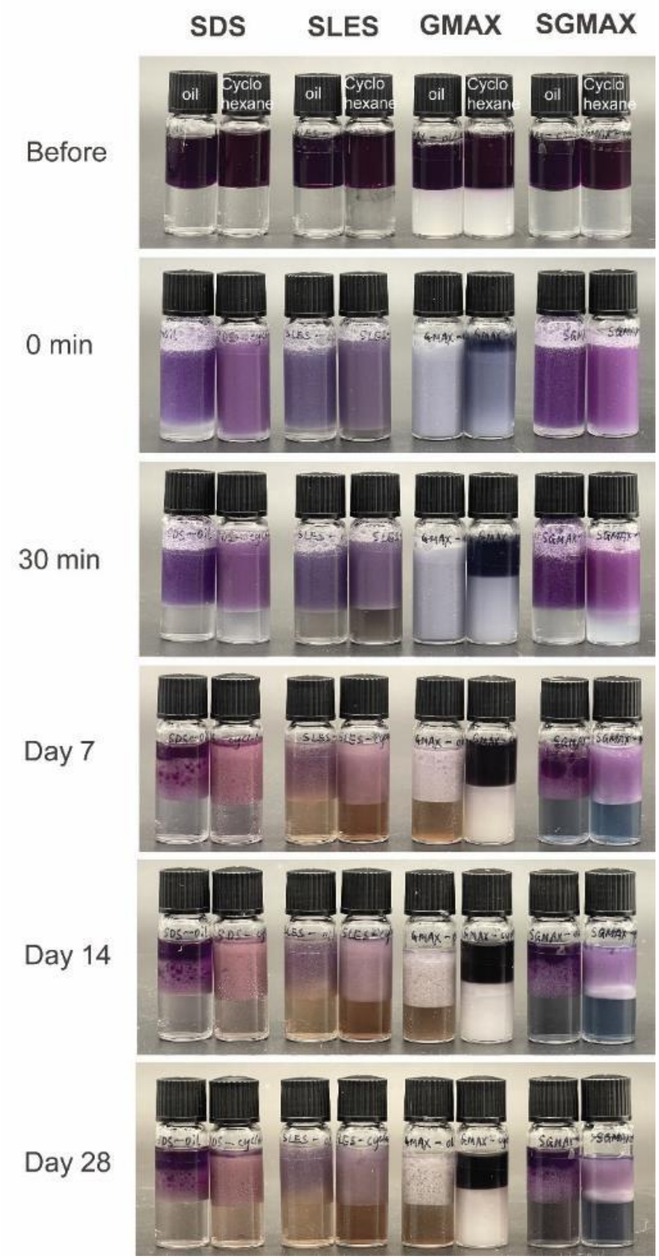

Figure S49. The visual appearance of emulsions (1 mL mineral oil with 0.05 mg·mL^{-1} Sudan black B + 1 mL water containing 5 mg·mL^{-1} surfactant) at days 0, 7, and 28.

8.5.3 MAXS

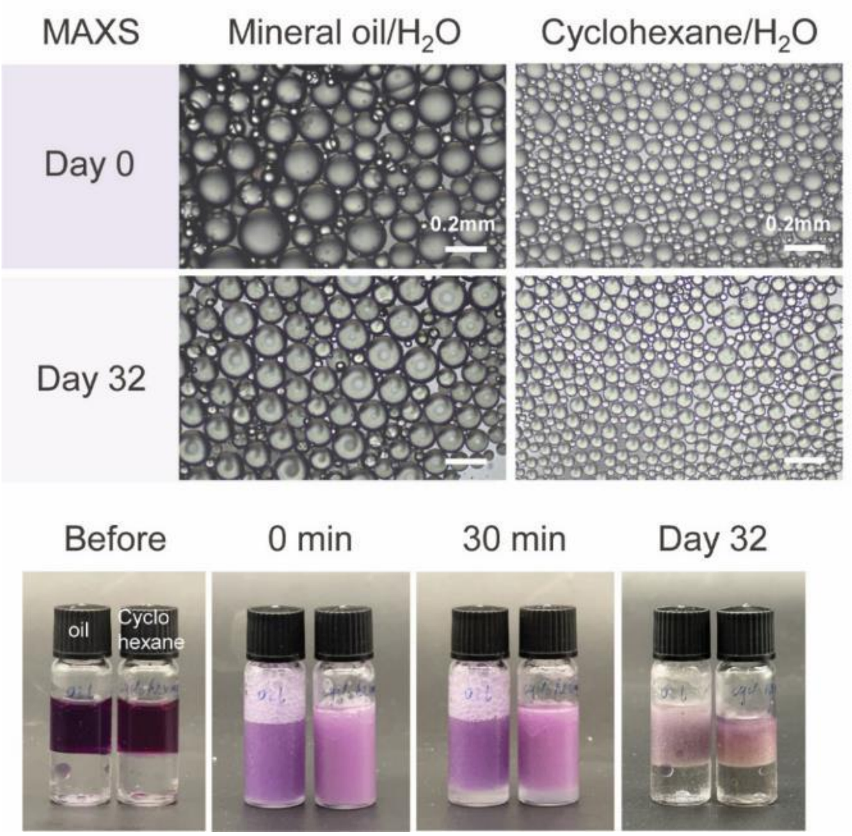

Figure S50. The optical microscopy images and the visual appearance of emulsions (1 mL mineral oil with 0.05 $mg{\cdot}mL^{-1}$ Sudan black B + 1 mL water containing 5 $mg{\cdot}mL^{-1}$ MAXS surfactant) at days 0, and 32.

8.6 Foaming property

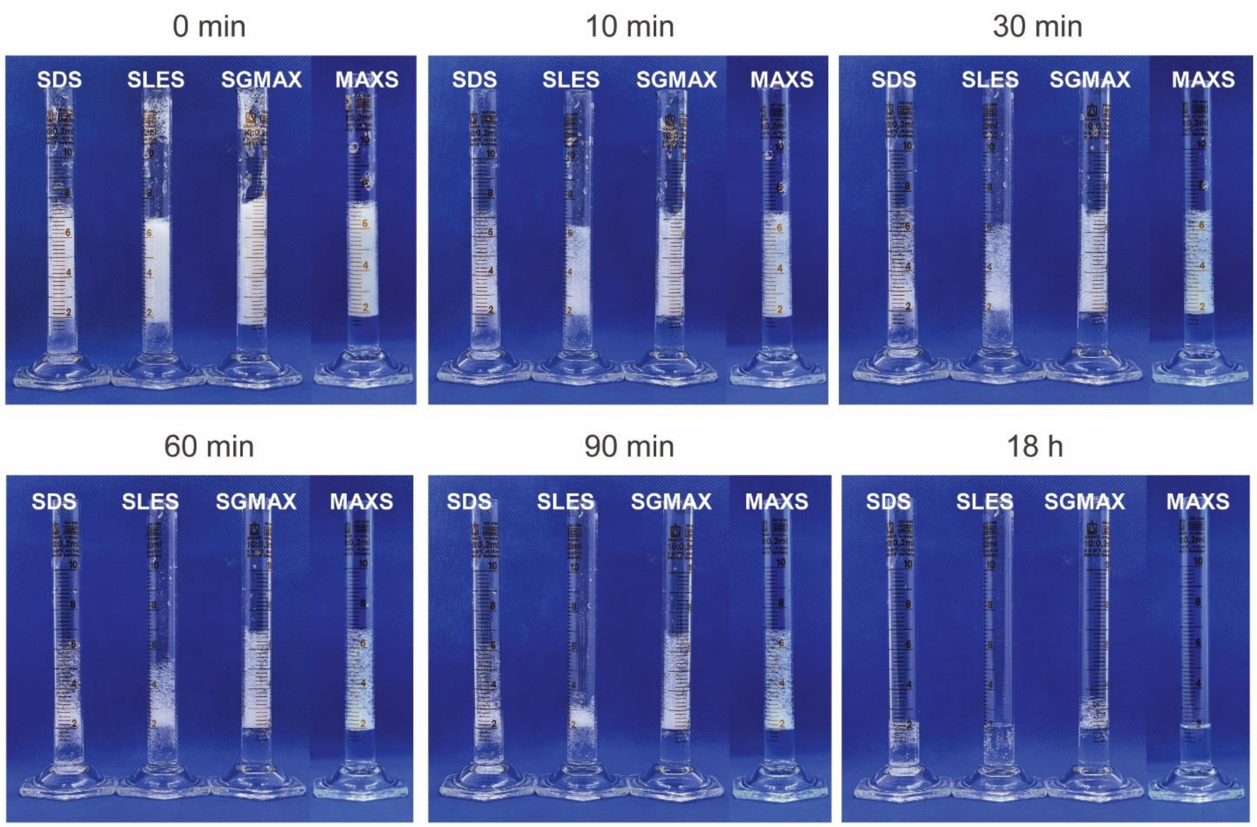

Figure S51. Foaming property of SDS, SLES, SGMAX and MAXS surfactants at 5 mg·mL^{-1}

8.7 Krafft temperature

The Krafft point of the surfactants was determined by assessing the extent of counterion dissociation using a conductivity meter. The measurement process involved gradually heating the sample from 0 °C while measuring conductivity at every 1 °C increment until a steady value was reached. The Krafft point was determined as the temperature at which the conductivity vs. temperature graph displayed a significant change in slope. To clearly observe the inflection point, the first derivative of conductivity with respect to temperature was calculated. For MAES, the Krafft point was determined to be 26 °C. Below 26 °C, the surfactants exist in a crystalline form, while between 26 °C and 37 °C, both crystalline and micellar forms coexist. Above 37 °C, all surfactant molecules are fully dissolved, forming micelles. This transition was visually observed as a shift from a cloudy state (indicating the presence of precipitated surfactant crystals below the Krafft point) to a clear solution (indicating the dissolution of surfactants and the formation of micelles in water). The SGMAX and MAXS surfactant solutions remained clear even at 0 °C, and no inflection point was observed in the conductivity vs. temperature curve. Therefore, we concluded that their Krafft points were below 0 °C.

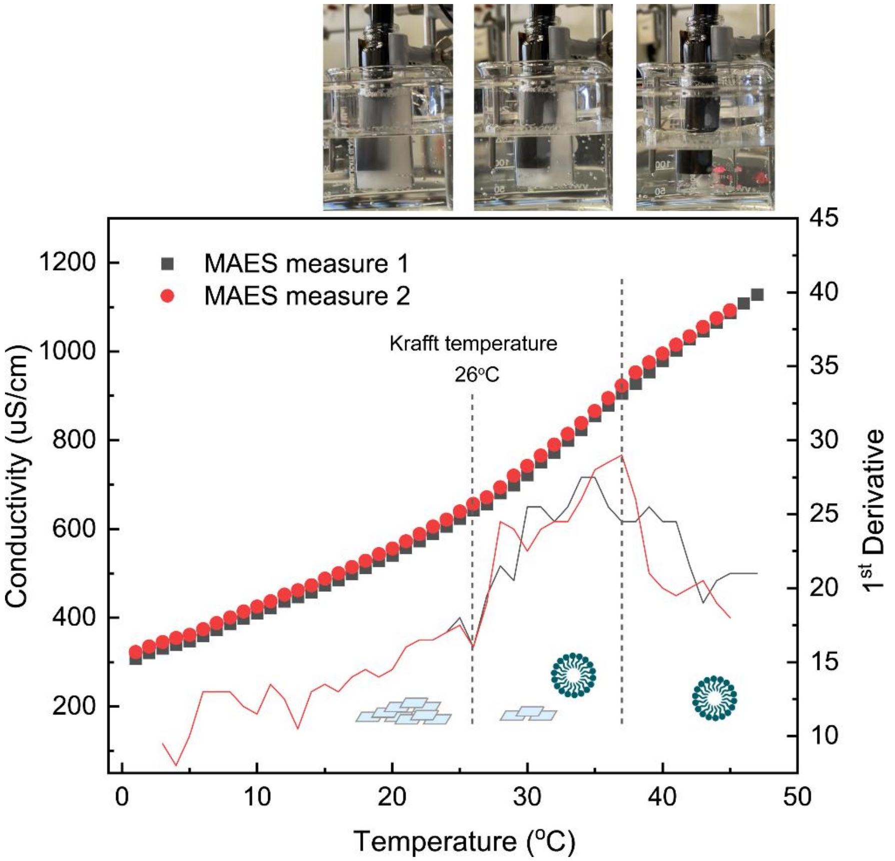

Figure S52. Conductivity versus temperature of 0.25 wt% surfactant solutions for determination of Krafft point for MAES.

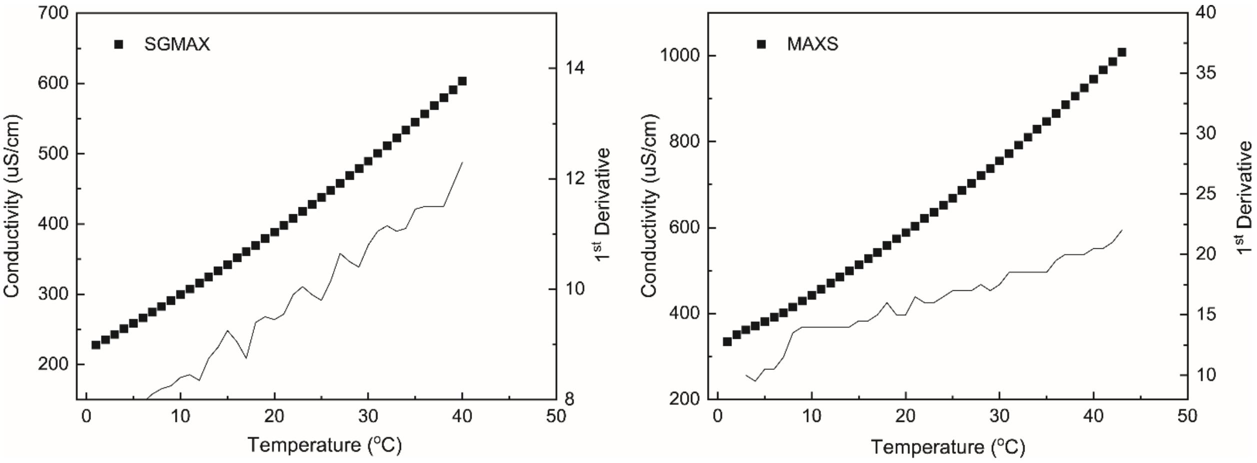

Figure S53. Conductivity versus temperature of 0.5 wt% surfactant solutions for determination of Krafft point for SGMAX and MAXS.

8.8 Washing test

A spectrophotometer is utilized to numerically assess the cleaning efficiency using the L, a, b parameters on a color chart. Each fabric sample is measured three times, and the average value is used.

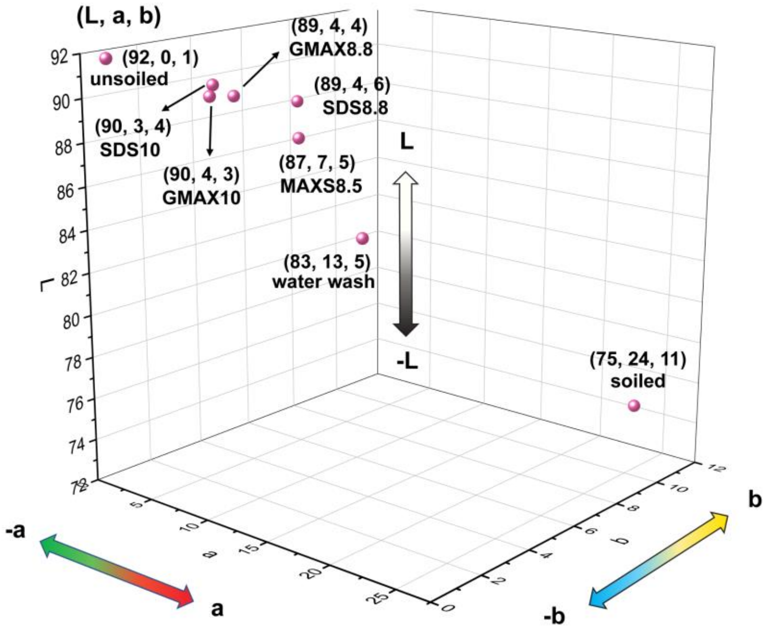

Figure S54. L, a, and b color chart of unsoiled and soiled fabric with lipstick (standard stained cotton fabrics CS116), and washed soiled fabric with different surfactants under different pH (the surfactant names are suffixed with the pH). (The letters L, a and b represent each of the three values the CIELAB color space uses to measure objective color and calculate color differences. L represents lightness from black to white on a scale of 0 to 100, while a and b represent chromaticity with no specific numeric limits).

The cleaning efficiency of the colored stain is defined as the distance between the washed-and-soiled divided by the distance of the unsoilded-and-soiled:

$$\sqrt{(L_w - L_s)^2+(a_w - a_s)^2+(b_w - b_s)^2} \,/\, \sqrt{(L_u - L_s)^2+(a_u - a_s)^2+(b_u - b_s)^2} \times 100\%$$

The cleaning efficiency of black stain is defined as the whiteness of the washed fabric divided by the whiteness of unsoiled fabric:

$$L_w \,/\, L_u \times 100\%$$

Subscript "w" represent washed; "s" indicates soiled; u means unsoiled.

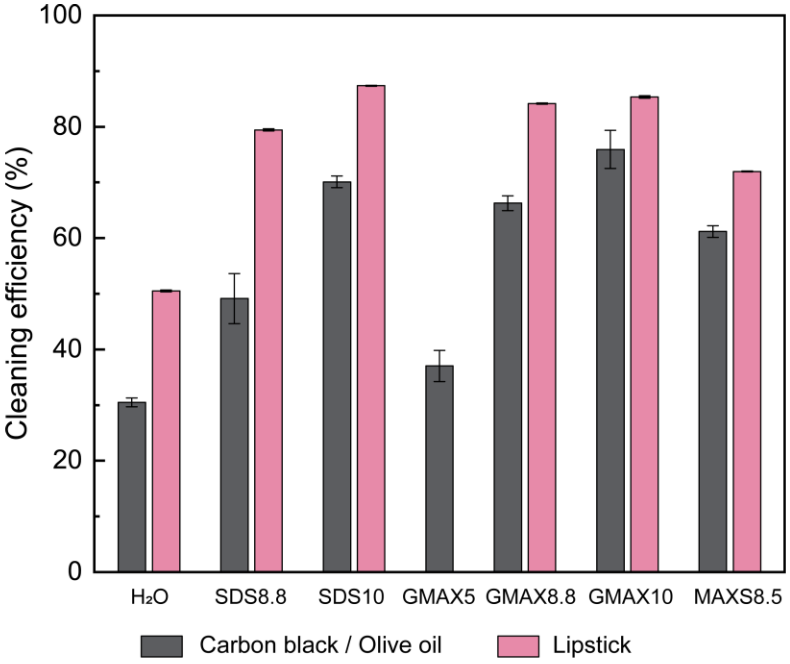

Figure S55. The cleaning efficiency of SDS, GMAX, and MAXS (suffixed with the pH) for removing different stains (standard stained cotton fabrics E-101, CS116) by ultrasonic cleaner.

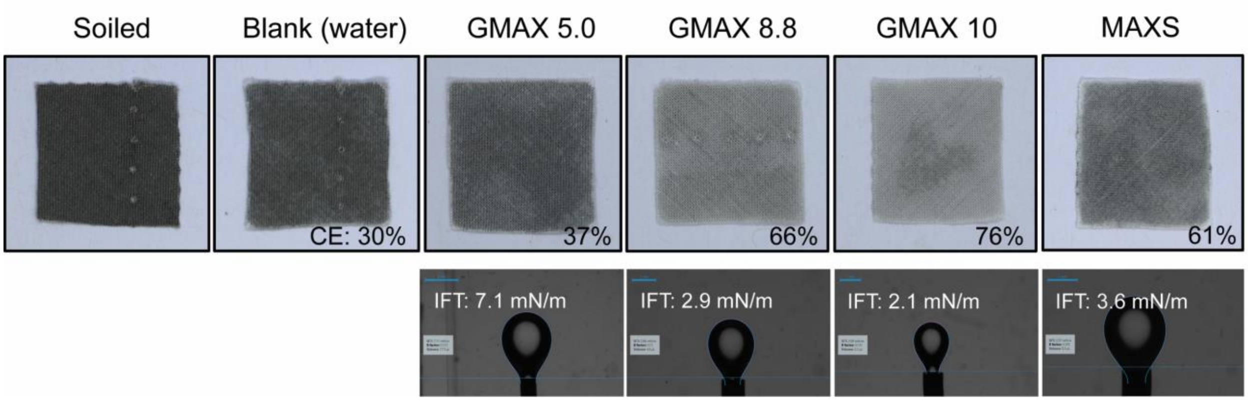

Figure S56. The cleaning efficiencies of GMAX (pH=5, 8.8, 10) and MAXS are inversely correlated to their IFT (toluene/water).

8.9 End-of-life properties of MAX12

8.9.1 Accelerated aqueous decomposition of MAX12

To gain insight into the degradation products of MAX12 in water, an accelerating aqueous decomposition test was performed by boiling it in water. Samples were taken at various time points and analyzed by HPLC (pH2 aqueous-phase chrmatography, method see S2.5). It shows that MAX12 can be cleaved into xylose and fatty aldehyde in boiling water in 2 days. And to our knowledge, fatty aldehydes can be oxidized into fatty acids catalyzed by the aldehyde dehydrogenase enzyme. Xylose and fatty acids are readily biodegradable. The result shows that MAX12 is unlikely to persist in the environment and cause long-term pollution.

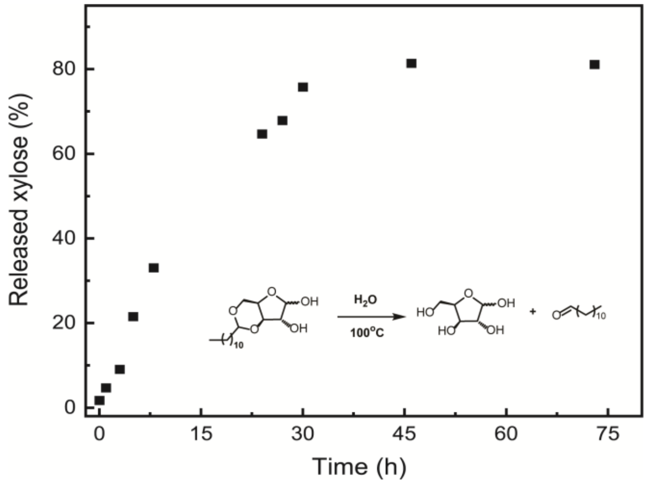

Figure S57. Accelerated aqueous decomposition of MAX12 in boiling water.

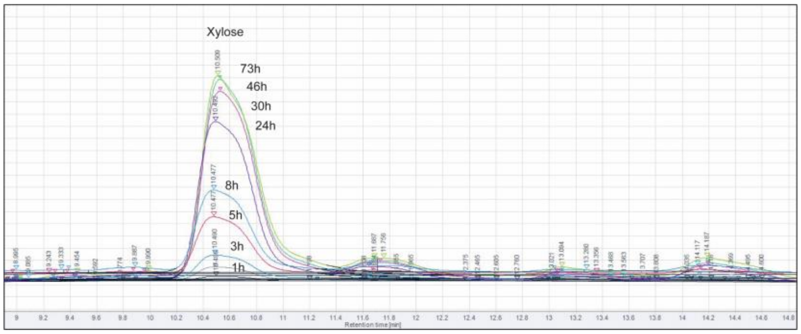

Figure S58. HPLC Analysis of xylose increase over time.

8.9.2 Biodegradability measurements

The ultimate biodegradability test was done following the OECD guideline 301 F protocol (Manometric Respirometry). The OxiTop® measuring system was used for the BOD measurement based on the pressure difference measurement. Mineral medium was defined as pH 7.4 phosphate buffer solution with micronutrients (Ca^{2+}, Mg^{2+}, Fe^{3+}). Polyseed® microbial BOD capsules were used to provide an initial

microbial population. Known quantities of the MAX12 Mix and xylose (procedure control) were introduced in sealed bottles (Oxitop®) for a period of 28 days at a temperature of 20 ± 1 °C in the dark. Meanwhile, an inoculum blank was also tested under the same conditions. Each test was repeated in duplicate. The biodegradability of the test substance was caluculated with Equation S7 and S8. The BOD of the test substance was calculated as the difference between the oxygen uptake by the test substances and by inoculum blank after the 28-day incubation period, which was then divided by the weight of the test chemical used. Dividing the BOD by the theoretical oxygen demand (ThOD, see definition below) is the percentage biodegradation.

Equation S7:

$$BOD = \frac{O_2\ uptake\ by\ test\ substance\ \left(\frac{mg}{L}\right) - O_2\ uptake\ by\ blank\ \left(\frac{mg}{L}\right)}{Test\ substance\ in\ vessel\ \left(\frac{mg}{L}\right)} = mg\ O_2/mg\ test\ substance$$

Equation S8:

$$\%\ biodegradation = \frac{BOD\ (mg\ O_2/\ mg\ test\ substance)}{ThOD\ (mg\ O_2/\ mg\ test\ substance)} \times 100\%$$

The theoretical oxygen demand (ThOD) of xylose is 1.066 mg/mg, the ThOD of MAX12 ($C_{17}H_{32}O_5$) is 2.275 mg/mg. (ThOD is the total amount of oxygen required to oxidize a chemical completely; it is calculated from the molecular formula. The ThOD of a $C_cH_hO_o$ formula is $16(2c+1/2h-o)$/Molecular weight (OECD guideline 301).)

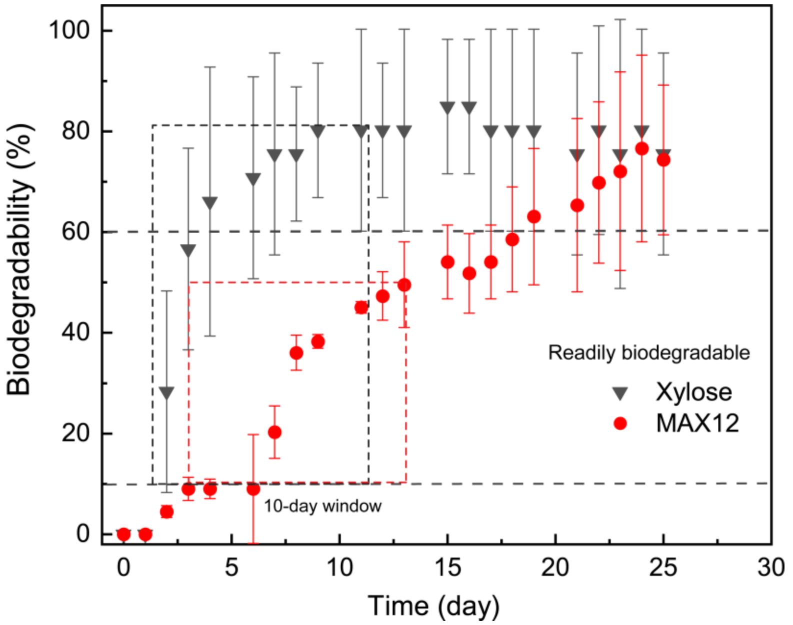

Figure S59. Biodegradability profile for MAX12 and xylose.

8.10 Toxicity of GMAX

To further understand the toxicity of this new class of surfactants, we selected GMAX as the representative molecule on which additional tests were conducted. The standard tests following ISO standards included the general toxicity by measuring the bioluminescence inhibition to *Aliivibrio fischeri* (ISO 11348-3: 2007), the herbicidal effects by the combined algae test using *Raphidocelis subcapitata* (ISO 8692: 2004), and the estrogenic activity with the lyticase yeast estrogen screen (ISO 19040-1: 2018). The detailed measurement procedures are detailed in the respective ISO documents. Briefly, GMAX was pre-dissolved in ethanol at a 10 mg/mL concentration to improve its solubility before diluting 100 times with nanopure water as a 0.1 mg GMAX/mL stock solution. Nanopure water with 1% ethanol was used as the blank in the corresponding test. The stock solution was diluted to different concentrations with nanopure water for each test. The inhibition of *Aliivibrio fischeri* bioluminescence was measured by exposing the bacterial culture to the GMAX solution and the blank at various concentrations for 30 min. The bioluminescence emitted from the bacterial culture was then measured using a luminometer in comparison to the original bacterial culture before adding the assay. The combined algae test considers both the growth inhibition and the inhibition of photosynthesis (i.e., photosystem II). The tested samples were added to a culture of *Raphidocelis subcapitata* before incubation at 23°C for 2h for the photosynthesis inhibition test and 24h for the growth inhibition test. Diuron, a known herbicide and algaecide, was used as the positive control. The cell density was measured by light absorbance at 685nm with a spectrometer, which was an indicator of the algal growth. The photosynthetic activity was measured by the quantum yield with the imaging pulse amplitude modulation. The lyticase yeast estrogen screen used *Saccharomyces cerevisiae* as the test organism that contained human estrogen receptor and a reporter gene that indicated estrogenic effects by changing the colour of the culture. 17β-estradiol, a potent form of estrogen in the human body, was used as the positive control. The yeast culture was inoculated with the tested samples at various concentrations before an 18h incubation. The cells were lysed and the colour of the culture was measured by a spectrometer. The test results are summarized in Figures S60-S62.

GMAX showed no bioluminescence inhibition of Aliivibrio fischeri, which served as a preliminary indicator for the absence of general cytotoxicity. The negative estrogenic activity of GMAX at any tested concentration further highlights its benignness. On the other hand, while GMAX did not negatively affect the photosynthesis of *Raphidocelis subcapitata* at any tested concentrations after a 2h exposure, it started to inhibit algal growth at a concentration above 1 mg/L, with an EC10 of 6.3 mg/L. However, this induction concentration is about an order of magnitude higher than what is commonly found in commercial surfactants, which suggests that GMAX may have a lower herbicidal and algaocidal activity than common commercial surfactants, like SDS and LAS[23,26]. All surfactants tend to have some inhibitory effect due to their innate surface activity and their interactions with cell membranes[23–25]. The combined algae test with

24 h incubation only served as a speed screening in this preliminary assessment. An extended test period should be considered to assess GMAX's full growth effects. Other surfactants synthesized from this scheme and their degradation intermediates should also be tested in future studies to fully understand the impacts of these molecules on the environment and human health.

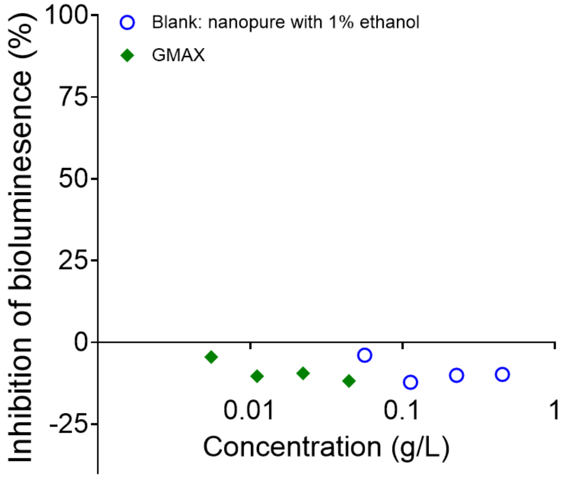

Figure S60. Effects on bioluminescence in bacteria following exposure to GMAX (green) and the blank (blue).

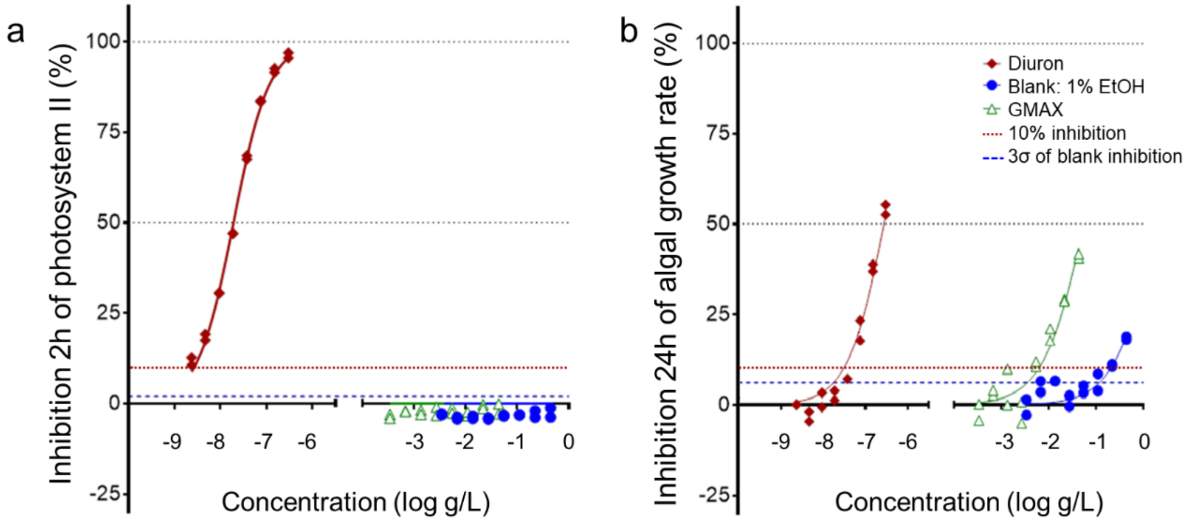

Figure S61. Concentration-effect relationships of diuron (red, positive control), GMAX (green) and blanks (blue) in the combined algae test, (a) the photosynthesis inhibition and (b) the algal growth inhibition. The red dotted line corresponds to the 10% inhibition (EC10). The blue dashed line represents 3 times the average standard deviation (3σ) of the blank test run in triplicate.

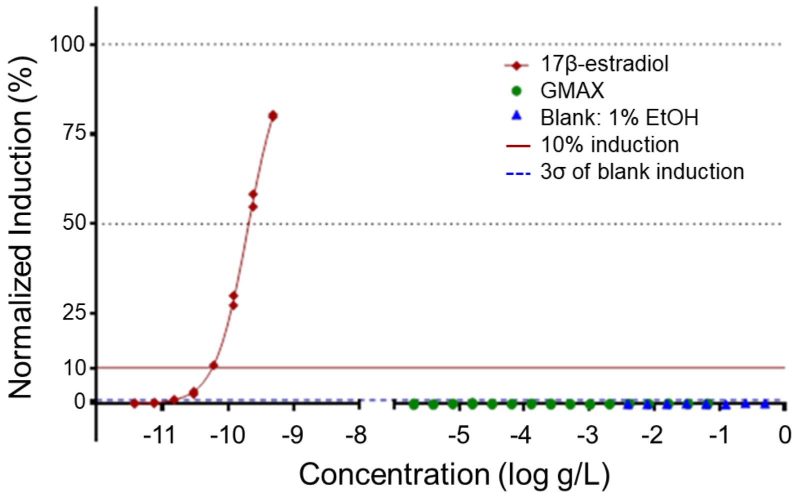

Figure S62. Concentration-effect on the estrogen activity of 7β-estradiol induced (red, positive control), GMAX (green), and blank (blue). The red line corresponds to 10% inhibition (EC10). The blue dashed line represents the 3 times the average standard deviation (3σ) of the blank test run in triplicate.

Reference

[1] C. Gozlan, E. Deruer, M.-C. Duclos, V. Molinier, J.-M. Aubry, A. Redl, N. Duguet, M. Lemaire, *Green Chem.* **2016**, *18*, 1994–2004.

[2] J. Kops, H. Spanggaard, *Die Makromol. Chemie* **1975**, *176*, 299–313.

[3] R. A. Morris, R. L. Chapman, *J. Air Pollut. Control Assoc.* **1961**, *11*, 467–489.

[4] W. Lan, J. B. de Bueren, J. S. Luterbacher, *Angew. Chemie* **2019**, *131*, 2675–2680.

[5] A. L. Fameau, J. Ventureira, B. Novales, J. P. Douliez, *Colloids Surfaces A Physicochem. Eng. Asp.* **2012**, *403*, 87–95.

[6] A. G. Pelmenschikov, R. A. Van Santen, J. Jánchen, E. Meijer8, *CD3CN as a Probe of Lewis and Bronsted Acidity of Zeolites*, **1993**.

[7] M. Thommes, K. Kaneko, A. V. Neimark, J. P. Olivier, F. Rodriguez-Reinoso, J. Rouquerol, K. S. W. Sing, *Pure Appl. Chem.* **2015**, *87*, 1051–1069.

[8] P. Tarazona, *Phys. Rev. A* **1985**, *31*, 2672–2679.

[9] P. Tarazona, U. M. B. Marconi, R. Evans, *Mol. Phys.* **1987**, *60*, 573–595.

[10] W. Bi, N. Mercier, *Chem. Commun.* **2008**, 5743.

[11] S. Li, A. Zheng, Y. Su, H. Fang, W. Shen, Z. Yu, L. Chen, F. Deng, *Phys. Chem. Chem. Phys.* **2010**, *12*, 3895–3903.

[12] H. M. Kao, C. P. Grey, *J. Phys. Chem.* **1996**, *100*, 5105–5117.

[13] Z. Zhao, D. Xiao, K. Chen, R. Wang, L. Liang, Z. Liu, I. Hung, Z. Gan, G. Hou, *ACS Cent. Sci.* **2022**, *8*, 795–803.

[14] K. Chen, S. Horstmeier, V. T. Nguyen, B. Wang, S. P. Crossley, T. Pham, Z. Gan, I. Hung, J. L. White, *J. Am. Chem. Soc.* **2020**, *142*, 7514–7523.

[15] K. Chen, Z. Gan, S. Horstmeier, J. L. White, *J. Am. Chem. Soc.* **2021**, *143*, 6669–6680.

[16] R. D. Shannon, K. H. Gardner, R. H. Staley, G. Bergeret, P. Gallezot, A. Auroux, *J. Phys. Chem.* **1985**, *89*, 4778–4788.

[17] S. R. Batool, V. L. Sushkevich, J. A. van Bokhoven, *J. Catal.* **2022**, *408*, 24–35.

[18] M. Ravi, V. L. Sushkevich, J. A. van Bokhoven, *Chem. Sci.* **2021**, *12*, 4094–4103.

[19] D. Massiot, F. Fayon, M. Capron, I. King, S. Le Calvé, B. Alonso, J. Durand, B. Bujoli, Z. Gan, G. Hoatson, *Magn. Reson. Chem.* **2002**, *40*, 70–76.

[20] M. Niwa, N. Katada, *Chem. Rec.* **2013**, *13*, 432–455.

[21] F. Lónyi, J. Valyon, *Microporous Mesoporous Mater.* **2001**, *47*, 293–301.

[22] H. G. Karge, in *Catal. Adsorpt. by Zeolites*, **1991**, pp. 133–156.

[23] F. Lónyi, J. Valyon, *Thermochim. Acta* **2001**, *373*, 53–57.

[24] J. Jae, G. A. Tompsett, A. J. Foster, K. D. Hammond, S. M. Auerbach, R. F. Lobo, G. W. Huber, *J. Catal.* **2011**, *279*, 257–268.

[25] E. Sjöman, M. Mänttäri, M. Nyström, H. Koivikko, H. Heikkilä, *J. Memb. Sci.* **2007**, *292*, 106–115.

[26] R. B. Bird, W. E. Stewart, E. N. Lightfoot, *Transport Phenomena*, New York, **2002**.

[27] “Chemeo database,” can be found under https://www.chemeo.com/, **n.d.**

[28] C. Baerlocher, L. B. McCusker, “Database of Zeolite Structures,” **1996**.

[29] W. Song, H. Liu, J. Zhang, Y. Sun, L. Peng, *ACS Catal.* **2022**, *12*, 12833–12844.

[30] N. Wang, M. Zhang, Y. Yu, *Microporous Mesoporous Mater.* **2013**, *169*, 47–53.

[31] N. Pfriem, P. H. Hintermeier, S. Eckstein, S. Kim, Q. Liu, H. Shi, L. Milakovic, Y. Liu, G. L. Haller, E. Baráth, Y. Liu, J. A. Lercher, *Science (80-.).* **2021**, *372*, 952–957.

[32] S. Sultana Poly, S. M. A. Hakim Siddiki, A. S. Touchy, S. Yasumura, T. Toyao, Z. Maeno, K. ichi Shimizu, *J. Catal.* **2018**, *368*, 145–154.

[33] E. Pérez-Botella, S. Valencia, F. Rey, *Chem. Rev.* **2022**, *122*, 17647–17695.

[34] S. U. Rege, J. Padin, R. T. Yang, *AIChE J.* **1998**, *44*, 799–809.

[35] C. F. Fellow, *Proc. R. Soc. London. Ser. B - Biol. Sci.* **1937**, *122*, 155–174.

[36] S. Molina-Gutiérrez, V. Ladmiral, R. Bongiovanni, S. Caillol, P. Lacroix-Desmazes, *Ind. Eng. Chem. Res.* **2019**, *58*, 21155–21164.

[37] N. Ferlin, D. Grassi, C. Ojeda, M. J. L. Castro, A. Fernández-Cirelli, J. Kovensky, E. Grand, *J. Surfactants Deterg.* **2012**, *15*, 259–264.

[38] USGS-U.S. Geological Survey Office of Water Quality, “Hardness of Water,” can be found under https://www.usgs.gov/special-topics/water-science-school/science/hardness-water, **2018**.

[39] M. E. Mahmoud, M. K. Obada, *Chem. Eng. J.* **2014**, *252*, 355–361.

[40] E. Illous, S. Doblinger, S. Pipolo, J. F. Ontiveros, R. Lebeuf, J.-M. Aubry, *J. Colloid Interface Sci.* **2021**, *585*, 808–819.

[41] C. Xu, H. Wang, D. Wang, Y. Zhu, X. Zhu, H. Yu, *Colloids Surfaces A Physicochem. Eng. Asp.* **2021**, *613*, 126046.

[42] Y. Chen, G. Xu, *Colloids Surfaces A Physicochem. Eng. Asp.* **2013**, *424*, 26–32.

[43] L. Rhein, in *Handb. Cleaning/Decontamination Surfaces*, Elsevier, **2007**, pp. 305–369.

[44] D. S. Park, K. E. Joseph, M. Koehle, C. Krumm, L. Ren, J. N. Damen, M. H. Shete, H. S. Lee, X. Zuo, B. Lee, W. Fan, D. G. Vlachos, R. F. Lobo, M. Tsapatsis, P. J. Dauenhauer, *ACS Cent. Sci.* **2016**, *2*, 820–824.

[45] A. Al Ghatta, R. C. Aravenas, Y. Wu, J. M. Perry, J. Lemus, J. P. Hallett, *ACS Sustain. Chem. Eng.* **2022**, *10*, 8846–8855.
